# Automatic value learning results in counterproductive human behavior

**DOI:** 10.1038/s41467-026-76297-7

**Published:** 2026-08-10

**Authors:** Ido Ben-Artzi, Maayan Pereg, Roy Luria, Rani Moran, Nitzan Shahar

**Affiliations:** 1 School of Psychological Sciences, Tel Aviv University, Tel Aviv, Israel; 2 Sagol School of Neuroscience, Tel Aviv University, Tel Aviv, Israel; 3 Department of Psychology, Achva Academic College, Yinon, Israel; 4 Department of Psychology, School of Biological and Behavioural Sciences, Queen Mary University of London, London, UK; 5 Max Planck UCL Centre for Computational Psychiatry and Ageing, University College London, London, UK

**Keywords:** Human behaviour, Reward, Learning algorithms

## Abstract

Humans are adept at accurately estimating the value of available choices from accumulated experience. However, cognitive processing also incorporates irrelevant information during deliberation, undermining decision accuracy. Here, we show that credit assignment operates automatically, allowing irrelevant action features to acquire value and impair choice behavior. Across four preregistered experiments (*N* = 504, 158, 195, 319) and a re-analysis of previously published data (*N* = 50), we examine outcome-irrelevant learning to spatial-motor and visual action features under conditions of explicit instruction and extensive training. In all cases, automatic value updating persists even when participants clearly understood which features were non-predictive of reward, and reliance on these irrelevant values led to suboptimal choices. Moreover, working memory capacity, known as a key regulatory resource, predicts the magnitude of this automatic outcome-irrelevant learning. Collectively, our results broaden the concept of automatic cognitive processing beyond the selective-attention literature to encompass reinforcement learning mechanisms.

## Abstract (web summary)

Humans use rewards to learn which actions are worth repeating. Here, the authors demonstrate an automatic tendency to credit unrelated actions for these rewards, driving suboptimal choices.

## Introduction

To effectively achieve their goals, humans need to identify which actions lead to desired outcomes. Commonly, humans make decisions in complex situations where it can be challenging to determine what specific aspects of their behavior led to the intended outcome^1–4^. This challenge, termed the “credit assignment problem”, plays a major role in the human reinforcement learning literature^5–8^. Research has shown that to address this challenge, humans are able to accurately assign credit by forming an internal representation of causal dependencies in the environment, either based on prior knowledge or by gradually inferring, through trial-and-error, which features of their actions reliably predict reward^9–12^. However, studies have also shown that humans assign credit to outcome-irrelevant features of their actions^13–21^. Thus, a fundamental question is whether knowledge about the causal structure of the environment, either provided through instructions or inferred through experience, leads humans to fully disregard outcome-irrelevant information.

By learning from reinforcement, humans can make decisions based on previously accumulated knowledge and experience^22–24^. However, it is not clear whether the human learning system can effectively ignore associating actions and outcomes that appear in close temporal proximity yet are not causally related. For example, consider a child picking an apple from a tabletop. Both the child’s prior knowledge and experience could guide the child to attribute feedback from consuming the apple (e.g., its taste) to relevant features such as its color. However, in a scenario where the environment’s underlying causal structure is not represented in a direct manner, the learning system might struggle to withhold assigning credit to incidental characteristics like the apple’s location on the tabletop. Previous work suggested that the human brain assigns credit from rewarding outcomes to incidental and non-causally related representations^25,26^. However, demonstrations of such non-causal credit assignment have been largely confined to credit spreading within the outcome-relevant feature^27,28^, for example, assigning credit to actions that fall outside the causal time window^25^ or to spatially adjacent actions despite explicit knowledge of the task’s spatial structure^26^. Our work extends this literature by showing an automatic credit assignment even when a clear dimensional separation exists between information relevant and irrelevant to the outcome, allowing, in principle, the complete filtering of outcome-irrelevant information during credit assignment. Thus, we argue that the human value-based learning system automatically forms action-outcome associations between action features and an outcome, even when both prior knowledge and extensive experience indicate that those action features are irrelevant to the outcome, and despite leading to reduced gains.

Automatic cognitive processing has been defined as a mental operation triggered by irrelevant environmental cues that persist despite impairing task performance^29–32^. Accordingly, automatic cognitive processes have been well documented in studies on selective attention and cognitive control, demonstrating that task-irrelevant information consistently influences human decision-making, even when it leads to reduced performance^33–36^. However, despite a longstanding emphasis on automatic processing in the cognitive control literature, the role of automaticity in human reinforcement learning has been understudied and remains poorly understood^37–39^. Reinforcement learning studies often assume that high-level representations of task state fully constrain credit assignment, shielding it from irrelevant influences^9,40–42^. Consequently, the possibility that outcome-irrelevant information influences behavior in a state-independent manner was mostly neglected.

To evaluate our claim, several methodological and theoretical hurdles must be addressed with care. As a first step, it is essential to ensure that bona-fide instructions are delivered clearly and enable participants to accurately represent which task features are relevant to predicting outcomes^9,43,44^. In a pivotal study, Feher da Silva & Hare suggested caution when interpreting learning estimates, showing they can sometimes reflect poor instruction delivery, leading to the formation of a wrong model of the task’s environment. Specifically, Feher da Silva and Hare^45,46^ demonstrated that embedding reinforcement learning tasks within engaging and clear narratives (e.g., spacecrafts visiting planets and magic carpets reaching mountains) helps participants follow instructions effectively and dramatically minimizes model-free influence on choice behavior. In contrast, previous reports of outcome-irrelevant learning were based on abstract stimuli and instructions, thus not meeting the benchmark of a narrative-based instruction^13–19,40^. Therefore, it is yet unknown whether outcome-irrelevant learning reflects an automatic property of learning, or rather poor instruction delivery leading to the formation of wrong models of the task’s environment.

A second important consideration is that even if verbal instructions are insufficient to prevent outcome-irrelevant learning, participants may still rely on extensive experiential learning to infer the outcome-relevance of features^3,12,47,48^. For example, prior research has indicated feature-specific attention weights that track each feature’s predictive value for rewards^4,49–52^. Consequently, if outcome-irrelevant learning bears automatic qualities, it should still be observed even after a prolonged trial-and-error period, enabling participants to infer through experience which action features should be disregarded^53^.

Third, it is important to determine the extent to which outcome-irrelevant learning reflects a domain-general phenomenon. Prior studies demonstrating outcome-irrelevant learning have been largely confined to the spatial-motor domain^13,17,18^, raising the question of whether automatic credit assignment also occurs in other domains. If outcome-irrelevant learning operates automatically, then all action features activated as part of the task set should, in principle, receive credit. During action selection in a reinforcement-learning context, all motor and perceptual features required to execute a choice are activated as part of the task set^54–56^. One possibility is that all features activated during action selection, regardless of domain, are retrospectively attended to at outcome delivery and are therefore assigned credit, even though they are known to be irrelevant for predicting reward^57^. Alternatively, spatial-motor features may hold a privileged status due to their central role in natural environments, making credit assignment to locations or motor actions difficult to suppress even when they are known to be non-predictive of reward^58,59^.

Finally, even if outcome-irrelevant learning is domain-general and persists despite bona fide instructions and prolonged practice, studies on decision-making suggest that some individuals may be better able to regulate the influence of automatic processes than others^60,61^. Specifically, working memory capacity has been established as a resource that facilitates the activation and maintenance of instructed representations that guide decision-making and learning^62,63^, e.g., by reducing susceptibility to interference from irrelevant information^64–66^. We therefore further examined the association between working memory capacity and the ability to regulate outcome-irrelevant learning.

In the present study, we collected data from four preregistered experiments using a multi-armed bandit reinforcement learning task, in which participants made choices to maximize rewards. Crucially, across experiments, participants were explicitly told which feature predicted rewards, and which were outcome-irrelevant (i.e., locations in Experiments 1,2, and 4A; colors in Experiments 3 and 4B). In Experiment 1 (*N* = 504), we observed robust evidence of automatic outcome-irrelevant learning based on stimulus location despite a story-based instructional framing. To further validate this finding, we reanalyzed the story-based dataset from Feher da Silva et al. (*N* = 50^45^) and found a consistent pattern, replicating our results in a two-step reinforcement learning task. Next, in Experiment 2, we addressed the question of whether outcome-irrelevant learning will diminish with practice. Here, participants (*N* = 158) performed the task across three sessions in three consecutive days, allowing time to learn the irrelevant nature of stimulus locations from experience. Nevertheless, we found that value learning for outcome-irrelevant spatial-motor features persists despite leading to reduced performance and gains. To examine whether this automatic credit assignment is specific to the spatial-motor domain, in Experiment 3 (*N* = 195), we flipped the outcome-relevance, making locations outcome-relevant and colors outcome-irrelevant, and found that the effect generalizes beyond the spatial-motor domain. To test whether our findings could be explained by a Pavlovian association formed during outcome presentation between the irrelevant feature and the outcome, rather than by a credit assignment mechanism, we conducted Experiment 4. We replicated the effect while controlling for Pavlovian associations, demonstrating that outcome-irrelevant learning remains whether the location (Exp. 4 A, *N* = 129) or the color (Exp. 4B, *N* = 190) served as the irrelevant feature. This allowed us to conclude that the effect is driven by credit assignment to outcome-irrelevant action features rather than by Pavlovian associations alone. Finally, we show that, in line with the automaticity literature, working memory capacity is negatively associated with outcome-irrelevant learning, with lower capacity linked to stronger learning for outcome-irrelevant features.

Reinforcement learning studies have highlighted a remarkable human ability to efficiently guide their behavior according to the structure of the environment^8,67–69^. Yet, automatic cognitive processing has received less emphasis within the human credit assignment literature^39^. This work highlights a human tendency toward outcome-irrelevant learning, which leads to suboptimal performance and reduced gains. The robustness of this aspect of human learning should therefore be given greater consideration when developing models of human decision-making.

## Results

**Fig. 1:**
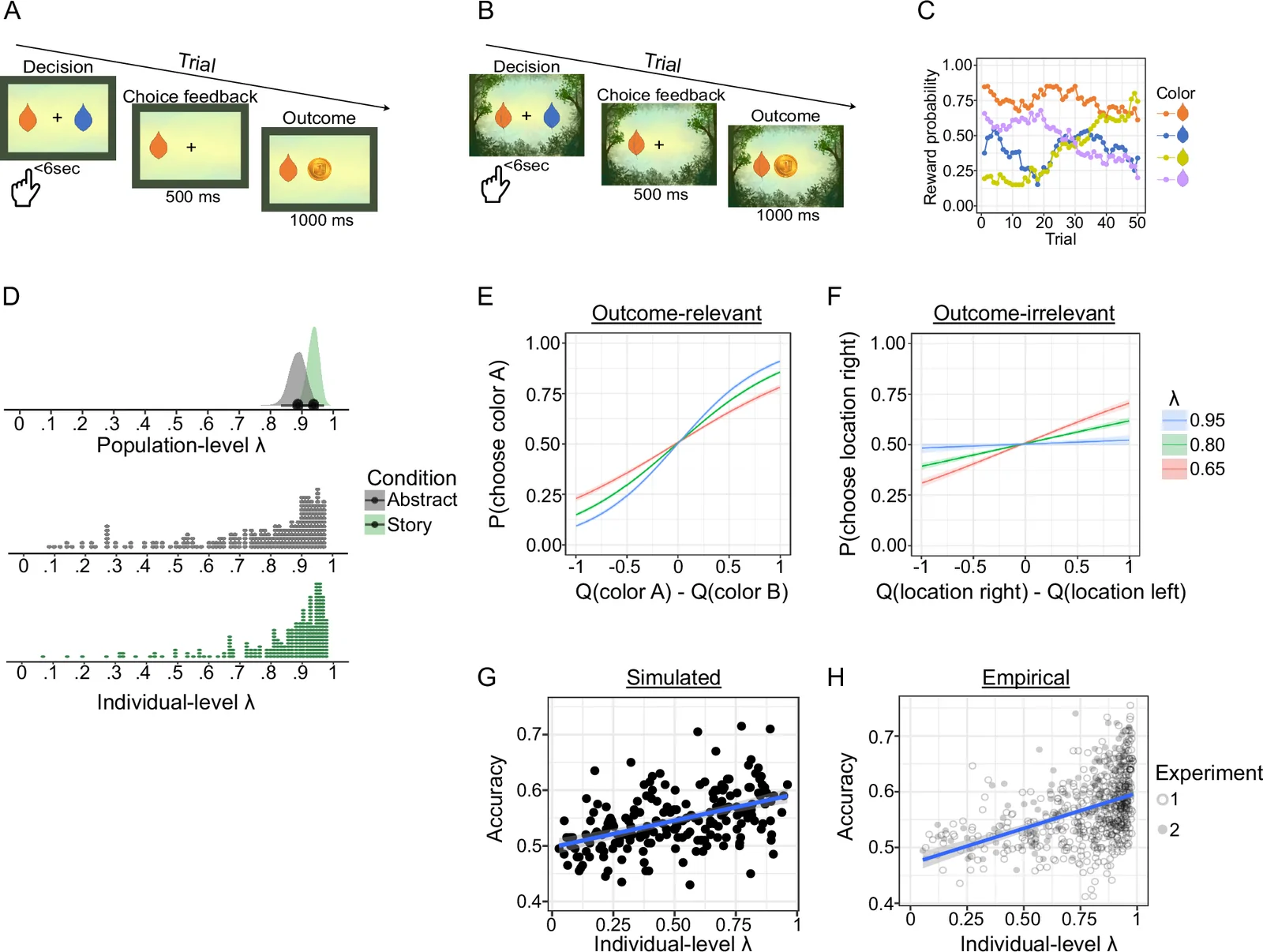
Outcome-irrelevant learning persists even when using story framing. We examined whether humans assign credit to action features that were explicitly instructed as outcome-irrelevant, using two versions of a multi-armed bandit task that differed in their framing^46^. **A** In the abstract condition, participants completed a standard task in which colored shapes determined reward probabilities. **B** In the story condition, the same task was framed as a “Magical Forest,” where participants were told that a wizard wanted to buy colored leaves for his potion. **C** On each trial, participants selected a color and received a binary outcome, either a reward, + 1, or no reward, 0. Rewards were determined by color-specific probabilities that drifted slowly and randomly across the 50 trials in each block. Each block introduced new colors, requiring participants to learn and continuously update their preferences from observed outcomes. **D** We modeled choices using a feature-specific prediction error model that estimated learning about both outcome-relevant and outcome-irrelevant action features. The key parameter, λ, quantified the extent to which choices were driven by relevant feature values rather than irrelevant feature values. Across both conditions, population-level λ estimates were substantially below 1, indicating a consistent influence of outcome-irrelevant learning on choice. Colored circles (gray for abstract and green for story conditions) show posterior medians for each condition, horizontal lines show 95% highest density intervals (HDI_95%_), and individual dots show participant-level λ estimates. **E** Model-derived trial-by-trial Q-values showed that higher λ values were associated with greater reliance on outcome-relevant color values. Colors represent different empirical λ values. Shaded bands represent the 95% credible interval (CI). **F** Conversely, lower λ values reflected stronger outcome-irrelevant learning, expressed as greater reliance on values erroneously assigned to spatial locations. Colors represent different empirical λ values. Shaded bands represent the 95% CI. **G** Simulations across a broad parameter range showed that higher λ, reflecting weaker outcome-irrelevant learning, led to higher accuracy, defined as choosing the color with the currently higher expected value. Each black dot represents a simulated agent, and the regression line is depicted in blue with the shaded band representing the 95% CI. **H** Empirical data from Experiments 1&2 supported this pattern, showing that stronger outcome-irrelevant learning was associated with lower accuracy and smaller monetary bonuses. Empty dots represent data from Experiment 1 (*N* = 504; pooled across both conditions), filled dots represent data from Experiment 2 (*N* = 158; see below), and the regression line is depicted in blue with the shaded band representing the 95% CI.

Across four preregistered experiments, participants completed a modified version of the multi-armed bandit task. In Experiment 1, 504 participants completed the task under an “abstract” or “story” framing of instructions. On each trial, participants were given a choice between two options, randomly selected from a set of four stimuli, each represented by a color. Upon selecting one of the two options, participants observed an outcome of either a golden coin (indicating a reward) or a black coin (indicating no reward; see Fig. 1A, B). To motivate performance, coins were later translated into real monetary bonuses. Each of the four stimuli (i.e., colors) had an associated probability of yielding a reward, which slowly fluctuated over time following a random walk process (see Fig. 1C). Participants were uninformed of these probabilities and were expected to adapt their choices based on outcome feedback to maximize their earnings. Crucially, the spatial location of the stimuli on the screen (left or right) was randomized on each trial, rendering location non-informative for predicting outcomes. Participants were explicitly told to base their choices on the outcome-relevant visual attribute (i.e., color) and to ignore the outcome-irrelevant spatial feature (i.e., locations of the stimuli). To confirm understanding, all participants completed a comprehension quiz, which had to be answered perfectly before beginning the task. Accordingly, we defined learning based on the colors as “outcome-relevant learning,” while learning associated with spatial location was termed “outcome-irrelevant learning”. Importantly, basing decisions on values assigned to outcome-irrelevant locations can only result in suboptimal performance in this task, as it bears no predictive value and introduces noise into the internal value representations (see Fig. 1).

### Computational modeling

We used computational modeling to disentangle the contribution of outcome-irrelevant learning estimates from other factors influencing choice behavior. Specifically, we implemented a reinforcement learning algorithm in which the value of each visual stimulus and each spatial location was updated separately using a feature-specific prediction error algorithm^49^. Crucially, the model included a free parameter, λ, which integrated the values of features relevant to the outcome with those irrelevant to the outcome. The λ parameter was constrained to range between 0 and 1, where a value of 1 indicated that choices were based entirely on the value of the offered visual stimuli, a value of 0.5 reflected equal weighting of stimuli and location, and a value of 0 meant that choices were based solely on the incidental location value. By allowing λ to vary freely within this range, the model captured the extent to which participants relied on outcome-relevant versus outcome-irrelevant information in their value-based decisions. Finally, the model incorporated separate perseveration mechanisms for visual and spatial features, capturing the tendency to repeat (or switch) prior selections of each feature regardless of outcome. Therefore, this modeling framework allowed us to dissociate value-based decision-making driven by outcome-irrelevant learning from behavior influenced by outcome-relevant learning and habitual/explorative tendencies.

**Fig. 2:**
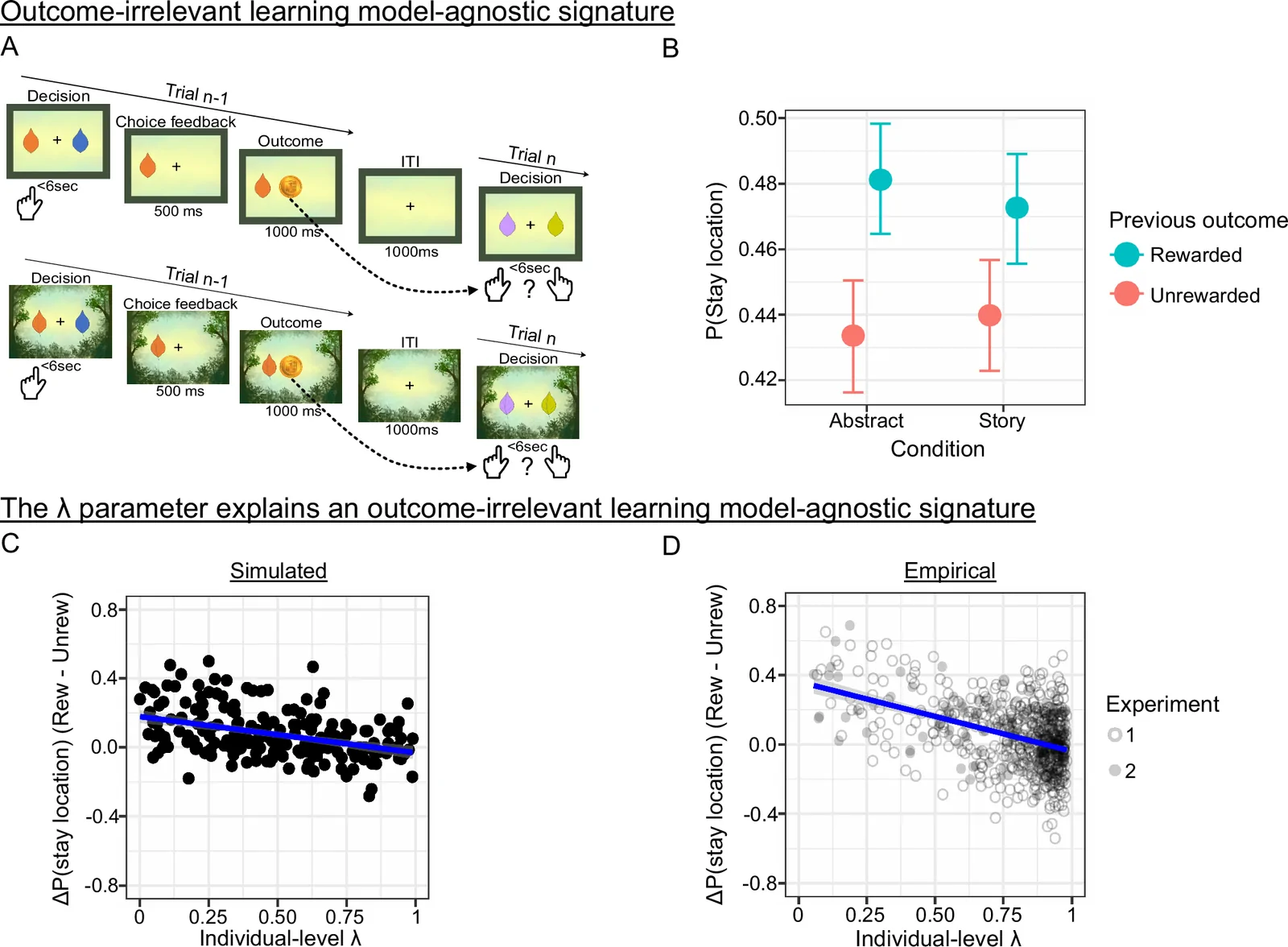
Model-agnostic signature indicated outcome-irrelevant learning. To confirm that participants learned the value of outcome-irrelevant locations, we estimated a model-agnostic signature. **A** We focus on a trial sequence where colors offered on trial *n-1* were not reoffered on trial n, allowing us to examine the model-agnostic effect of the outcome in trial *n-1* on trial *n* location choice, without any interfering contribution from outcome-relevant learning. Note that according to current literature of human reinforcement learning, the outcome in trial *n-1* should have no influence on the trial *n* choice, as two different colors are offered for selection. **B** We fitted a hierarchical Bayesian logistic regression model examining the influence of the previous outcome (from trial n-1) on the tendency to choose the same location (in trial n) when two different colors are offered (see panel A). We found that in both conditions (Abstract: *N* = 250; Story: *N* = 254), outcome-irrelevant learning tendencies are apparent, as indicated by a higher probability (~ 4%) of staying with the same location choice after a rewarded trial compared to an unrewarded one. The below chance intercept (middle point between unrewarded and rewarded trials) reflects a location-switching tendency as we illustrate further in Fig. 3C, D. Colored circles represent the posterior median, and the error bars represent the 95% CI. **C** We simulated data using our model and task design to illustrate the relationship between the model-agnostic behavioral signature and the λ parameter. Y-axis values indicate for each simulated agent the difference in the probability of repeating the same location choice (when two different cards than before are offered, see panel **A**) following rewarded vs. unrewarded trials. As expected, agents generated using lower λ values show a bigger positive effect. Each black dot represents a simulated agent, and the regression line is depicted in blue with the shaded band representing the 95% CI. **D** We repeat this analysis for empirical data from Experiments 1 & 2, showing the negative relationship between λ and the model-agnostic behavioral signature. Empty dots represent data from Experiment 1 (*N* = 504) and filled dots represent data from Experiment 2 (*N* = 158). The regression line is depicted in blue with the shaded band representing the 95% CI.

To examine the contribution of including outcome-irrelevant learning to predicting out-of-sample human choice behavior, we constructed a reduced model that was identical to the full version mentioned above, but excluded choosing based on the location feature by fixing λ at 1. Both the full and reduced models were built within a hierarchical framework that captures variability at the group and individual levels. Each model was estimated through Bayesian posterior inference with MCMC sampling in Stan^70,71^, and evaluated using out-of-sample predictive performance^72^. Prior to analyzing empirical data, we conducted three simulation-based validation steps aligned with established computational modeling standards^73^. First, we completed a model recovery test in which we generated synthetic datasets from each model and evaluated their predictive performance using expected log predictive density (*elpd*^72,74,75^). Results demonstrated strong discriminability between the full and reduced models based on posterior predictive performance (see “Methods”). Second, we assessed parameter recoverability in the full model, observing high recovery across both population and subject-level parameters (see “Methods”). Third, we showed that the λ parameter maps meaningfully onto a model-agnostic regression signature based on a subset of trials, allowing us to further validate outcome-irrelevant estimates outside the realm of computational modeling^76^ (see Fig. 2). Together, these validation steps confirmed that the models demonstrated strong recovery characteristics, robust parameter identifiability, and a clear model-agnostic behavioral signature of outcome-irrelevant learning.

### Outcome-irrelevant learning persists regardless of how instructions are framed

Previous work has shown that participants tend to assign value to locations, even when explicitly instructed about their irrelevance for outcome prediction^13^. However, embedding instructions within a narrative has been previously shown to enhance participants’ ability to follow the task’s instructions. In this study, we examined whether outcome-irrelevant learning persists when participants are provided with a story explaining why stimulus locations randomly vary from trial to trial and are thus irrelevant for predicting the outcome. To this end, in Experiment 1, participants completed a multi-armed bandit task framed as a “Magical Forest Game,” designed to facilitate the encoding and maintenance of the environment’s model^45,46^ (see Fig. 1B). The experiment employed a between-subjects design, such that participants completed either the *story* condition or a standard *abstract* version of the task. In the story condition, participants were told that in an enchanted forest, a magical tree with colored leaves exists (see Supplementary Note 12 for the full transcript). Each time, two colored leaves drop from the tree and land either to the left or right due to the random direction of the wind, as visually depicted in the instructions. A wizard living in the forest buys leaves for his potion and has hidden preferences for leaf colors, which may change throughout the game. Participants were informed that selecting a colored leaf the wizard prefers would increase their chances of receiving a gold coin in return. Critically, participants were explicitly told that the wind’s randomness makes the leaves’ locations irrelevant, as the wizard cares only about their color. Participants were instructed to make selections, observe the outcomes, and aim to maximize their rewards. To assess the effect of the story framing, participants in the abstract condition completed the exact same task with almost identical stimuli, but without the magical forest context. They were simply told that reward probability is determined by the chosen color and that location did not affect the outcome. Thus, the only difference between the two conditions was the way instructions were framed.

### Model comparison

To evaluate whether outcome-irrelevant learning explains behavior, we compared the goodness-of-fit of the full model (which includes outcome-irrelevant learning) with a reduced model (which excludes outcome-irrelevant learning), based on out-of-sample predictive choice accuracy in each condition (story and abstract; see “Methods”). In both conditions (story and abstract task framing), we found a > 99.99% chance that the full model will outperform the reduced model when predicting new data (Story Δelpd = 126, SE = 18; Abstract Δelpd = 263, SE = 24; see Supplementary Note 10 for a comparison with a simple categorical regression model^72,74,75^). This means that the full model, including learning for outcome-irrelevant locations, provides better out-of-sample choice predictions regardless of how task instructions were framed. Accordingly, we found population-level estimates of λ to be smaller than 1 in both conditions (Story: *N* = 254, λ population posterior median = .94, Highest Density Interval (HDI)_95%_ [.90, .97]; Abstract: *N* = 250, λ population posterior median = .87, HDI_95%_ [.84, .94]; see Fig. 1D), with slight evidence for a difference between conditions (λ difference posterior median = 05, HDI_95%_ [− 01, 11], pd = 95%).

### The λ parameter captures the influence of outcome-irrelevant Q-values on behavior

We next illustrate how both outcome-relevant and outcome-irrelevant learning influenced participants’ behavior using model-derived Q-value estimates. Specifically, we computed trial-by-trial posterior estimates of the Q-values for the two offered colors (relevant) and the two offered locations (irrelevant) and assessed the extent to which these values predicted participants’ choices, as a function of the individual-level λ parameter. To this end, we fitted a hierarchical Bayesian logistic regression model in which the empirical probability of choosing a given color was predicted by the Q-value difference between the two colors and its interaction with λ, as well as a second model in which the probability of choosing a given location was predicted by the Q-value difference between the two locations and its interaction with λ. As expected, higher individual λ values were associated with a stronger influence of outcome-relevant color values on choice (interaction denoting the change in the influence of the Q-value difference on choice given the change in λ estimates - posterior median on the log-odds scale = 4.49, HDI_95%_ [4.11, 4.88], pd = ~ 100%, see Fig. 1E). Conversely, higher individual λ values were associated with a weaker influence of outcome-irrelevant location values (interaction posterior median on the log-odds scale = − 2.67, HDI_95%_ [− 3.06,− 2.28], pd = ~ 100%, see Fig. 1F).

### Outcome-irrelevant learning leads to reduced accuracy and gains

To establish the suboptimality of outcome-irrelevant learning, we examined the association between each individual’s estimated λ parameter and their mean choice accuracy, defined as the proportion of trials in which participants selected the option with the currently higher expected value among the two offered options (see Fig. 1C). We first tested this association in a simulated dataset using the random walk set used for empirical testing and a broad range of parameters (see Methods). In simulation, higher λ values were robustly associated with better task performance (Simulated dataset: *N* = 200, Pearson’s r posterior median = .48, HDI_95%_ [.35, .60]; see Fig. 1G). This positive relationship was further confirmed in empirical data, where individuals with higher estimated λ values showed higher choice accuracy (Pearson’s r posterior median = .41, HDI_95%_ [.34, .49]; pooled across the two conditions; see Fig. 1H). This association translates to reduced monetary gains in both simulated and empirical datasets (see Supplementary Note 1).

### Model-agnostic regression signature for outcome-irrelevant learning

To illustrate the presence of outcome-irrelevant learning outside computational modeling, we focus on a subset of trials where the two outcome-relevant colors presented in a previous trial (trial n-1) are not reoffered in the current trial (trial n; see Fig. 2A). We aimed to demonstrate a signature of outcome-irrelevant learning, reflected in an increased tendency to repeat a previously chosen location as a function of the previous outcome (see Fig. 2B; Note that examining the tendency to repeat/switch a location as a function of outcome reflects an outcome-dependent effect, which should not be confused with outcome-independent motor perseveration extensively described in previous studies (i.e., tendency to repeat regardless of outcome^77^).

### Influence of outcome in one trial on the selection of outcome-irrelevant features in the next trial

To provide a main model-agnostic signature for outcome-irrelevant learning, we fitted a hierarchical Bayesian logistic regression estimating the effect of the *previous-outcome* (on trial *n-1*; Unrewarded/Rewarded) on the decision to repeat the same location choice on trial *n* (i.e., *stay_location*; Switch/Stay), when two different colors than before are offered (see Fig. 2A, B). We found convincing results in both abstract and story conditions indicating that the probability of repeating the same location following a rewarded trial (47%/48% for story and abstract conditions, accordingly) was consistently higher than following an unrewarded trial (44%/43% for story and abstract conditions, accordingly; Story posterior median on the log-odds scale = 0.13, HDI_95%_ between 0.04 and 0.22, pd = ~ 100%; Abstract posterior median on the log-odds scale = 0.19, HDI_95%_ between 0.1 and 0.29, pd = ~ 100%; see Fig. 2B). We found no conclusive evidence in favor of a difference between conditions (interaction posterior median = − 0.06, HDI_95%_ [− 0.19, 0.07], pd = 83%). To illustrate the relationship between the model-agnostic signature and the λ parameter from our computational model we show a clear negative correlation between these two measures in both simulated data (*N* = 200, posterior Pearson r median = − .40, HDI_95%_ [− .53, − .27], see Fig. 2C) and in empirical data (*N* = 504 pooled across the two conditions, posterior Pearson r median = − .43, HDI_95%_ [− .51, − .35], see Fig. 2D).

To further show that our model-agnostic signature reflects a reward-driven learning process, we conducted several additional analyses.

### Influence of outcomes from the past three trials on selection of outcome-irrelevant features

**Fig. 3:**
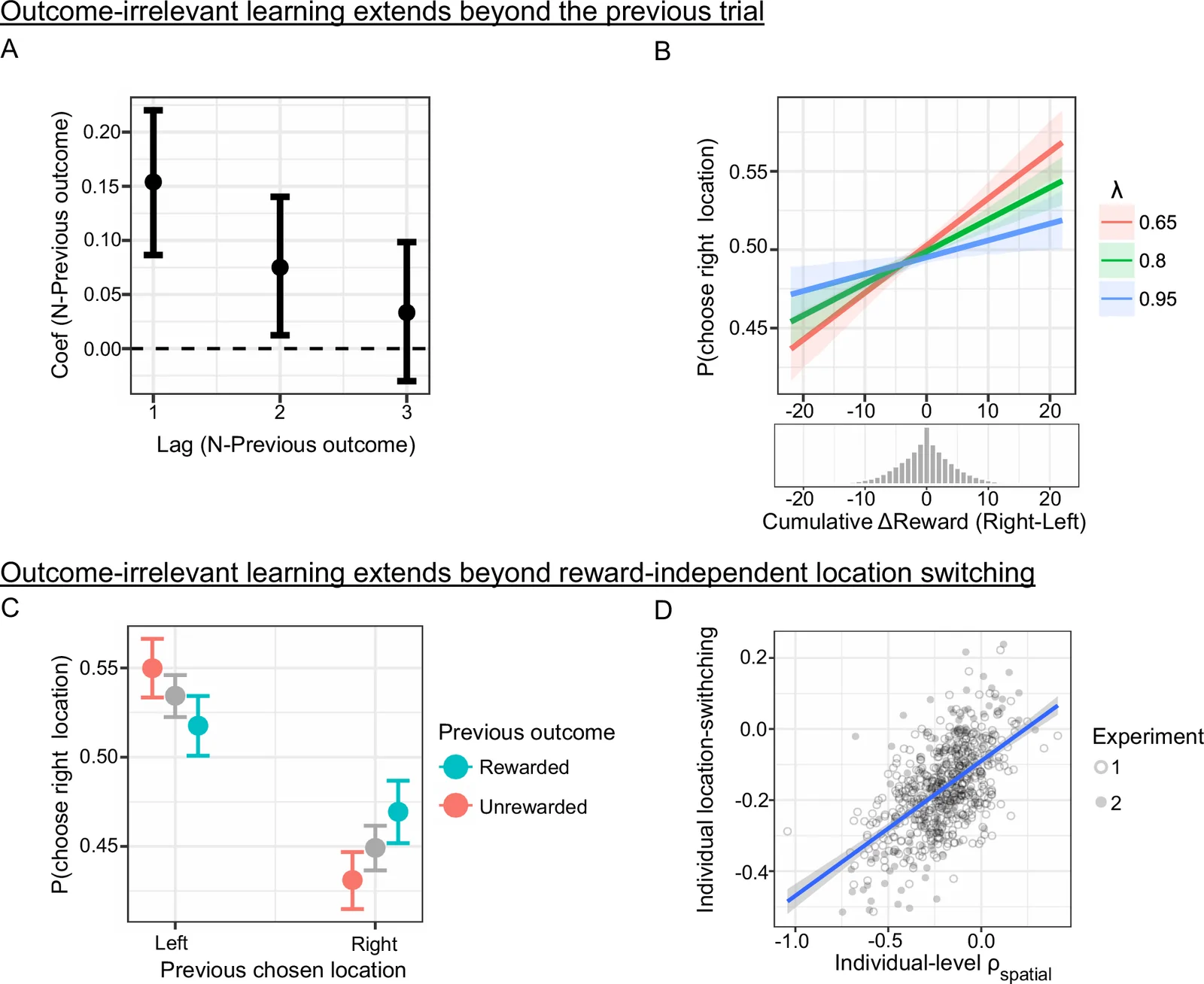
Outcome-irrelevant learning extends beyond the previous trial and is reward-dependent. **A** To show that outcome-irrelevant learning signature extends beyond the previous trial, we extend the model-agnostic regression analysis shown in Fig. 2B, from the previous trial to 2 and 3 trials back, showing a gradually decaying influence of a previously obtained reward on choice, always when different colors were now offered for selection (Data from Experiment 1; *N* = 504). Black circles represent the posterior median and error bars the 95% CI. **B** We further calculated an empirical cumulative reward difference between the two locations, while excluding the very last trial. We show that, on average, the difference is equal to 0, but when one location is by chance more rewarded than the other, choices of individuals with lower λ are strongly influenced by that arbitrary value difference. Colors represent different λ values. **C** Fig. 2B shows an intercept below chance that reflects an outcome-independent location switching tendency. To illustrate this, we provide a re-visualization of our model-agnostic result from Fig. 2B, where the chosen location (left/right) is predicted by the previously chosen location and the previous outcome. As depicted by the gray points, we show that overall participants are more likely to choose ‘left’ after choosing ‘right’ and vice versa (i.e., location switching tendency regardless of reward). Importantly, we show that choice is further influenced by the previous outcome, making participants more likely to repeat a previously chosen location after a rewarded trial, and vice versa (Data from Experiment 1; *N* = 504). Colored circles represent the reward-dependent posterior median, and error bars represent the 95% CI. **D** The explanation that the below chance intercept in Fig. 2B is due to location switching tendency independent of the obtained outcome is supported by a strong correlation of the individual model-agnostic intercept in the regression model and the ρ_spatial_ switching-perseveration parameter (*r* = .58). Empty dots represent data from Experiment 1 (*N* = 504) and filled dots represent data from Experiment 2 (*N* = 158). The regression line is depicted in blue with shaded bands showing the 95% CI.

In the analysis above (Fig. 2B), we performed a regression in which reward on trial *n-1* predicted the probability of repeating the same location on the current *n* trial. We next repeated this same regression twice more: once using reward on trial *n-2* and once using reward on trial *n-3*. Thus, we estimated the reward effect separately in three regressions (for *n-1*, *n-2*, and *n-3*). In all three analyses, we only included trials in which the historical trial (whether *n-1*, *n-2*, or *n-3*) did not contain the same colors as those offered on the current trial. We found a gradual effect of past outcomes on choice. The outcome from the immediately preceding trial had the strongest influence (posterior median = 0.15, HDI_95%_[0.09, 0.22], pd = ~ 100%), a weaker but still reliable influence remains two trials back (posterior median = 0.08, HDI_95%_[0.01, 0.14], pd = 99%), and the effect was further reduced three trials back (posterior median = 0.03, HDI_95%_[− 0.03;0.10], pd = 85%). Thus, the regression coefficients systematically decrease with temporal distance, consistent with a learning-rate-like decay in the impact of rewards over time (see Fig. 3A; see also Supplementary Fig. S2 for confirmation that these effects do not appear among artificial agents who rely only on the very recent outcome).

### Influence of cumulative reward on the selection of outcome-irrelevant features

We show that the model-agnostic cumulative reward difference between locations influences choice. Specifically, we calculated for each individual the cumulative reward difference at each point in time by adding 1 for each rewarded choice of a location and subtracting 1 for each unrewarded choice of a location. To show the effect extends beyond the very last trial, we accumulated only rewards obtained from the beginning of the block until trial n-2. We then use the difference in cumulative reward for each of the two locations to predict the next chosen location. As expected, given that locations are not predictive of outcome, on average, the cumulative reward difference between the locations was 0 (see Fig. 3B). However, we show that when one location was arbitrarily more rewarded, participants were overall more likely to choose it (posterior median = 0.03, HDI_95%_ [0.02, 0.04], pd ~= 100%), and the lower one’s estimated λ was the more they were affected by this noise in guiding their decisions (interaction posterior median = − 0.03, HDI_95%_[− 0.04, − 0.01], pd ~ = 100%; see Fig. 3B). We further illustrate that despite their randomness with respect to the outcome, the estimated difference in Q-values between the two locations varies substantially (see Supplementary Note 5).

### Perseveration estimates in the model-agnostic signature

Examination of Fig. 2B shows an intercept below chance, which reflects a tendency to switch locations regardless of outcome. To illustrate this fact, Fig. 3C provides a re-visualization of Fig. 2B. Here, instead of coding the dependent variable as the probability of staying with the previous location selection (as in Fig. 2B), we fit the same regression only predicting the chosen location (left/right coded 0 vs. 1, respectively) based on the previously chosen location (left/right) and its interaction with the previous outcome (unrewarded/rewarded). While this is a recap of Fig. 2B in terms of added information, it allows us to more clearly demonstrate that participants tend to switch their chosen location regardless of reward (see gray circles in Fig. 3C; posterior median for the effect of previously chosen location = − 0.17, HDI_95%_ [− 0.20, − 0.13], pd ~ = 100%). The figure then further demonstrates how outcome-irrelevant learning influences location selection. Specifically, after choosing the right-side location, participants were more likely to repeat that choice following a rewarded trial than an unrewarded one. In contrast, after previously choosing the left location, they were more likely to switch to the right location after an unrewarded trial than after a rewarded trial (interaction posterior median = 0.07, HDI_95%_ [0.04, 0.10], pd ~= 100%; see colored circles in Fig. 3C). Importantly, the conclusion that the regression intercept reflects a location-switching tendency is strongly supported by the negative ρ_spatial_ parameter from our computational model (*r* = .58; see Fig. 3D).

### Replication of outcome-irrelevant learning in the two-step task using previously published data

To replicate the finding of outcome-irrelevant learning despite clear, bona-fide instructions, we reanalyzed data from Feher da Silva et al. (2023). In that study, human choice behavior was examined using a story-based version of a reinforcement learning task (i.e., a two-step task). As in Experiment 1 of our study, the narrative instructions were designed to give participants a clear understanding of the task’s structure. Importantly, Feher da Silva et al. explicitly emphasized that the spatial locations of the offered options were irrelevant to reward outcomes and provided a narrative-based explanation for their arbitrary positioning.

Our complete analysis is detailed in the Supplementary Material of the current work (see Supplementary Note 3). Briefly, we found an outcome-irrelevant, model-agnostic signature, such that participants were more likely to repeat their location selection after receiving a reward compared to after an unrewarded trial, despite the clear instructions. Thus, we replicated the original outcome-irrelevant learning effect in an independent dataset, demonstrating that participants in the two-step task assign credit to spatial location, even when those locations are explicitly described as outcome-irrelevant. This finding supports the conclusion that outcome-irrelevant learning reflects an inherent component of human reinforcement learning, rather than a misunderstanding of the task structure.

### Outcome-irrelevant learning persists even following prolonged practice

**Fig. 4:**
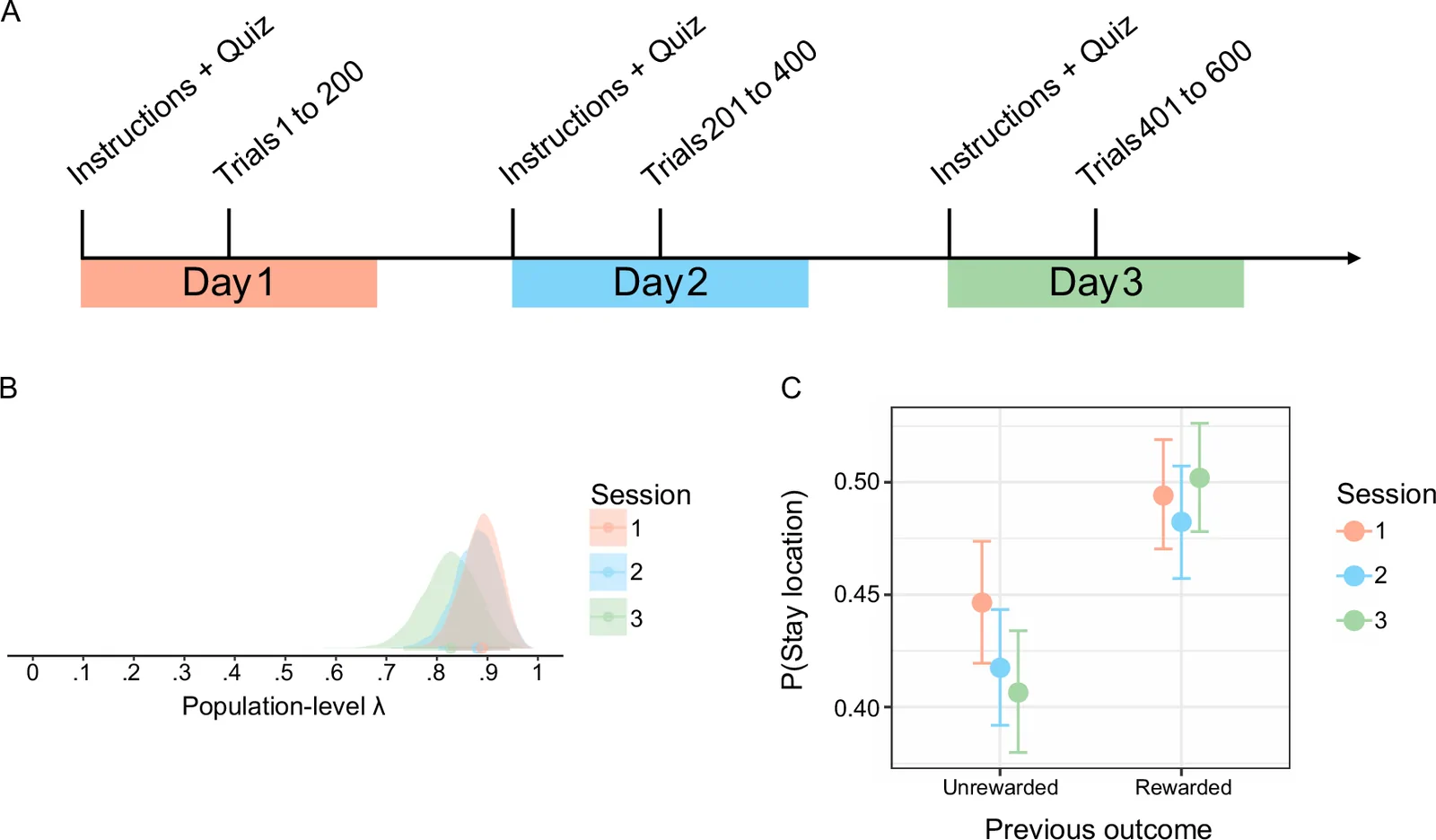
Outcome-irrelevant learning persists with prolonged training and experience. In Experiment 1, participants exhibited outcome-irrelevant learning (i.e., assigning credit to spatial locations) even when task instructions clearly highlighted that locations are randomly allocated. **A** To test whether this tendency diminishes with extended experience, in Experiment 2, we had participants (*N* = 158) complete the story-based version of the task over three consecutive days, with 200 trials per session. At the beginning of each session, participants completed comprehensive instructions and a quiz, ensuring they understood the structure of the task. Colors represent different sessions. **B** Our pre-registered hypothesis was that outcome-irrelevant learning would not decline with practice. Indeed, population-level λ estimates slightly decreased over time, indicating greater (rather than lower) reliance on outcome-irrelevant location values across sessions. Colored circles represent the posterior median, and the horizontal lines represent the 95% CI. **C** A model-agnostic analysis using hierarchical Bayesian logistic regression further supported this finding. We found that the influence of the previous trial’s outcome on current location choice (in trials featuring different options) persisted over time and even increased in the third compared to the first session. Colored circles represent the posterior median, and the error bars represent the 95% CI.

The results of Experiment 1 and the reanalysis of Feher da Silva et al. (2023) suggested that verbal instructions alone are insufficient to prevent individuals from mistakenly attributing credit to outcome-irrelevant representations. Here, we consider the possibility that humans may require prolonged first-hand experience, indicating that locations are indeed outcome-irrelevant. This would align with findings showing that instructions have only a limited effect on human choices^9,44,78^, rendering experience necessary for accurate credit assignment. Thus, in Experiment 2, we examined whether outcome-irrelevant learning decreases with time and experience in the task. We repeated the same task that was used in the previous experiment (Story-based version; Fig. 1A), but this time, newly recruited participants (*N* = 158) repeated it across three consecutive days with 200 trials in each session (600 trials in total; see Fig. 4A). We fitted the same computational model as in Experiment 1, allowing all parameters to vary across sessions within a Bayesian hierarchical model structure. We examined population-level λ values separately for each of the three sessions. First, we replicated Experiment 1 findings showing robust evidence of outcome-irrelevant learning in the first session, despite the use of story-like instructions (λ for Session 1: posterior median = .89, HDI_95%_ [.81, .95]). Furthermore, λ values demonstrated clear evidence of outcome-irrelevant learning in both subsequent sessions (Session 2: posterior λ median = .88, HDI_95%_ [.78, .95]; Session 3: posterior λ median = .83, HDI_95%_ [.71, .92]). Comparing the posterior distributions for the λ values across sessions showed strong evidence that λ values did not increase across time (posterior median λ difference Session 1 vs. 2 = .01, HDI_95%_ [− .10, .13], pd = 56%; posterior median λ difference Session 1 vs. 3 = .06, HDI_95%_ [− .06, .20], pd = 83%; see Fig. 4B), thus implicating that outcome-irrelevant learning tendencies do not diminish over time, even with extended task experience, and lead to reduced performance (Pearson’s r between accuracy and average λ posterior median = .65, HDI_95%_ [.53, .77]; see Fig. 1H).

A similar pattern emerged in the model-agnostic signature. Specifically, for each session, we evaluated the effect of the previous trial’s outcome (Rewarded vs. Unrewarded on trial *n-1*) on the likelihood of repeating the same location choice on trial *n* (Stay vs. Switch), when two different colors were offered than in the previous trial. The model-agnostic signature did not decrease, but rather increased over time (Session 1: posterior median = 0.19, HDI_95%_ [0.04, 0.34], pd = 99.4%; Session 2: posterior median = 0.26, HDI_95%_ [0.11, 0.41], pd ~= 100%; Session 3: posterior median = 0.39, HDI_95%_ [0.23, 0.53], pd ~= 100%; Session 1 vs. 2: posterior median difference = -0.07, HDI_95%_ [− 0.24, 0.09], pd = 80%; Session 1 vs. 3: posterior median difference = − 0.19, HDI_95%_ [− 0.36, − 0.03], pd = 99%; see Fig. 4C). This shows that the outcome-irrelevant model-agnostic signature does not diminish but, if anything, tends to increase over time despite leading to impaired performance.

### Outcome-irrelevant learning extends beyond spatial-motor representations

**Fig. 5:**
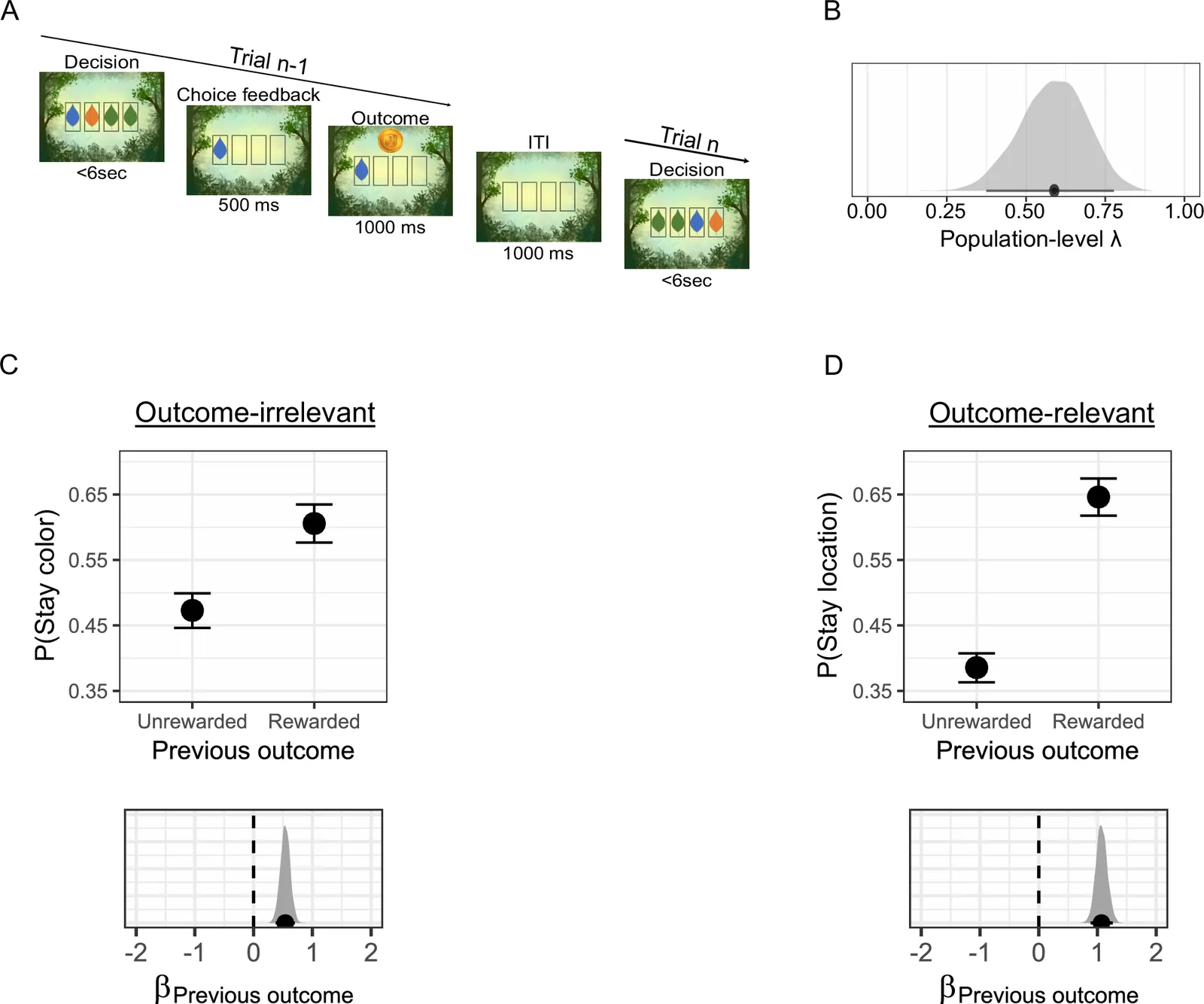
Outcome-irrelevant learning extends beyond spatial-motor representations. **A** To test whether outcome-irrelevant learning reflects a domain-general process or is restricted to spatial-motor representations, we modified the “story” condition from Experiment 1. Specifically, we flipped the outcome-relevance of the two action features, such that the color was now outcome-irrelevant, and the location/response-key was outcome-relevant. On each trial, two out of four locations were offered for choice. The two offered locations in a given trial were marked by two “colored leaves” (e.g., orange or blue), while the two locations that were not available for a given trial were marked by two “green leaves”. Participants (*N* = 195) were comprehensively instructed and tested for their understanding that the specific color of the non-green leaf was irrelevant for outcome prediction, and choices should solely depend on the chosen location. **B** We fitted the computational model to participants’ behavior in Experiment 3, where colors were outcome-irrelevant, and choices should have been determined solely by the location. Higher values of λ indicate greater reliance on outcome-relevant information (in this case, locations). We found a clear indication that participants were unable to inhibit assigning credit to outcome-irrelevant colors, as seen by the posterior distribution of the λ parameter. The black circle represents the posterior median, and the horizontal line indicates the 95% CI. **C** We evaluate whether the previous outcome affects color selection in trial *n*, when the locations from trial *n-1* were not reoffered. We found a robust effect indicating that outcome-irrelevant learning generalizes beyond spatial-motor representations. The lower part of the panel shows the posterior distribution for the effect of the previous outcome. The black circle represents the median, and the upper and lower bars represent the 95% CI. The horizontal line indicates the 95% CI. **D** To further confirm that individuals were able to follow the values of outcome-relevant locations, we estimated a win-stay lose-shift signature on trials where the previously chosen location was reoffered. We found a clear effect of previous outcome on location choice. The lower part of the panel shows the posterior distribution for the effect of the previous outcome. The black circle represents the median, and the upper and lower bars represent the 95% CI. The horizontal line indicates the 95% CI.

To test whether outcome-irrelevant learning is specific to spatial-motor representations, we conducted a preregistered experiment (*N* = 195) using a modified version of the “story” framing from Experiments 1 & 2. Here, the locations were made outcome-relevant while stimulus color was irrelevant to outcome prediction. The task included four available locations (left, middle-left, middle-right, and right). In each trial, participants were offered two of the four locations for their selection. The locations that were offered for selection in a given trial were marked by two “colored leaves” (e.g., orange or blue), while the two locations that were not available for a given trial were marked by two “green leaves” (see Fig. 5A). In each trial, the allocation of the four colors (e.g., green, green, orange, blue) to each of the four locations was randomly assigned by the computer. Therefore, participants had to attend to the color during the selection phase to know which locations were offered. Yet, colors had no association with outcomes, which were determined only by the chosen location. After choosing a location, participants observed whether they obtained a reward or not. Each location led to a monetary outcome (unrewarded or rewarded) with a latent expected probability that was unknown to participants and that slowly drifted across trials. Participants were therefore asked to observe the outcomes that followed their selection and try to make decisions that would maximize return. We made sure, using story-based instructions and a dedicated quiz, that participants understood only the location, and not the color of the stimuli, was outcome-relevant (see Supplementary Note 12).

Confirming our preregistered hypothesis, we found a clear indication that participants assigned credit for the outcome-irrelevant colors. Computational modeling showed that the population-level λ parameter was substantially lower than 1 (posterior median = .59, HDI_95%_ [.38, .78]; see Fig. 5B). Our model-agnostic signature further examined the tendency to re-select a previously selected color as a function of reward. This was examined only in trials where the two previously offered locations were not reoffered for selection in the following trial (see Fig. 5A). We fitted a hierarchical logistic Bayesian regression and found that participants were more likely to repeat a previously chosen color following a rewarded (61%) vs. an unrewarded (47%) trial (posterior median = 0.54, HDI_95%_ [0.38, 0.7], pd ~= 100%; see Fig. 5C). We further estimated the degree to which participants were able to associate rewards with the outcome-relevant locations and found a clear outcome-relevant reward learning signature, such that when the previously chosen location was reoffered, participants were more likely to repeat it following a rewarded vs. an unrewarded trial (posterior median = 1.07, HDI_95%_ [0.88, 1.26], pd ~= 100%; see Fig. 5D). This indicates that despite a clear understanding of the task, individuals found it difficult to inhibit assigning credit to task-relevant, yet outcome-irrelevant colors. Thus, we conclude that outcome-irrelevant learning generalizes to other non-spatial-motor domains.

### Outcome-irrelevant learning cannot be explained by a Pavlovian bias

We performed an additional experiment to minimize the possibility that Pavlovian processes were a main contribution to the previous results^79–81^. Specifically, in Experiments 1–3, the chosen option remained on the screen during outcome presentation. Thus, a Pavlovian association between reward and the outcome-irrelevant chosen side or chosen color could have been formed. Following a Pavlovian to Instrumental transfer^82,83^, this may then lead to a bias towards approaching an outcome-irrelevant feature (i.e., location/color) following a rewarded trial and avoiding it following an unrewarded trial.

**Fig. 6:**
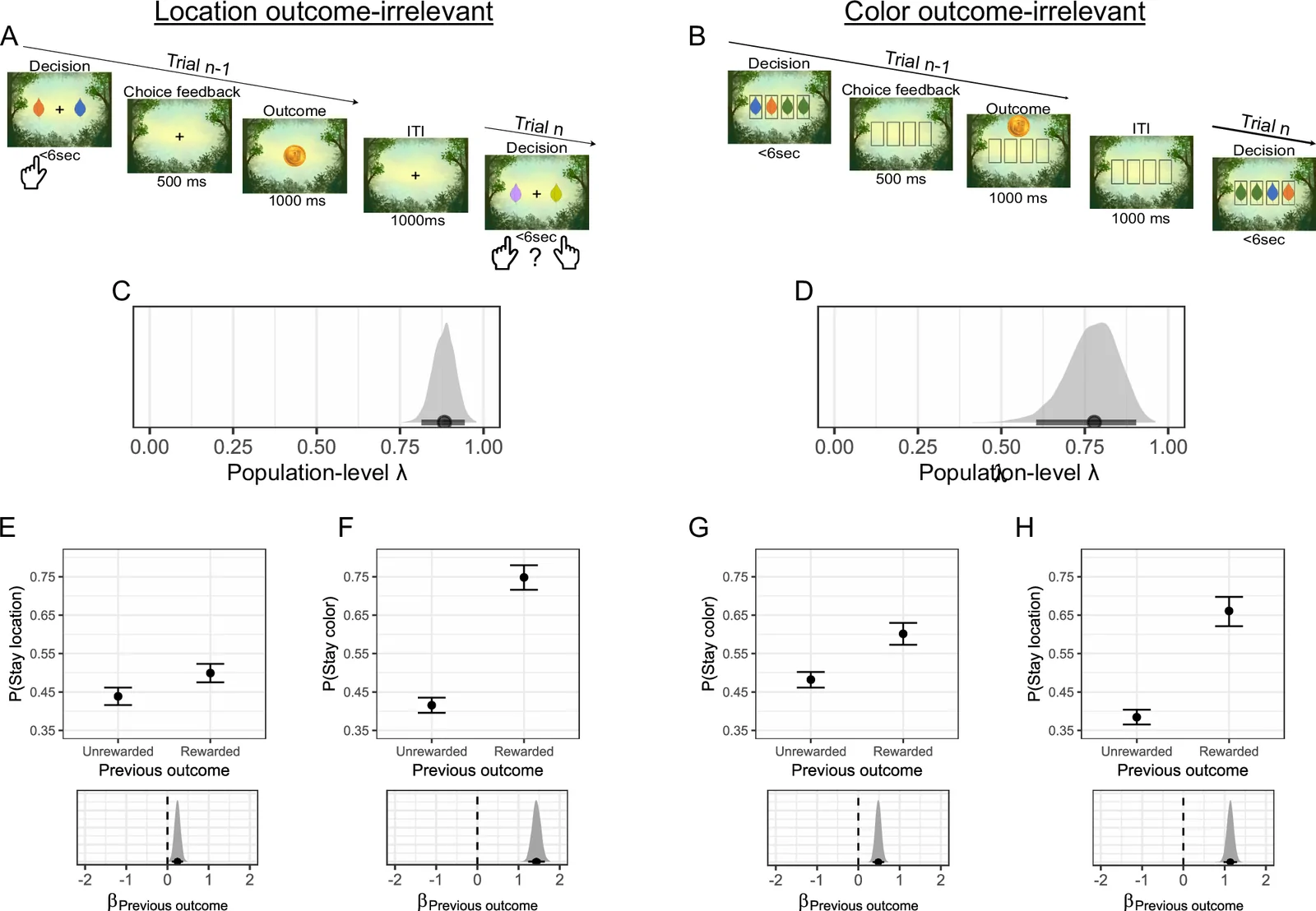
Outcome-irrelevant learning is observed even when choice feedback is omitted. We conducted two additional experiments demonstrating that outcome-irrelevant learning cannot be explained by choice-free Pavlovian association. **A**, **B Trial sequence for Experiments 4A and 4B**. Here, we repeated the tasks used in previous experiments where either location (Exp. 4A; *N* = 129) or color (Exp. 4B; *N* = 190) was outcome-irrelevant. Critically, we omitted the choice feedback presentation completely. This ensured that all features of the two options gained the same exposure on screen, preventing any possible Pavlovian association between the outcome and an outcome-irrelevant feature of the chosen option. **C**,** D Posterior distributions of the λ parameter** estimated separately for each experiment using computational modeling showed a clear indication that even when the two options gained equal presentation on screen, participants were unable to inhibit credit assignment to outcome-irrelevant locations (Exp. 4A) or colors (Exp. 4B). The black circle represents the median, and the horizontal line indicates the 95% CI. **E**,** G** **Outcome-irrelevant model-agnostic signature** showing that participants were more likely to stay with the same outcome-irrelevant feature (i.e., location for Exp. 4A; color for Exp. 4B) after a rewarded trial compared to an unrewarded trial. The lower part of the panel shows the posterior distribution for the effect of the previous outcome. The black circle represents the median, and the upper and lower bars represent the 95% CI. **F**,** H** **Outcome-relevant model-agnostic signature**. We further show the outcome-relevant win-stay lose-shift signature, indicating that participants were clearly able to learn the value of the outcome-relevant feature (colors Exp. 4A; locations Exp. 4B). The lower part of the panel shows the posterior distribution for the effect of the previous outcome. The black circle represents the median, and the upper and lower bars represent the 95% CI.

To test whether Pavlovian associations that might have formed during outcome presentation are the main driver of our effect, we completed two additional preregistered experiments that dramatically reduce the potential contributions of Pavlovian processes. Experiment 4A (*N* = 129) included locations/response keys as outcome-irrelevant and color as outcome-relevant (like Experiments 1&2). Experiment 4B (*N* = 190) included the opposite, namely, color as outcome-irrelevant and locations/response keys as predicting the outcome (like Experiment 3). Critically, in both experiments, we modified the task design so that during both choice feedback and outcome presentation, the chosen location or color was not shown on the screen (see Fig. 6A, B). This minimized potential Pavlovian associations between the outcome-irrelevant features and the outcome.

We replicated our results from previous experiments in both domains, showing a reward-dependent change in the tendency to choose a knowingly outcome-irrelevant feature. We estimated the population-level λ parameter using computational modeling in both experiments and found the λ parameter to be substantially lower than 1 (Experiment 4A: posterior median = .88, HDI_95%_ [.82, .94]; see Fig. 6C; Experiment 4B: posterior median = .78, HDI_95%_ [.62, .92], see Fig. 6D). To corroborate this conclusion, we estimated our model-agnostic signature in Experiment 4A, showing that outcome-irrelevant locations are more likely to be repeated following a rewarded (50%) vs. an unrewarded trial (44%; Experiment 4A: posterior median = 0.24, HDI_95%_ [0.11, 0.38], pd ~= 100%; see Fig. 6E). To further ensure that our manipulation did not interfere with participants’ ability to learn the relevant dimension, we also share results indicating a strong win-stay lose-shift signature for the previously chosen color, such that when a previously chosen color was reoffered, participants were more likely to choose it after a rewarded trial (75%), than after an unrewarded trial (42%; Experiment 4A: posterior median = 1.43, HDI_95%_ [1.23, 1.63], pd ~ = 100%; see Fig. 6F). Similarly, when visual information was outcome-irrelevant (Experiment 4B; see Fig. 6B), we found that participants were more likely to repeat a previously chosen color following a rewarded (60%) than after an unrewarded trial (48%; Experiment 4B: posterior median = 0.48, HDI_95%_ [0.35, 0.62], pd ~= 100%; see Fig. 6G). Participants were again still able to learn the values of the outcome-relevant spatial-motor feature (Experiment 4B: posterior median = 1.14, HDI_95%_ [0.97, 1.3], pd ~= 100%; see Fig. 6H). Thus, we conclude that outcome-irrelevant learning persists even when mitigating the contribution of Pavlovian associations.

### Working memory abilities are associated with automatic credit assignment

**Fig. 7:**
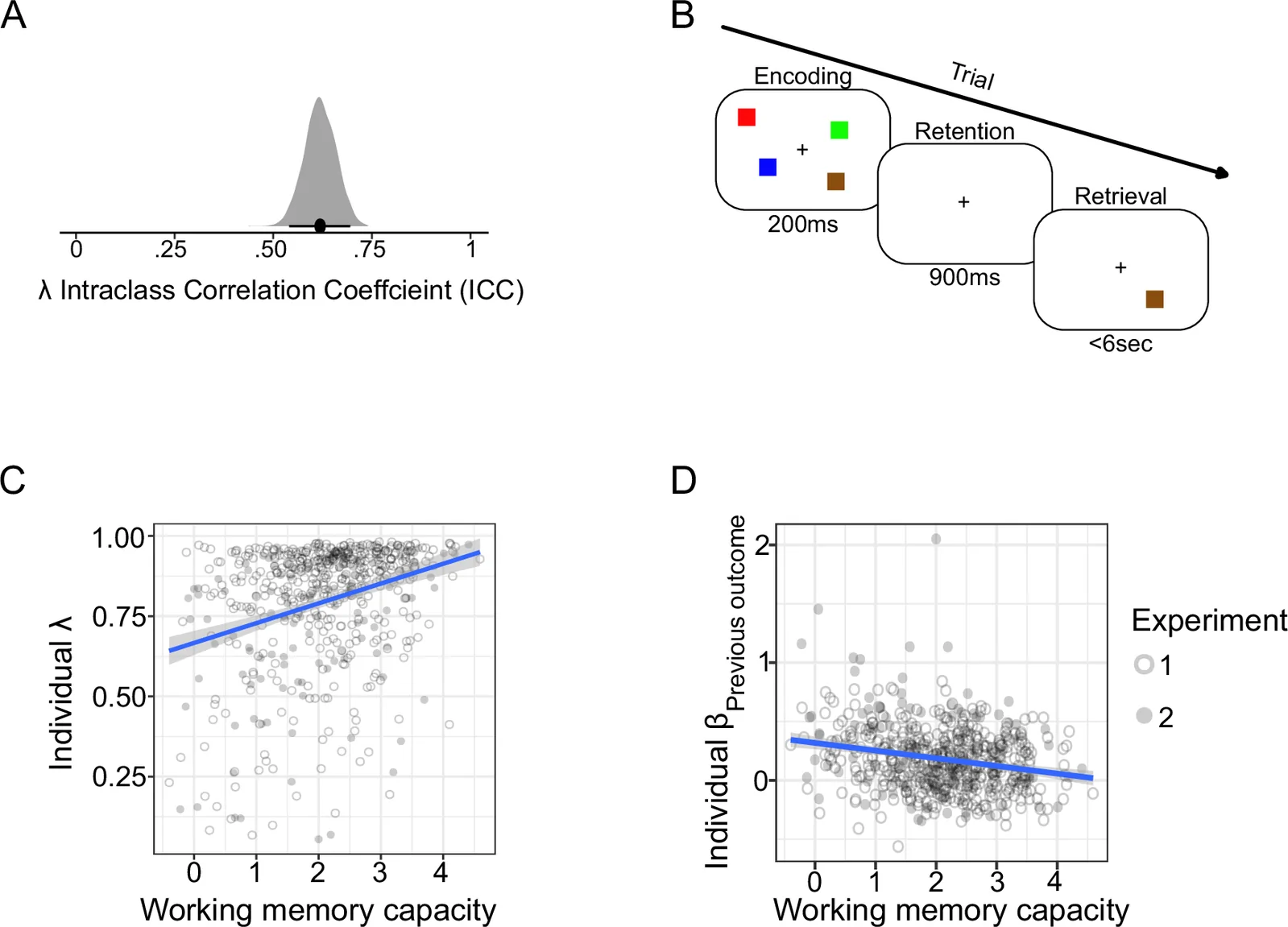
Outcome-irrelevant learning is linked to working memory capacity. Before evaluating individual differences in the λ parameter, we sought to evaluate their reliability across time. **A** For this purpose, we fitted a hierarchical Bayesian linear regression (random intercepts model), quantifying the proportion of total variance attributable to between-subject differences (Exp. 2: *N* = 158), relative to total variance (between- plus within-subject variability). We found λ to be moderately reliable across sessions, with the median posterior ICC being .62 (HDI_95%_ [.54, .70]). The black circle marks the posterior median, and the horizontal line represents the 95% CI. **B** Working memory was assessed in Experiments 1 and 2 using a classical change-detection task completed on a separate session from the reinforcement learning task^66,90,91^. Participants completed 120 trials, each comprising three stages: encoding, retention, and retrieval (see “Methods”). During encoding, participants viewed an array of 4 or 8 colored squares and were instructed to remember them. After a brief retention interval, a single target square appeared, and participants reported whether its color matched the square previously shown at the same location. **C** Across both experiments, we observed a robust positive correlation between participants’ λ estimates and their working memory capacity, such that individuals with higher λ values tended to show stronger working memory performance. Empty dots represent data from Experiment 1 (*N* = 453), filled dots represent data from Experiment 2 (*N* = 135), and the regression line is depicted in blue with the shaded band representing the 95% CI. **D** Consistently, we found that participants with higher working memory capacity exhibited weaker outcome-irrelevant learning, as indexed by their individual model-agnostic regression coefficients. Empty dots represent data from Experiment 1 (*N* = 453), filled dots represent data from Experiment 2 (*N* = 135), and the regression line is depicted in blue with the shaded band representing the 95% CI.

Finally, we examined whether individual differences in working memory capacity were associated with outcome-irrelevant learning. Given concerns about the reliability of individual-level computational modeling parameters, we first sought to establish the reliability of λ^84–86^. For this purpose, we leveraged the repeated-measures design of Experiment 2 to assess the reliability of individual-level λ estimates across the three sessions. Specifically, we fitted a hierarchical random-intercept model, allowing us to compute the Intraclass Correlation Coefficient (ICC) by comparing within- and between-subject variance^87–89^. The resulting ICC was 0.62 (HDI_95%_ [.54, .70]), indicating that λ was moderately reliable across sessions (see Fig. 7A; note that the ICC estimate may be over-conservative, as our computational model treated sessions as a fixed effect rather than modeling them hierarchically within individuals; see ref. ^85^).

Next, to test our prediction that outcome-irrelevant learning would be higher in individuals with lower working memory abilities, we used data from Experiments 1 and 2, where we measured participants’ working memory capacity using the well-established change detection task and examined its correlation with each participant’s λ (see Fig. 7B)^66,90,91^. Specifically, we fitted a linear regression and found a positive association such that individuals with lower working memory capacity had lower λ values (Experiment 1: *N *= 453 participants who had eligible data in both tasks; posterior median = 0.25, HDI_95%_ [0.16, 0.34]; pd ~= 100%; Experiment 2: *N* = 135 (λ was averaged over sessions), posterior median = 0.38, HDI_95%_ [0.24, 0.52]; pd = ~ 100%; see Fig. 7C). We further examined the relationship between our model-agnostic signature and participants’ working memory abilities across both experiments and found a consistent negative association (Experiment 1: *N* = 453; posterior median = − 0.17, HDI_95%_ [− 0.26, − 0.08]; pd ~= 100%; Experiment 2: *N* = 135, posterior median = − 0.33, HDI_95%_ [−0.47, −0.18]; pd ~= 100%; see Fig. 7D). These results suggest that outcome-irrelevant learning is more pronounced in individuals with lower working memory capacity.

### Inattentive responding does not lead to outcome-irrelevant learning

Across our work, we reported a reward-driven stay/switch behavioral effect in the outcome-irrelevant dimension, which is tightly associated with the λ parameter (Fig. 2). We thus interpreted this behavioral effect as a direct, model-agnostic signature of outcome-irrelevant learning. Here, we further aimed to show that this signature effect cannot arise from random responding due to task inattention.

**Fig. 8:**
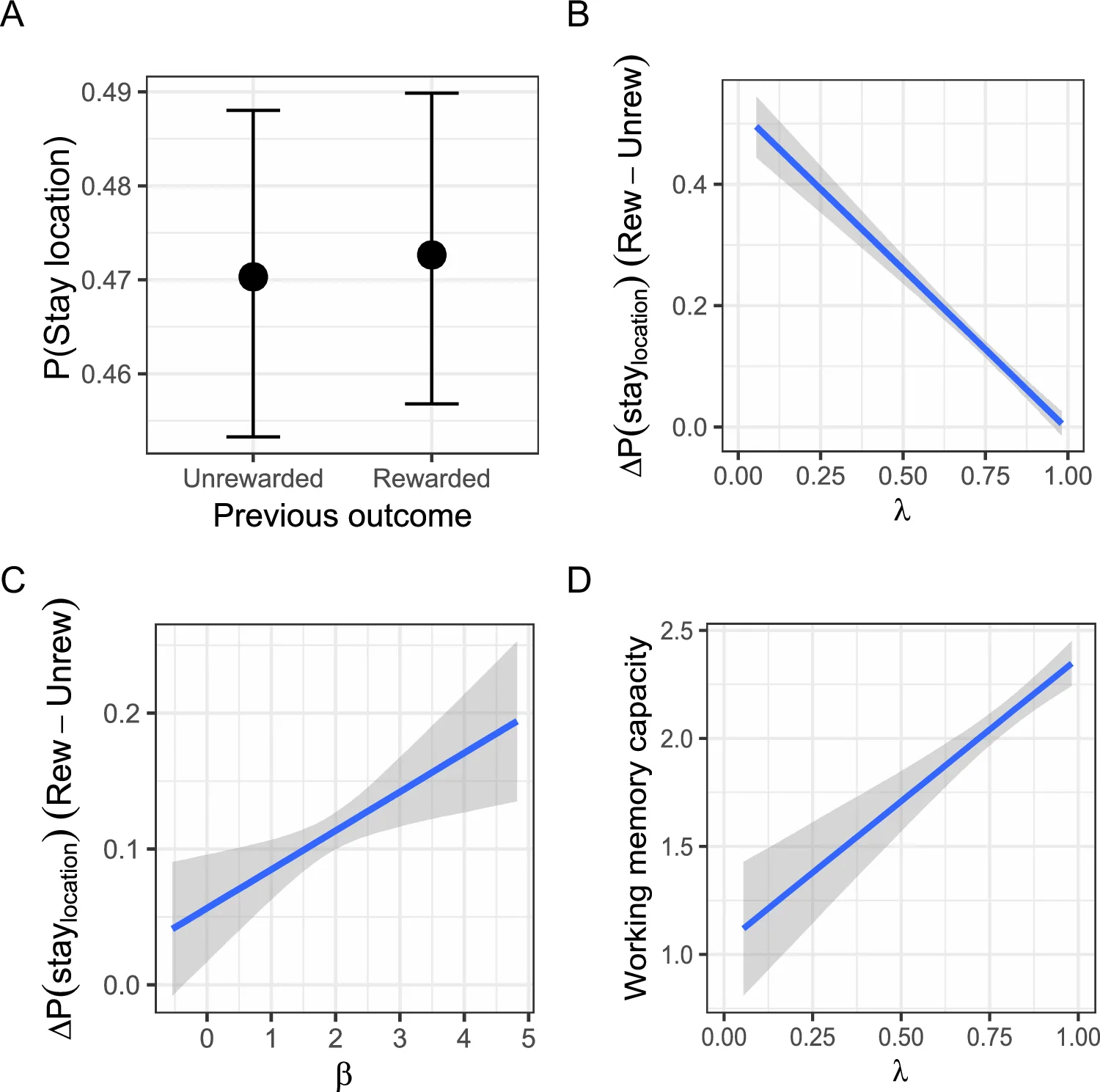
Inattentive responding does not lead to outcome-irrelevant learning. **A** To ensure that the reward-driven stay/switch behavioral effect in the outcome-irrelevant dimension is not driven by random responding, we simulated agents (*N* = 200) with empirically estimated parameters but with no influence of outcome-irrelevant learning on decision (i.e., *λ* = 1). To explore the possibility that outcome-relevant learning combined with inattentiveness gives rise to outcome-irrelevant learning, on one third of the trials, we included a “lapse of attention”, where β is temporarily set to 0. We found that these agents clearly do not show the model-agnostic effect. The black circle represents the posterior median, and the error bars represent the 95% CI. **B**,** C** Next, we used data from Experiments 1 & 2 (Exp. 1: *N* = 504; Exp. 2: *N* = 158) and predicted for each individual the model-agnostic signature effect based on both λ and β parameters. Despite λ and β being positively correlated among individuals in our sample, the model-agnostic signature is differentially associated with these parameters. While higher degrees of λ are associated with a smaller outcome-irrelevant learning signature, higher degrees of β are associated with a greater outcome-irrelevant learning signature. This is to be expected, given that random responding can not generate the effect. The blue lines represent the regression lines, with the shaded areas representing the 95% CI. **D** Finally, we show that the association between λ and working memory is robust even when controlling for both β and general choice accuracy in the bandit task. That is, the association is not due to inattentive responding in both tasks (Exp. 1: *N* = 453; Exp. 2: *N* = 135). The blue line represents the regression line, with the shaded area representing the 95% CI.

We addressed this question using three independent analyses. First, we simulated 200 agents using empirically derived parameter values (i.e., learning rate, color-perseveration, and location-switching tendencies; see Supplementary Table S1). We, however, eliminated outcome-irrelevant learning in those agents by fixing λ to 1. We further introduced attentional lapses in one third of the trials for all agents by temporarily setting β = 0, resulting in value-independent choices on those trials. Nevertheless, we were able to recover λ as equal to 1 (see Supplementary Note 7). Importantly, an in-silico analysis revealed no evidence for a reward-driven stay/switch behavioral effect in the outcome-irrelevant dimension (see Fig. 8A). This demonstrates that arbitrary random responding alone cannot produce the empirical behavioral signature we associate with outcome-irrelevant learning.

Second, using data from Experiments 1 & 2, we fitted a multiple linear regression predicting each individual’s reward-driven stay/switch behavioral effect in the outcome-irrelevant dimension using both their λ and β estimates. λ values were found to be negatively associated with the model-agnostic signature, even when controlling for individual differences in the β parameter (posterior median = − 0.53, HDI_95%_ [− 0.60, − 0.45], pd ~= 100%; see Fig. 8B; see Supplementary Note 8 for illustration that this association holds even amongst individuals with high β). In contrast, we found that a one-unit reduction in β is associated with a 3% decrease in the aforementioned effect (posterior median = 3%, HDI_95%_ [1%, 5%], pd ~= 100%; see Fig. 8C). Therefore, if anything, random responding (i.e., low β) is associated with a lower signature effect in the outcome-irrelevant dimension. This asserts that the reward-driven stay/switch behavioral effect in the outcome-irrelevant dimension reflects a behavior that cannot arise from random responding.

Finally, to further illustrate our point that random responding cannot give rise to the aforementioned association with working memory, we fitted a linear multiple regression analysis predicting working memory capacity by λ while controlling for inattentive/random responding. We found that even when controlling for β and the general choice accuracy in the bandit task, there is a clear association between working memory capacity and the λ parameter (Data from Experiments 1 and 2; posterior median = 1.32, HDI_95%_ [0.92, 1.73], pd ~= 100%; see Fig. 8D).

## Discussion

Our findings add to a growing body of consistent evidence regarding credit assignment processes to outcome-irrelevant action features in human reinforcement learning^13,14,16–20^. In the present study, both computational modeling and complementary regression analyses revealed that participants engaged in such learning and assigned value to action features that did not predict reward, despite this leading to impaired performance and reduced monetary gains. Outcome-irrelevant learning persisted despite explicit instruction (Experiment 1) and extensive experience (Experiment 2). Furthermore, this tendency was found to be reliable across sessions and stronger in participants with lower working memory resources. We further found that outcome-irrelevant learning is not restricted to spatial-motor features (Experiment 3), and is driven by instrumental learning rather than merely by a Pavlovian association (Experiment 4). We now turn to interpreting the theoretical and cognitive implications of these findings.

The reinforcement learning literature has argued for a dissociation between elaborative model-based processes, which rely on an internal representation of the environment, and model-free processes in which learning is based on action-outcome history alone^92–96^. Given that participants were provided with elaborate instructions and quizzed to make sure they had understood these instructions, it seems unlikely that outcome-irrelevant learning is the result of a pervasive use of wrong cognitive models. However, we cannot rule out that at least some participants might have formed wrong cognitive models of the task, making it challenging to confidently affirm that outcome-irrelevant learning is purely model-free^45,46^. Furthermore, recent work has questioned the existence of such perfect dissociation between model-free and model-based processes^67,68,97^. Thus, future work should carefully address whether outcome-irrelevant learning is model-free by definition or can also reflect residual model-based tendencies.

### Can we consider outcome-irrelevant learning to be an automatic process?

Automatic processes are defined as cognitive operations triggered by irrelevant information that interfere with goal-directed behavior^29–32^. Our results align with this definition, as spatial-motor action representations (stimulus locations and the response keys used to report selections in Experiments 1, 2, and 4A) and visual representations (colors in Experiments 3 and 4B) were not predictive of rewards by design. These action features are therefore outcome-irrelevant, and associating them with observed outcomes is counterproductive, as it injects noise into value estimates and thus reduces accuracy and monetary gains. Accordingly, the formation of such non-veridical or spurious associations can be interpreted as evidence of an automatic credit assignment process. Here, we define automatic credit assignment as the spontaneous attribution of reward value to temporally contiguous action features that are causally unrelated to the outcome and known with high certainty to be incidental. A few considerations should be addressed in detail to clarify the scope of this process and the specific meaning of automaticity in this context.

A first major concern when interpreting credit assignment to outcome-irrelevant action features as automatic is that we might misinterpret this behavior as automatic when it actually reflects an explicit strategy. For instance, participants who are uncertain about the instructions and the role of the bandits’ locations might deliberately explore spatial-motor features under the assumption that these could influence rewards. Previous studies have underscored this issue, claiming that outcome-irrelevant learning can, under certain conditions, reflect a “model-based confusion about the relevance of different task features” (p. 14^45^). In Experiment 1, we followed the pivotal study by Feher da Silva et al. (2023), which set a benchmark for delivering clear, bona-fide instructions in reinforcement-learning experiments. To meet this standard, we provided participants with an intuitive narrative that detailed the task structure and provided a rationale for the random changes in outcome-irrelevant features (“wind randomly shuffling the locations of leaves falling from a tree”). Participants’ understanding of the task design was assessed with a comprehension quiz that confirmed they could identify the random, outcome-irrelevant task features before testing began. To evaluate the generality of our claims, we also reanalyzed the data of Feher da Silva et al. (2023), who used a comparable narrative in a more complex two-stage decision task. The results of Experiment 1, together with the reanalysis of Feher da Silva et al.’s (2023) dataset, were conclusive. Even when holding accurate knowledge of the task structure, participants still assigned credit to outcome-irrelevant action features.

A second key concern in arguing for automatic credit assignment is that, even after being clearly informed which action features were not predictive of reward, participants might still have uncertainty about the outcome-relevance of these features (for instance, because they mistrust the instructions). Such uncertainty could prompt them to deliberately assign value to outcome-irrelevant features and, through trial and error, test whether these features predict rewards. Previous studies have shown that, under conditions of state uncertainty, humans are adept at inferring through trial and error which action features are reward-predictive and which can be ignored^3,4,12,98^. In Experiment 2, participants completed extended testing that gave them ample opportunity to learn through experience, which action features were uninformative of reward. But even after three sessions comprising 600 trials in the same environment, and despite outcome-irrelevant learning leading to reduced monetary gains, participants continued to assign credit to outcome-irrelevant features. This aligns with a broader framework where, at a lower representational level, the reward learning system assigns value to task-relevant yet outcome-irrelevant features, as demonstrated across experiments. At a higher, strategy level, a fully flexible system should learn that relying on these features is counterproductive and reduce their influence with practice. Experiment 2 offered a substantial opportunity for this strategic adjustment, yet no reduction in outcome-irrelevant learning was observed. This indicates that higher-level policy learning does not override feature-level credit assignment, which continues to operate even when it is detrimental to performance.

Based on evidence from Experiments 1 and 2, we conclude that outcome-irrelevant learning exhibits the defining characteristics of automatic cognitive processing, persistently acting based on irrelevant information in a counterproductive manner. However, research in the selective-attention literature indicates that automaticity is best conceived as a continuum, with some processes being more automatic than others^29,31,33^. The degree of automaticity depends on the extent to which a process meets additional criteria, such as occurring involuntarily and without conscious monitoring^30,31,99–102^. Automatic processes are also viewed as effortless and capable of running in parallel without drawing on executive resources^30–33,99,101,103^. Determining whether credit assignment meets these stricter criteria is challenging. Self-reports gathered after a task often fail to capture the effort invested or the degree of conscious monitoring, especially when the underlying cognitive processes are subtle^104,105^. Further rigorous behavioral and neurophysiological measures are therefore required to establish how much effort and awareness are involved in outcome-irrelevant credit assignment processes.

### Outcome-irrelevant learning extends beyond spatial-motor representations

Previous work suggested that interference from outcome-irrelevant features might be limited to spatial-motor representations^17,54^. However, in Rmus et al., the outcome-irrelevant visual feature was also irrelevant for executing the task. That is, participants could have made their choice without exerting any attention to the visual feature (color). Thus, in Rmus et al., the lack of interference from the visual feature may be due to it being irrelevant to the whole task, not just the outcome. Here, we designed a task where visual features are necessarily attended during action selection, yet are irrelevant for outcome prediction. We found clear evidence that value is assigned to outcome-irrelevant colors. We conclude that credit may be automatically assigned to all action features that are part of the task set, even when these are outcome-irrelevant.

### Outcome-irrelevant learning is driven by credit assignment to action features

Outcome-irrelevant learning can, in principle, be interpreted through a Pavlovian-to-Instrumental Transfer (PIT) account^82,83^. In PIT, a Pavlovian cue that is associated with reward can later invigorate an instrumental response directed toward that cue. According to this Pavlovian account, the association between a location and a reward is formed independently of one’s action. In this view, when an option is chosen, then the outcome-irrelevant feature of the selected option might become linked to reward simply because the reward co-appears with that feature on screen, rather than having credit being assigned to the individual’s choice. Experiments 1-3 may have been susceptible to this explanation, as the outcome-irrelevant features (location in Experiments 1-2, color in Experiment 3) were present on the screen while the outcome was displayed. This temporal overlap may have led the irrelevant feature to acquire a Pavlovian association, which could then bias instrumental responding toward the same location or color in subsequent trials. To address this concern, we designed Experiment 4 so that the outcome-irrelevant features (4A-locations/4B-colors) were displayed symmetrically during choice feedback and outcome presentation, eliminating any differential pairing between reward and an irrelevant feature. We still observed robust outcome-irrelevant learning effects, suggesting PIT is not the sole mechanism driving the outcome-irrelevant learning effect, and that choice-based credit assignment to outcome-irrelevant features persists in shaping behavior.

### Cognitive control can modulate and regulate automatic credit assignment

The automatic assignment of credit to outcome-irrelevant action features can be understood through the Theory of Event Coding^106,107^. This framework holds that perceptual and motor elements of an action are automatically bound into an “event file,” a unified episodic representation that can be rapidly retrieved when similar situations arise^108^. However, if an event file contains features that later become irrelevant (e.g., through experimental manipulation), the retrieval of the event file can impair performance^109,110^. When an action immediately produces an affective outcome, the event file also stores stimulus-response-effect bindings^108,111^. Accordingly, in a multi-armed bandit trial, the stimulus color, spatial location, response key, and reward are encoded altogether. Thus, the later appearance of another stimulus in the same location can reinstate this event file and bias choice despite the irrelevance of location and motor response^108,112^. Hence, outcome-irrelevant learning arises naturally from event-coding principles that assert an automatic binding of perceptual, motor, and effect features into a single episodic memory.

The fact that a cognitive process is automatic does not mean it is exempt from regulation and control^29,31,33,103^. Within the event coding framework, control mechanisms can modulate both the binding and retrieval of event files, so that features not represented in the participant’s goals are less likely to be integrated and exert a weaker influence when the event file is retrieved^108,113,114^. The selective-attention literature argues that working memory capacity is a key resource for regulating automatic processes according to goal relevance because it supports the maintenance of task instructions, thereby restricting the influence of irrelevant features throughout the task^56,115,116^. Consistent with this view, individual differences in working-memory capacity predict the degree of interference produced by automatic event bindings^117^. Working memory thus plays a critical role in keeping goal-relevant information accessible and shielding behavior from being influenced by irrelevant associations^62,63,118,119^.

In reinforcement learning, working memory is essential for tracking which action features are causally predictive of reward while disregarding those that are not^55^. In our tasks, spatial features (in Experiments 1,2, and 4A) and visual features (in Experiments 3 and 4B) are required to convey a choice (i.e., task-relevant) yet are unrelated to reward (i.e., outcome-irrelevant). Thus, participants must attend to the outcome-irrelevant location/color to select a response and then immediately disregard that same feature as non-predictive during credit assignment. It could be that maintaining and updating this representation taxes working memory, making individuals with lower capacity more prone to misattributing value to task-relevant yet outcome-irrelevant features. Consistent with this account, we observed a strong association between outcome-irrelevant learning and working-memory capacity, with larger outcome-irrelevant learning effects in participants who scored lower on a working-memory measure^13^. Further studies that orthogonally manipulate task-relevance and outcome-relevance could delineate the conditions under which automatic credit assignment is more adequately regulated.

### Potential alternative explanations for outcome-irrelevant learning

An alternative account for our findings is the “value of control” theory, which posits that exerting cognitive control carries a subjective cost and is engaged only when its expected benefits outweigh that cost^120–124^. Within this framework, individuals constantly face meta-control decisions, evaluating whether the potential reward gain justifies the effort required to override habitual or automatic responses^125–127^. Empirical evidence supports this view, with Kool et al. (2016)^128^ showing that people arbitrate between a computationally inexpensive model-free strategy and a more effortful model-based strategy based on expected accuracy and monetary gains^128–130^. From a value-of-control perspective, outcome-irrelevant learning may persist because the modest loss in reward does not justify the effort needed to suppress automatic credit assignment^124,131^. Future research should test this prediction by manipulating how much inhibiting outcome-irrelevant information affects earnings.

Outcome-irrelevant learning may potentially serve as a “tie-breaking” mechanism in decisions made under complete uncertainty, where no reward-relevant information is available to guide choice (see Supplementary Note 4 for a relevant analysis). Such decisions have been described in the literature as “picking” rather than “choosing,” reflecting the absence of a rational basis for preference^132–134^. It has been argued that true picking does not exist, since some internal or external influence always nudges the decision-maker toward one option over another^135^. In this spirit, learning cumulative outcome-irrelevant noise, such as tracking which side of the screen temporarily tends to yield more rewards, could enable individuals to generate arbitrary yet structured decisions, as opposed to truly random ones^136^. Under the assumption that truly random guessing is challenging to execute, individuals can rely on an internal coherent logic^137^. Thus, such a mechanism of “arbitrary exploration” may be similar, yet potentially more realistic, than the “random exploration” that is often described in the literature^138^.

Finally, we suggest that the failure to regulate outcome-irrelevant value learning may reflect a cost-benefit trade-off^130^. For a decision to be made based on outcome-irrelevant values, regulation must fail both during value updating and during action deliberation. Notably, the performance cost in the form of reduced gains emerges only when outcome-irrelevant values are not regulated at the time of choice^15^. In contrast, merely tracking the value of an outcome-irrelevant feature might be beneficial by enabling early detection of changes in a volatile environment. For example, consider someone learning to pick apples from a tree while correctly ignoring leaf shape, which is initially unrelated to taste. If a pathogen later makes leaf shape predictive of taste, having encoded the value of leaves’ shape would allow the learner to rapidly detect this emerging association, something that would not be possible if those values had been entirely ignored. Since real-world scenarios are rarely static or guided by explicit instructions, outcome-irrelevant learning may offer greater flexibility in typical dynamic environments. The regulation failure we document may thus reflect the background operation of a change-detection strategy, which carries the cost of allowing irrelevant values to occasionally contaminate choice^139,140^. The strong association with working memory abilities raises the question of whether individuals with high working memory capacity regulate outcome-irrelevant value updating during learning or, alternatively, allow unregulated values to accumulate, but then suppress them more effectively at the time of choice. Future studies should directly examine this and explore whether inhibiting outcome-irrelevant learning comes at the expense of reduced flexibility in the face of change^141^.

### Limitations

The conclusions of the current study should be interpreted in light of several limitations and outstanding questions. First, stimulus location was perfectly confounded with the keypress required to select it, preventing us from isolating credit given to a certain location in space from credit given to motor commands. Future work should uncouple these factors to pinpoint the features driving outcome-irrelevant learning. Moreover, while we demonstrate that working memory capacity moderates outcome-irrelevant learning, it is yet unknown whether working memory plays a mechanistic role in this process ^142^. One option is that by actively maintaining knowledge about outcome-relevance, working memory resources are essential for such regulation. But given the well-established association between working memory and general intelligence^143^, research should explore whether the association we found reflects broader aspects of intelligence and cognitive control^142,143^. To discern whether the association between working memory and outcome-irrelevant learning represents a causal mechanism, future experiments should carefully manipulate working memory resources to assert that this relationship is causal. Finally, the current design does not allow us to reliably estimate a separate learning rate for each action feature. Thus, future experiments should aim to establish whether the learning rate for outcome-irrelevant dimensions is substantially different from that of outcome-relevant dimensions.

In conclusion, the present study demonstrates that outcome-irrelevant learning is an inherent property of human behavior, persisting despite clear instructions and extensive experience. This appears to result from automatic credit assignment mechanisms that operate independently of explicit task goals. Collectively, these results call for a revision of existing reinforcement learning frameworks to account for the persistence of credit assignment to task-relevant, but outcome-irrelevant action features. We thus encourage the incorporation of outcome-irrelevant learning into human reinforcement learning modeling practices.

## Methods

### Participants

We pre-registered our hypotheses and provided sample size justification using a Bayesian precision analysis^144,145^ (All preregistrations can be accessed using this link: https://github.com/shahar-lab/Automatic-value-learning-results-in-counterproductive-human-behavior/tree/main/Preregistrations). The study protocol for all experiments was approved by the Research Ethics Council of Tel Aviv University (#6707), and all participants signed informed consent before participating in the study. All participants received monetary compensation for their participation. We did not conduct sex or gender analyses, as these variables were not relevant to the specific research question addressed in this study. Data collection and analysis were not performed blind to the conditions of the experiments.

***Experiment 1***: A total of 562 Prolific workers entered the study online in exchange for monetary compensation. All participants were aged 18–50 and reported normal or corrected vision and no current or past psychiatric or neurological diagnosis, color blindness, or brain injury. 279 participants were assigned to the abstract condition (Mean age = 32, SD = 8.4; 159 Male, 119 Female, 1 Other) and 283 participants to the story condition (Mean age = 31.3, SD = 7.9; 147 Male, 122 Female, 14 Prefer not to say). Participants completed the reinforcement learning task in the first session, and in the second session, on the next day, a working memory change-detection task. Of these, 524 participants completed both sessions on consecutive days.

***Experiment 2***: A total of 198 Prolific workers entered the study online in exchange for monetary compensation. All participants were aged 18–50 and reported normal or corrected vision and no current or past psychiatric or neurological diagnosis, color blindness, or brain injury. In Sessions 1–3, participants completed the reinforcement learning task, and in Session 4, a working memory change-detection task. 178 participants completed all four sessions on consecutive days (Mean age = 30.9, SD = 8.2; 93 Male, 85 Female).

***Experiment 3***: A total of 201 Prolific workers entered the study online in exchange for monetary compensation. All participants were aged 18–50 and reported normal or corrected vision and no current or past psychiatric or neurological diagnosis, color blindness, or brain injury. Participants completed a reinforcement learning task (Mean age = 32.5, SD = 8.2; 104 Male, 96 Female, 1 prefer not to say).

***Experiment 4A***: A total of 150 Prolific workers entered the study online in exchange for monetary compensation. All participants were aged 18–50 and reported normal or corrected vision and no current or past psychiatric or neurological diagnosis, color blindness, or brain injury. Participants completed a reinforcement learning task (Mean age = 32.4, SD = 8.2; 94 Male, 55 Female, 1 prefer not to say).

***Experiment 4B***: A total of 201 Prolific workers entered the study online in exchange for monetary compensation. All participants were aged 18–50 and reported normal or corrected vision and no current or past psychiatric or neurological diagnosis, color blindness, or brain injury. Participants completed a reinforcement learning task (Mean age = 32.3, SD = 8.0; 100 Male, 101 Female).

### Authentic respondent verification

We implemented measures to prevent AI bots from completing the task. First, we included a CAPTCHA at the beginning of the survey to block automated responses. Second, we incorporated two open-ended questions during the study: one asked participants to explain their understanding of the instructions, and the other invited them to describe their experience with the task and disclose whether they were an AI.

Details regarding the sample and procedure for the data used by Feher da Silva et al. (2023) can be found at https://www.nature.com/articles/s41562-023-01573-1.

### Reinforcement learning task

Experiment 1

Participants completed a four-armed bandit task in which, on each trial, the computer randomly selected and presented two options for selection. Options were distinguished by unique colors. Each participant completed 50 trials per block across four blocks, with a new set of four colors introduced in each block. The trial began with a fixation mark for 1 sec, followed by the presentation of two options. The two offered options were randomly positioned on either the left or right side of the screen, and participants made their selection using the left (“s”) or right (“k”) response key on a QWERTY keyboard (within a 6-second response window). After selecting an option, the chosen option remained on screen for 0.5 sec without the unchosen option. Next, a feedback screen appeared where the selected option remained on screen, and the monetary outcome (either a gold coin in rewarded trials or a black coin in unrewarded trials) appeared at the screen center for 1 sec (see Fig. 1 for trial sequence). The probability of providing a reward was determined only by the identity of the chosen option. Each option had a probability of providing a reward on each trial based on a slowly varying true expected value (two random-walk sets were counterbalanced between participants, who knew that reward probabilities slowly fluctuate; see Fig. 1C). Each block included 4 new colored options and had the same random-walk set.

Both the abstract and story conditions followed the same task sequence, differing only in the framing of the task and the accompanying instructions. Each version included a mandatory quiz that participants were required to complete with 100% accuracy in order to proceed. The quiz comprised 10 questions assessing participants’ understanding of the task’s overall goal, response keys, and trial sequence. Given the importance of participants’ understanding the random assignment of stimuli to spatial locations, the quiz included a dedicated question addressing this concept. In the abstract condition, this question ensured that participants understood stimuli were randomly assigned to locations by the computer. In the story condition, the equivalent explanation involved stimuli being blown to the left or right by a random wind. Participants who answered this question incorrectly were provided with an additional explanation emphasizing the outcome-irrelevance of the spatial locations (see Supplementary Note 12).

The abstract version included standard framing typically used in previous studies^13,14,146^. The story version was framed as a “*Magical Forest Game*” to provide participants with a narrative that could ease the encoding and maintenance of information about the environment’s model^45,46,147,148^. Participants were told that in an enchanted forest, a *magical tree* with special *colored leaves* exists (see Supplementary Note 12). Each time, two colored leaves will drop from the magical tree and fall below it. Each leaf will sometimes fall to the left and sometimes to the right due to the random direction of the wind (depicted in instructions). Importantly, a *wizard* living in the enchanted forest may be willing to buy the colored leaves for his potion. The wizard has hidden preferences for the leaves that can change throughout the game. We explained to participants that if they select a leaf that the wizard currently prefers, they will be more likely to receive a gold coin for their chosen leaf. Also, we explicitly note to participants that (1) since the wind is blowing at random, the location of the leaves has no meaning to the wizard, who only cares about the color, and (2) since the wizard needs many leaves, the value of one will not indicate the value of the other. Participants were told that they should make selections, observe the selling outcomes, and do their best to make optimal choices to receive as much reward as possible.

Experiment 2

In Experiment 2, the trial sequence was identical to the “story” version from Experiment 1. Each session, participants completed 200 trials, resulting in a total of 600 trials. The 200 trials in each session were divided into four blocks of 50 trials. Each participant was presented with a single set of stimuli (i.e., colored leaves) that remained constant across all 600 trials, enabling generalization of learning across sessions. Four different stimulus sets were created and randomly assigned to participants. In addition, four corresponding sets of random walks were generated to define the expected values for each option throughout the 600 trials. Each participant was assigned one of these sets, supporting continuous learning across testing sessions.

Experiment 3

In Experiment 3, we used the same framing as in the “story” version of Experiment 1, but made the locations outcome-relevant while stimulus color was irrelevant to outcome prediction. The task included four available locations (left, middle-left, middle-right, and right). Each trial began with the presentation of four black rectangles, which participants had been informed corresponded to four potions the forest wizard might want to buy leaves for. Participants were offered two of the four locations for their selection (selections were made by pressing “S” for choosing left, “F” for middle-left, “H” for middle-right, and “K” for right). The two locations available for selection on each trial were indicated by colored potion-ready leaves (with the specific color pair counterbalanced across participants: orange and blue or red and cyan). The two unavailable locations were marked by green leaves, which participants were instructed were “young leaves” that are not potion-ready and thus the wizard never wants to buy (see Fig. 5A). The allocation of the four colors (e.g., green, green, orange, blue) to each of the four locations was randomly assigned by the computer, and participants were instructed that this was randomly determined by the forest wind. Colors had no association with outcomes, which were determined only by the chosen location. After choosing a location, participants observed the chosen option for 0.5 seconds, followed by the presentation of the outcome for 1 second alongside the chosen option. The outcome appeared centered horizontally above the four black rectangles on the screen. Each location led to a monetary outcome (unrewarded or rewarded) with a latent expected probability that was unknown to participants and that slowly drifted across trials (two sets of random walks were counterbalanced between participants). Participants were therefore asked to observe the outcomes that followed their selection and try to make decisions that would maximize return. We made sure, using story-based instructions and a dedicated quiz, that participants were well aware that only the location, and not the visual color of the stimuli, was outcome-relevant. Participants completed 200 trials divided into four blocks of 50. The same locations and colors were used for each participant throughout the entire task, and reward probabilities followed a continuous random walk across all 200 trials.

Experiment 4A

Experiment 4A included a modified version of the trial sequence of the “story” version of Experiment 1. The only difference was that after selecting an option, both options had disappeared from the screen, and a fixation was displayed for 0.5 sec, followed by a feedback screen where a gold coin (“rewarded trial”) or a black coin (“unrewarded trial”) appeared at the center of the screen for 1 sec without the appearance of the chosen option.

Experiment 4B

In Experiment 4B, we modified the trial sequence from Experiment 3, such that after making their selection, participants saw the fixation screen for 0.5 sec, and then only the outcome appeared, without any indication of the chosen option.

### Data treatment

We omitted participants and trials from task analyses based on preregistered exclusion criteria. We excluded the first trial of each block, trials with no response, and trials with implausibly fast reaction times (< 300 ms) or unusually slow reaction times (> 4000 ms). This resulted in the omission of 7.3% of trials in the abstract sample and 5.2% in the story sample in Experiment 1, 7.8% of trials in Experiment 2, 5.8% of trials in Experiment 3, 5.3% of trials in Experiment 4 A, and 6.7% of trials in Experiment 4B. Participants were subsequently excluded from task analyses for the following reasons: (a) participants with more than 20% of trials omitted due to no response or implausibly fast reaction times were excluded from further analysis (Experiment 1: 26 participants in the abstract condition and 23 in the story condition; Experiment 2: 22 participants; Experiment 3: 6 participants; Experiment 4 A: 14 participants; Experiment 4B: 11 participants); (b) participants who repeated the same key response or switched responses on more than 70% of trials were excluded (Experiment 1: 3 participants in the abstract condition and 6 in the story condition; Experiment 2: 3 participants; Experiment 3: non-applicable; Experiment 4 A 2 participants; Experiment 4B: non-applicable); and (c) participants who showed evidence of switching to other browser windows based on mouse-tracking data (5 participants in Experiment 4 A). (d) Participants who did not complete all reinforcement learning sessions (15 in Experiment 2). As a result, the final sample in Experiment 1 included 250 participants in the abstract condition and 254 in the story condition, Experiment 2 included 158 participants, Experiment 3 included 195 participants, Experiment 4 A included 129 participants, and Experiment 4B included 190 participants.

### Working memory capacity estimate

To measure working memory capacity, participants were asked to maintain and retrieve visual information in a change detection task under varying load conditions^66,90,91^. On each trial, participants were shown a visual array of colored squares (set-size of 4 or 8 squares; see Supplementary Note 11). Squares in each array had distinct colors and were evenly spread across the screen. Each trial started with the presentation of the squares array (i.e., encoding phase, 200 ms), followed by a fixation cross (i.e., retention phase, 900 ms) and then a target (i.e., retrieval phase, until response with a 6-second deadline). The target screen included one colored square randomly selected by the computer in a color that was either the ‘same’ or ‘different’ color as the square that appeared in the same location in the memorized array. Participants were asked to respond ‘same’ or ‘different’ by pressing ‘s’ or ‘k’ response keys (keyboard mapping was counterbalanced between participants). Following the target screen, participants saw feedback indicating whether their response was correct or incorrect, followed by a fixation screen (inter-trial interval, 500 ms fixation). Each participant completed 120 trials aimed at identifying individual differences in working memory capacity. In each condition (set size 8/4), capacity was calculated according to *K*  =  *set_size ⋅ (2 ⋅ accuracy-1)*, and the average between the two conditions was used as a measure of overall capacity^90,149^.

We omitted participants and trials based on pre-registered exclusion criteria. A total of 524 participants in Experiment 1 and 178 participants in Experiment 2 completed the working memory task on the final day of testing. Participants were excluded based on the following preregistered criteria: (a) accuracy below chance on set size 4 (Experiment 1: 6 participants; Experiment 2: 10 participants), (b) higher accuracy on set size 8 than on set size 4 (Experiment 1: 15 participants; Experiment 2: 8 participants), and (c) evidence of switching to other browser windows during the task more than once (First time had a warning; Experiment 1: 14 participants; Experiment 2: 5 participants). Following these exclusions, the final sample included working memory data from 489 participants in Experiment 1 (453 had also valid data from the reinforcement learning task) and 155 participants in Experiment 2 (135 had also valid data from the reinforcement learning task).

### Computational modeling

To model the trial-by-trial updating of value representations, we employed a feature-based reinforcement learning framework, drawing on prior work^49,150–152^. In this model, separate Q-values were maintained for each action feature. One represented the outcome-relevant feature (i.e., color in Experiments 1, 2, and 4 A; location in Experiments 3 and 4B), and the other represented the outcome-irrelevant feature (i.e., location in Experiments 1, 2, and 4 A; color in Experiments 3 and 4B). Following each trial, prediction errors (denoted δ_f_) were computed for each selected feature using a standard temporal difference learning rule (see Eq.1), and these were then used to update the corresponding Q-values according to a learning rate α (Eq.2). We constrained the learning rate to be identical for the relevant and irrelevant features because allowing a separate α for the irrelevant dimension would introduce a non-identifiable α_irrelevant_ parameter when λ is estimated to be high. This issue can emerge in a substantial portion of participants whose λ values are close to 1.

1 $${\delta }_{f}=({reward}-{{\mbox{Q}}}_{f})$$

2 $${{\mbox{Q}}}_{f}={{\mbox{Q}}}_{f}+\alpha \cdot {\delta }_{f}$$

To integrate the learned feature values into the decision process, the model included a binary relevance vector based on the task instructions. This vector reflected which features were emphasized as relevant by the instructions. In the current study, the outcome-relevant feature varied across experiments. In Experiments 1, 2, and 4 A, the visual feature was outcome-relevant, and the spatial-motor feature was not. In Experiments 3 and 4B, the spatial motor feature was outcome-relevant, and the visual feature was not. Accordingly, the relevance vector was defined as assigning full relevance to the feature designated as outcome relevant in each experiment and none to the irrelevant feature (Eq.3).

3 $${{\rm{instructed}}}\_{{\rm{weights}}}=\left\{\frac{1\;{{\rm{if}}}\; {{\rm{feature}}}\; {{\rm{is}}}\; {{\rm{relevant}}}} {0\;{{\rm{if}}}\; {{\rm{feature}}}\; {{\rm{is}}}\; {{\rm{irrelevant}}}}\right.$$

The model then combined feature values according to this instruction-based relevance, modulated by a free parameter λ (Eq.4). This parameter controlled the degree to which the model followed the instructed relevance information. Specifically, the model computed an integrated relevance vector W as follows:

4 $${W}={{\rm{\lambda }}} \cdot {{\rm{instructed}}}\_{{\rm{weights}}}+(1-{{\rm{\lambda }}})\cdot (1-{{\rm{instructed}}}\_{{\rm{weights}}})$$

High values of λ indicate greater reliance on the instructed relevance, while lower values reflect a tendency to also consider the feature marked as irrelevant, thereby allowing for outcome-irrelevant learning. The λ parameter was constrained to the (0,1) interval using a logistic transformation.

In addition to value updating based on reward, the model included a mechanism to account for reward-independent tendencies to repeat or switch previously selected features. This was implemented by updating E-values for each chosen feature according to a feature-specific parameter (ρ_f_; Eq.5). Positive values of ρ reflected a bias toward repeating previously chosen features, whereas negative values captured a tendency to explore alternatives. Before each choice, the E-values decayed at a rate determined by a feature-specific parameter η_f_ (constrained between 0 and 1), giving greater weight to more recent choices (Eq.6).

5 $${{\rm{E}}}_{f}={{\rm{E}}}_{f}+{\uprho }_{f}$$

6 $${{\rm{E}}}_{f}={{\rm{E}}}_{f} \cdot {\upeta }_{f}$$

E-values were tracked separately for the visual and spatial motor domains, allowing the model to capture perseveration or switching tendencies within each modality.

The decision policy was based on a combination of value components. Q-values were weighted according to the W vector defined by λ and then scaled by an inverse temperature parameter β to account for random exploration (Eq.7).

7 $${{\rm{Q}}}_{{net}}=\beta \cdot {\sum }_{{f}} ({{\rm{w}}}_{f}\cdot {{\rm{Q}}}_{f})\,$$

Finally, the model combined Q-values and both E-values into a net value signal for each option. These signals were passed through a softmax function to produce the final choice probabilities (Eq.8).

8 $${{\rm{p}}} \, ({{\rm{choice}}}_{{\rm{i}}})=\frac{{{e}}^{Q}{{\rm{net}}}^{i+E}\,{{\rm{visual}}}^{i+{E}}{{\rm{spatial}}}^{i}}{{\sum }_{{\rm{j}}} {e}^{Q}{{\rm{net}}}^{j+E}{{\rm{visual}}}^{{j}+E}{{\rm{spatial}}}^{j}}\,$$

### Parameter recovery

**Fig. 9:**
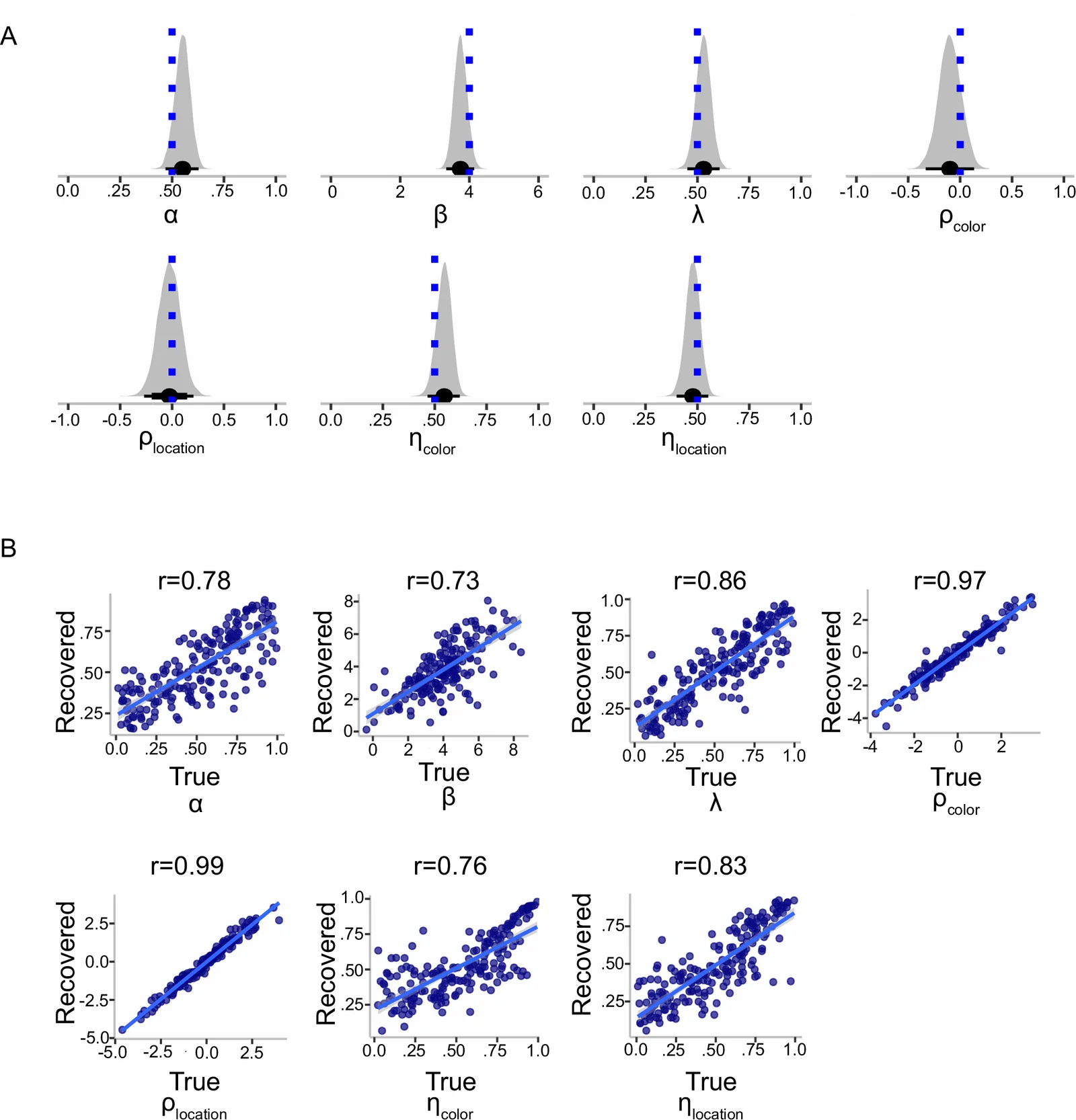
Parameter recovery. We simulated data for 200 agents completing the same multi-armed bandit task used empirically. We used a wide range of parameters to study whether we are able to recover these parameter values from simulated behavior. **A** We found excellent recovery on the population level, where each gray density corresponds to posterior samples for the population parameter, and the blue line indicates the true generative parameter. The black circle represents the posterior estimated median, and the horizontal line the 95% CI. **B** Furthermore, we found excellent recovery at the individual level, evidenced by the high correlations between true and recovered individual parameters. Each blue dot represents a simulated agent, and the blue line represents the regression line with 95% CI shown as a shaded area. Thus, we conclude our model is capable of identifying latent parameters from behavior.

To evaluate our model’s validity, we performed a parameter recovery analysis to test whether known generative parameters could be accurately recovered from simulated data^73^. We simulated behavior for 200 agents, each assigned a diverse set of parameters sampled from broad distributions [α ∼ N(0, 1.5); β ∼ N(4, 1.5); λ ∼ N(0, 1.5); ⍴_spatial_ ∼ N(0, 1.5); ⍴_visual_ ∼ N(0, 1.5); η_spatial_ ∼ N(0, 1.5); η_visual_ ∼ N(0, 1.5)]. Parameters α, λ, η_spatial_, and η_visual_ were logistically transformed to constrain their values between 0 and 1. Simulation data had 4 blocks with 50 trials in each and used one of the empirical random-walk sets. The results showed strong parameter recovery at both the population and individual levels. For each parameter, we found high correlations between the true (simulated) and estimated values across individuals (see Fig. 9), supporting the model’s reliability and justifying its use with empirical data. See Supplementary Note 9 showing that the parameters are identifiable in empirical data.

### Model recovery

Before applying model comparison to empirical data, we conducted a model recovery analysis to validate our modeling approach and ensure that the statistical procedure could reliably identify the true generative model^73^. In this analysis, we simulated data from 200 agents using either the reduced model or the full model. We then estimated out-of-sample predictive accuracy using leave-one-out cross-validation (LOO-CV) via the loo package, calculating the expected log predictive density (elpd) for each trial using Pareto-smoothed importance sampling. Following established guidelines, we interpreted elpd differences greater than twice their standard error (SE) as evidence of a meaningful distinction between models^72,74,75^. The analysis confirmed that models are recoverable. Specifically, the full model provided a markedly better fit when it served as the generative source (elpd difference = 1553, SE = 52). When data was generated using the reduced model, the full model accurately estimated λ to be redundant (λ posterior median ~ 1, where λ = 1 equals the reduced model; see Supplementary Note 6). Thus, as expected^74^, predictive out-of-sample performance between the two models was equal (elpd difference = 0.5, SE = 0.8).

### Bayesian parameter estimation

Bayesian logistic regression and reinforcement learning computational modeling analyses were performed using “brms” and “cmdstan” packages in R^71,153^. Both the computational and regression models included population-level (fixed effects) and individual-level (random effects) parameters. The models were sampled with weakly informative priors: N ~ (0,1) for regression coefficients, N ~ (0,3) for computational model population-level parameters, and N ~ (0,2) for individual-level parameters. We also examined wider priors (i.e., N ~ (0,2) and N ~ (0,4)) and found that none of the conclusions in the manuscript were sensitive to the chosen priors. All chains were visually examined using trace plots, pairs plots, and R-hat estimates and were found to show good convergence. Instead of relying on a binary criterion, such as checking whether zero is excluded from the highest density interval, we quantified the strength of each dynamical effect using the probability of direction (pd), which represents the proportion of the posterior distribution that shares the same sign as the median value of the estimate. We report the median, HDI_95%_, and probability of direction for parameters’ posterior distributions (for logistic regression, estimates are on the log-odds scale^85,154,155^). Fitting results had 2000 samples for each of the 4 chains, allowing us to estimate uncertainty in population-level and individual-level parameter estimates.

### Reporting summary

Further information on research design is available in the Nature Portfolio Reporting Summary linked to this article.

## Supplementary information

SUPPLEMENTARY INFO

Reporting Summary

Transparent Peer Review file

## Data Availability

All the data can be found online at the following GitHub repository: https://doi.org/10.5281/zenodo.21377105. The repository includes the raw task data and the preprocessed data frames necessary to conduct all analyses shown in the manuscript. A link to the data repository for the reanalysis of the two-step task^45^ can also be found in the link.

## Code availability

All the code required to recreate the analyses in this manuscript can be found online at the following GitHub repository: https://doi.org/10.5281/zenodo.21377105. In the repository, you will find the code necessary to run all analyses and create all figures. We also share the code required to run the tasks.
