# Supplementary material for: Automatic value learning results in counterproductive human behavior
Source: Nat Commun. 2026 Aug 10;17:9567. doi: 10.1038/s41467-026-76297-7 (PMC13550625; doi:10.1038/s41467-026-76297-7)
Supplement: Supplementary file 1 [file 41467_2026_76297_MOESM1_ESM.pdf]

Supplementary Information File

Supplementary Note 1 - Outcome-irrelevant learning is associated with reduced gains

Previous studies have claimed that drifting probabilistic rewards might not be sensitive to the use of optimal strategies [1]. We therefore further examined whether outcome-irrelevant learning was associated not only with reduced accuracy but also with lower payoffs in the task, which are what participants observed in the task. We found a substantial association with lower λ resulting in lower payoffs (posterior median = .06, HDI95% [.03, .09], pd =~100%) and Experiment 2 (posterior median = .08, HDI95% [.03, .12], pd =~100%; see Figure S1). Note that in Figure S1, the different intercepts between Experiment 1 & 2 pertain to the changes in the random walk, while the association between lambda and mean payoffs (i.e., slope) remains the same.

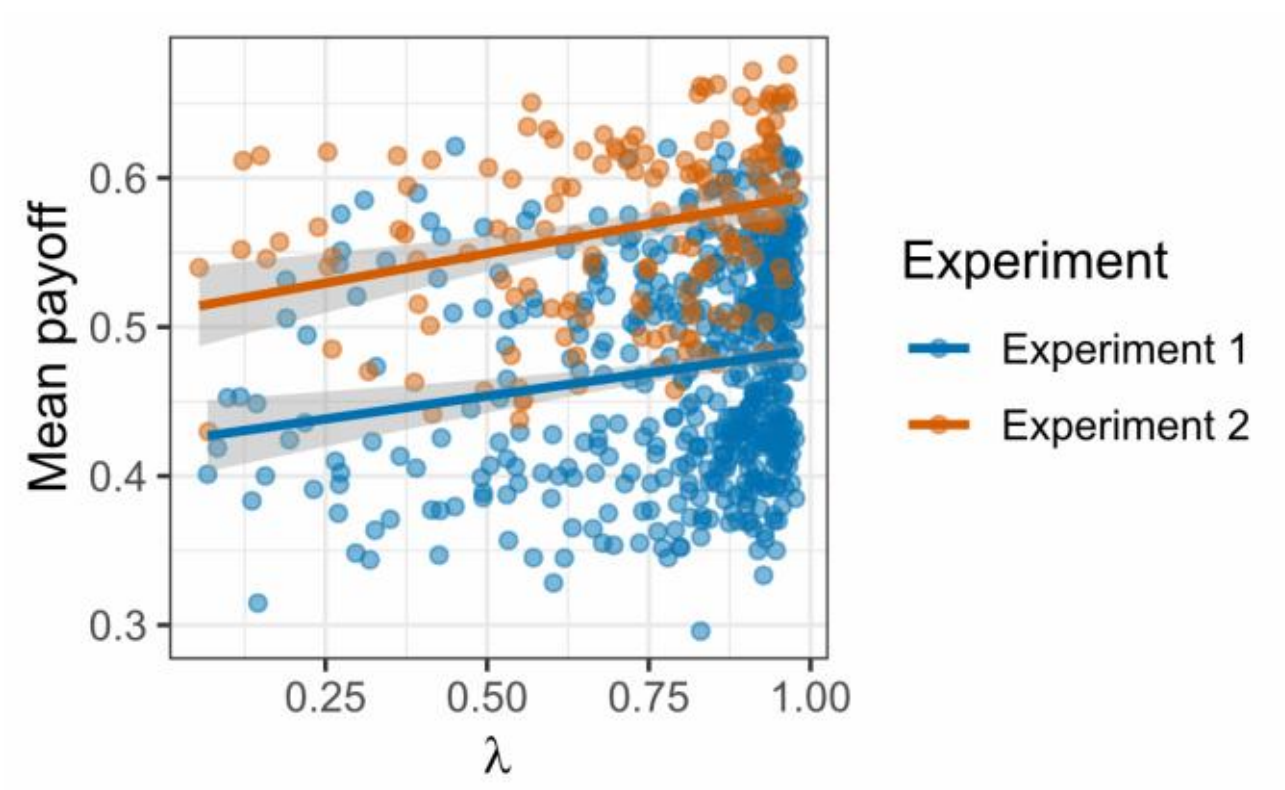

Figure S1. The association of λ with mean payoffs in the task. We find a positive association between λ and the mean payoff in the task. Note that the different intercepts are due to the different random walk sets used in the two experiments, and do not reflect a difference in the association between λ and mean payoffs, which was similar in both experiments. Blue dots represent data from Experiment 1 (N=504) and orange dots represent data from Experiment 2 (N=158), and the regression line is depicted in color. Error bars represent the 95% CI.

Supplementary Note 2 - Outcome-irrelevant learning beyond the previous trial is not due to a statistical artifact

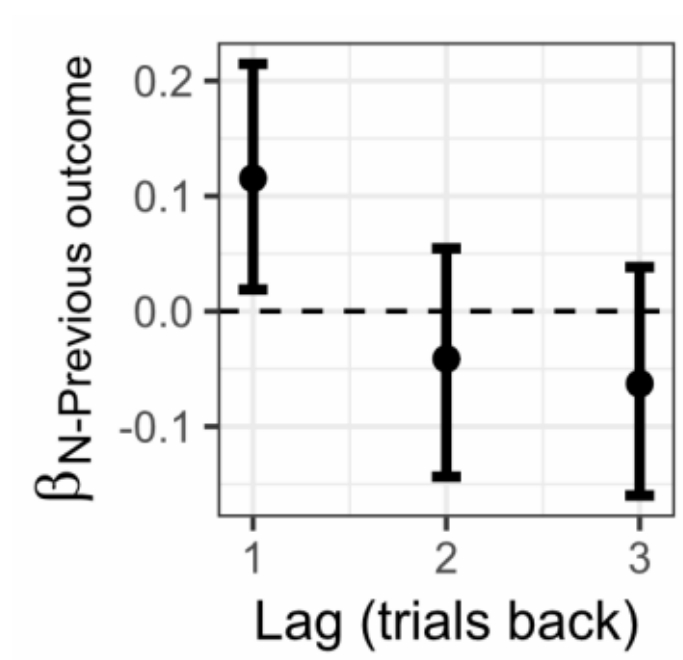

Figure S2. Validating the ability of our model-agnostic signature regression analysis to estimate outcome-irrelevant learning from two and three trials back. The main text presents two regression analyses examining the influence of outcome on the selection of an outcome-irrelevant location, from two and three trials back (Figure 3A, main text). We wanted to ensure that if participants had been only influenced by the very last trial (*n-1*) and not the two or three trials back (*n-2*/*n-3*), then our regression

coefficients would accurately reflect that. For this purpose, we simulated 200 agents with empirically derived parameters, but whose learning rate was artificially set to 1, reflecting learning that is constrained only to the very last trial (*n-1*). This means these agents should not show an effect on the n-2 or n-3 by definition, allowing us to test whether the regression analysis can, in fact, discover that. As expected, when fitting the logistic regression to data from the simulations, we find that only the outcome of the very last trial affects the probability of repeating the same location (in trial *n*), with no residual influence from earlier trials.

Supplementary Note 3 - Replication using previously published data from Feher da Silva et al. (2023)

To replicate the findings of Experiment 1, we re-analyzed the dataset reported by Feher da Silva et al. [2]. In that study, 50 adults completed a two-step decision task under story-based instructions' framing [3]. On each trial, participants made a first-stage choice that stochastically determined which pair of second-stage options would appear. In the second stage, participants selected one of the two offers, which then led to a reward with some unknown probability. The authors framed the first-stage choice in the instructions as a selection between two magical carpets that would each lead the participant to one of two mountains with some probability. The choices at the second stage were framed as two genie lamps that were placed in each mountain, and when rubbed, might release a genie who would provide a reward with an unknown probability. The reward probability was framed by telling participants that some genies are more likely than others to enjoy emerging from their lamps, making some lamps more profitable to rub than others. Crucially, carpets and lamps were randomly assigned to the left or right side of the screen, and this location had no bearing on the reward probabilities. Participants were explicitly told that carpets randomly change places, and that after rubbing a genie lamp, it might be returned to either the left or right side in the mountain's cave, thereby emphasizing that the location was arbitrary and carried no reward-predicting information.

We found clear evidence of outcome-irrelevant learning under the story-based instructional framing. Specifically, we fitted a hierarchical Bayesian logistic regression measuring the likelihood that participants will repeat the location of the previously selected lamp during their next first-stage (carpet) choice, as a function of reward. Results revealed a clear outcome-irrelevant learning effect. Participants were more likely to choose (in stage 1 of trial *n*) the carpet appearing in the same location as the previously chosen lamp (in stage 2 of trial *n-1*) when the previous choice was rewarded (50% location stay-probability) compared to when it was not rewarded (47% location stay-probability; posterior median for the effect is .13, $HDI_{95\%}$ [.03, .22], pd = 99.5%; see Figure S3A-B). Importantly, this reward-dependent repetition effect cannot be explained by motor perseveration, which refers to the tendency to repeat previous actions regardless of the outcome. Rather, our analysis reveals a reward-dependent effect in which value is assigned to spatial locations, such that previously rewarded location choices are more likely to be repeated in a different state in the following trial, even when locations are not predictive of rewards.

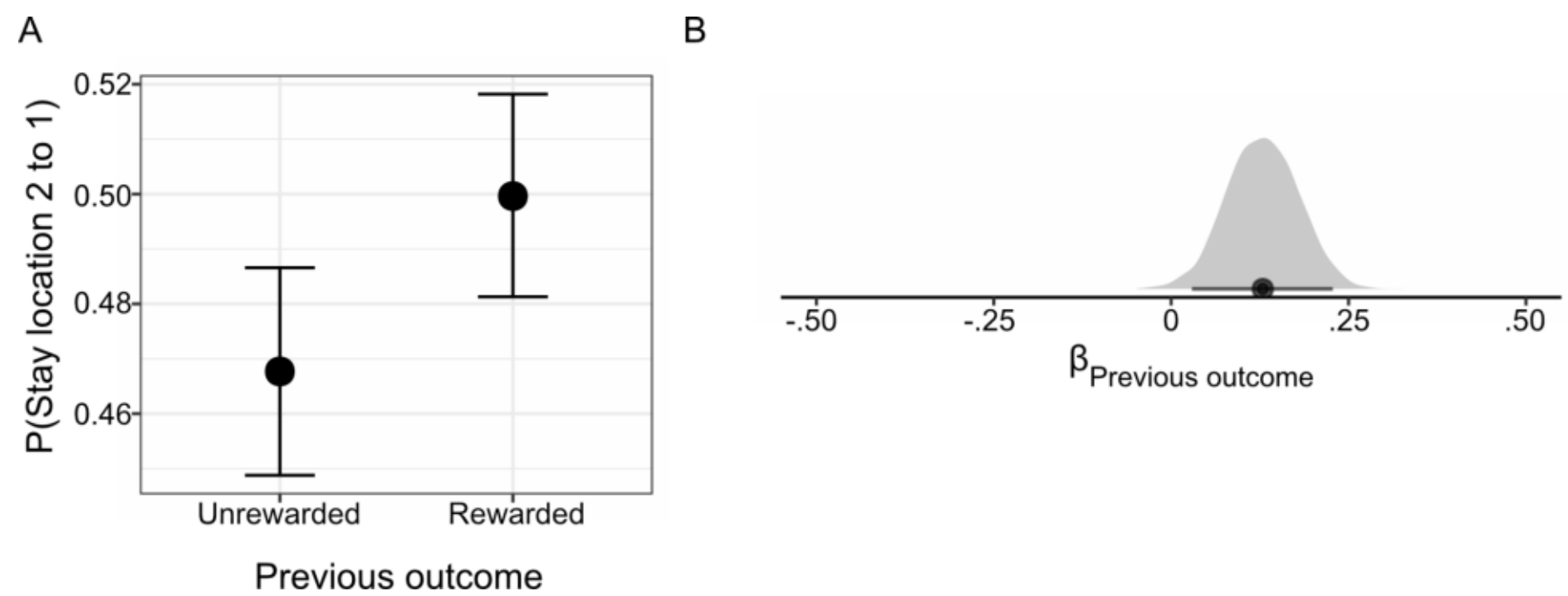

Figure S3. Replication of outcome-irrelevant learning despite narrative instructions using a re-analysis of Da Silva & Hare (2023). We reanalyzed a previously published dataset in which participants (N=50) completed a two-step decision task designed to dissociate model-based from model-free learning. See Figure 1 in [2] for a visual depiction of the trial sequence. In each trial, participants selected between two carpets, leading probabilistically to a second-stage choice between two genie lamps. The goal was to maximize rewards by choosing the more rewarding genie. Critically, the spatial locations of options were fully randomized across trials, making any learning based on location outcome-irrelevant. The original authors argued that the story-like instructions promoted purely model-based behavior, minimizing model-free influences. **(A)** Our reanalysis reveals clear evidence of outcome-irrelevant learning. Participants showed a higher tendency to repeat the same location from the previous second-stage choice if it had been rewarded than if it was not, despite locations being randomized and thus non-informative. The black circle denotes the posterior median, and the error bars represent the 95% CI. **(B)** Population-level posterior estimate of the reward effect on location repetition. Following a rewarded trial, participants were more likely to stay with the same location choice (trial *n* second-stage choice to trial *n+1* first-stage choice) than after an unrewarded trial. The black circle denotes the posterior median, and the horizontal line indicates the 95% CI.

Supplementary Note 4 - Outcome-irrelevant learning is not limited to decisions where no information is available in the outcome-relevant feature

To test whether outcome-irrelevant learning only serves as a tie-breaking mechanism, we explored whether the model-agnostic regression signature is also apparent when the outcome-relevant feature provides a strong signal for choice. Specifically, we repeated the regression analysis on a subset of trials where the underlying expected values difference of the two colors was large (>0.3) and found that even in these trials participants are more likely to repeat a previously chosen location following a rewarded compared to an unrewarded trial (posterior median = .2, HDI95% [.1, .3], pd~=100%; see Figure S4A). As participants may not be aware of the underlying expected values, we show similar results on a subset of trials where the difference in model-derived Q-values for the offered colors is large (>0.3), and found the same effect (posterior median = .24, HDI95% [.13, .35], pd=~100%; see Figure S4B). While these analyses indicate that outcome-irrelevant learning also occurs when the outcome-relevant feature is far from chance-level, we acknowledge that the general conclusion that outcome-irrelevant learning is not merely a tie-breaking mechanism is constrained by the assumption that our estimates accurately reflect the values held by participants. Future experiments should therefore use designs that more directly test this hypothesis.

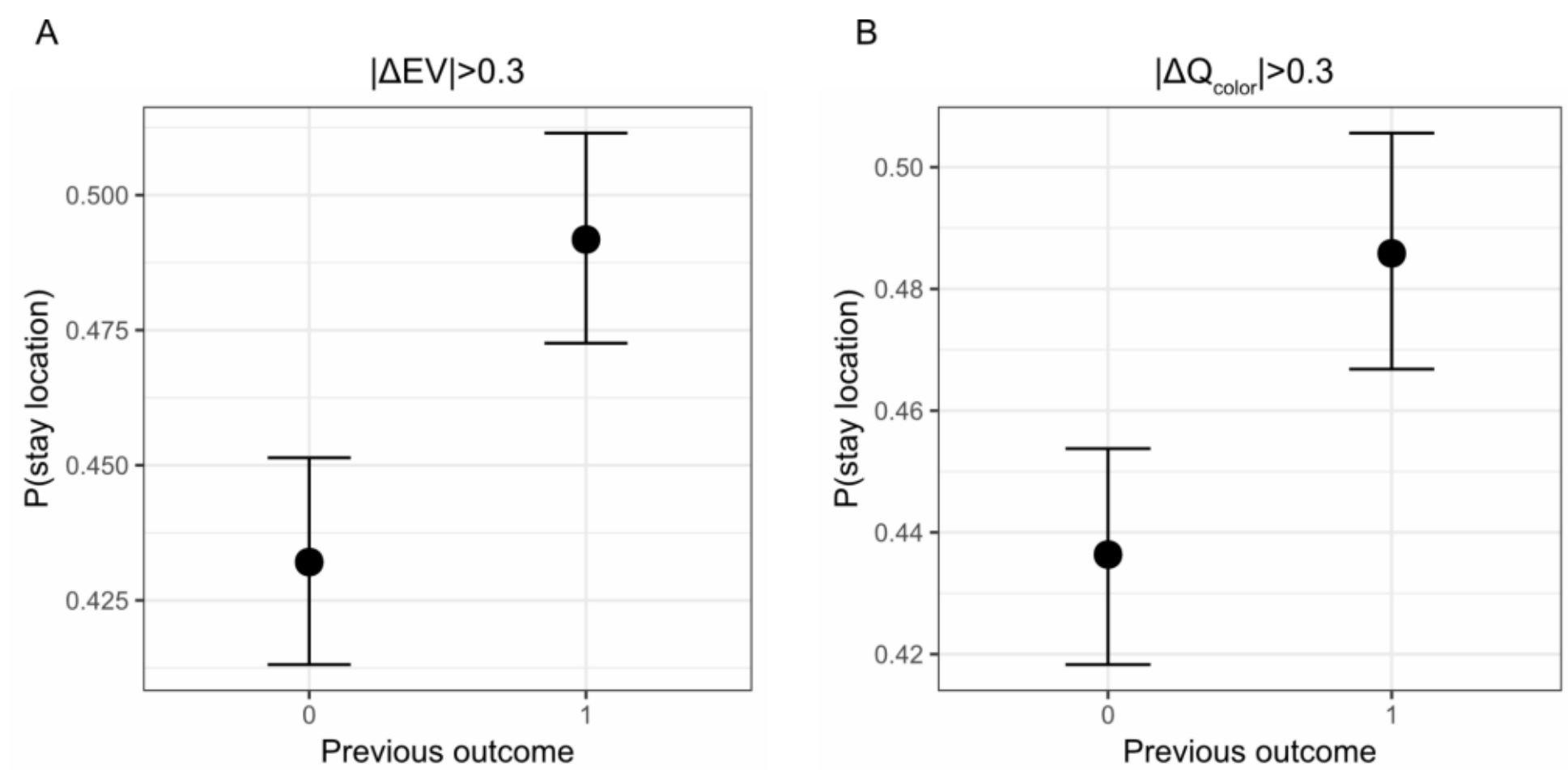

Figure S4. Outcome-irrelevant learning in trials with clear outcome-relevant information. (A) We also find that in a subset of "easy" trials where the current difference in expected values between the colors is large (i.e., >0.3), we find a clear indication of the outcome-irrelevant model-agnostic effect. **(B)** As the underlying expected values of the random walk are not available for participants, we repeated the same analysis with model-derived Q-values for the outcome-relevant feature and found the same effect. Data from Experiment 1 (N=504).

Supplementary Note 5 - Model-estimated Q-value difference in Experiments 1 and 2

A concern is that the Q-values differences for outcome-irrelevant dimensions might show restricted variance since they are driven by a random association with the outcome (due to their irrelevant nature by design). We therefore examined and found that the differences in Q-values of the two outcome-irrelevant locations were substantial when estimating them from the model (see Figure S5). Thus, while arbitrary in nature, outcome-irrelevant values have a predictable influence on behavior.

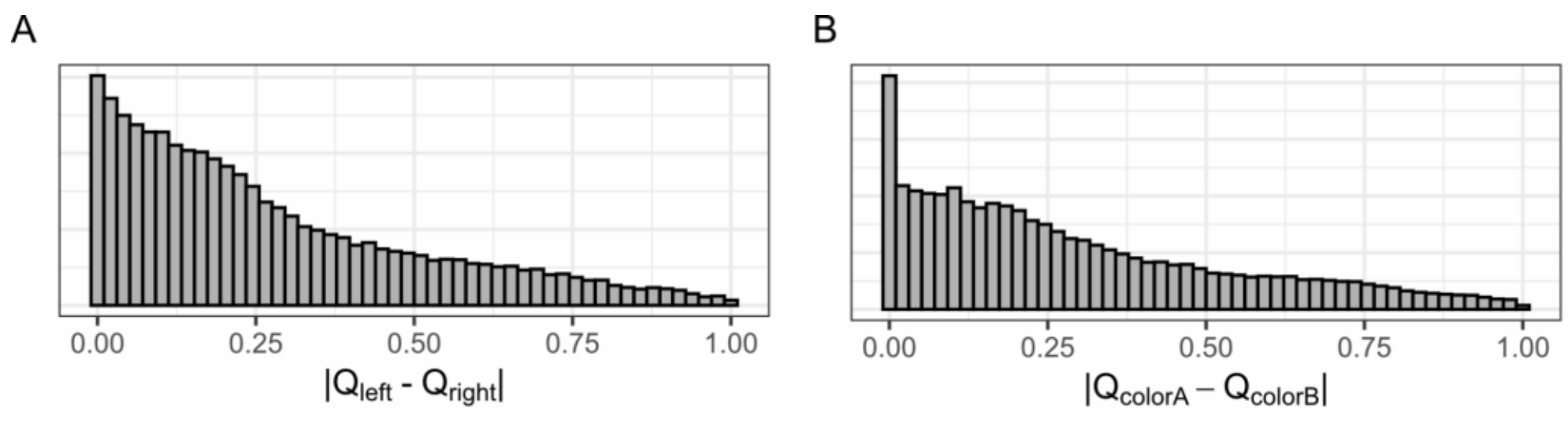

Figure S5. A histogram for the absolute model-derived Q-value difference between the (A) Left and Right outcome-irrelevant locations, and (B) between the two outcome-relevant currently offered colors. This shows that the variance of outcome-irrelevant Q-value differences was not minimal, and could show substantial value differences, despite being driven by random association with reward. Data from Experiment 1 (N=504) and Experiment 2 (N=158).

Supplementary Note 6 - The estimation of λ as lower than 1 is not due to statistical capping artifacts

To illustrate that our model fitting procedure can identify data containing no outcome-irrelevant learning, we simulated data of 50 agents from the reduced model, where λ is 1 for all agents, using the design from Experiment 1. We find that our model fitting procedure confidently recovered population-level λ as 1 (posterior median = .997), indicating that capping effects of λ are negligible.

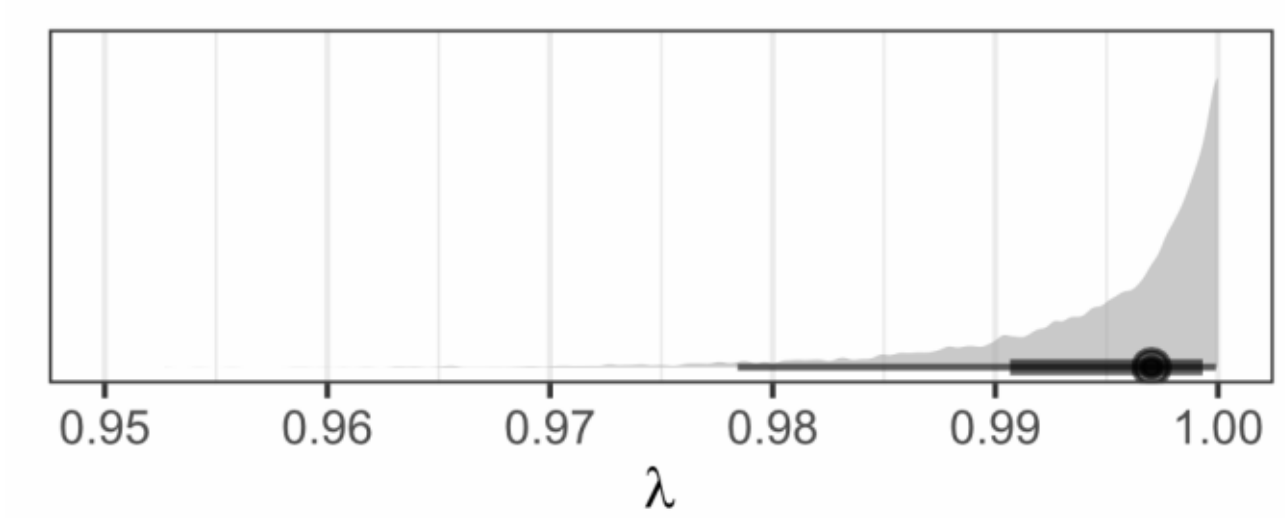

Figure S6. **Recovery of population-level λ = 1.** We simulated 50 agents with λ = 1 and estimated the posterior population-level λ. We found that λ can be clearly recovered (posterior median = .997).

Supplementary Note 7 - Recovery of λ in agents with λ=1 and attentional lapses

To show that inattentive responding can not account for the outcome-irrelevant learning effect, we simulated data of 50 agents using empirically derived parameters, but eliminated outcome-irrelevant learning in those agents by fixing λ to 1. We further included attentional lapses in one third of the trials for all agents by temporarily setting β = 0. Yet, we were able to recover λ as equal to 1 (posterior median = .99, HDI95% [.93, 1]; see Figure S7).

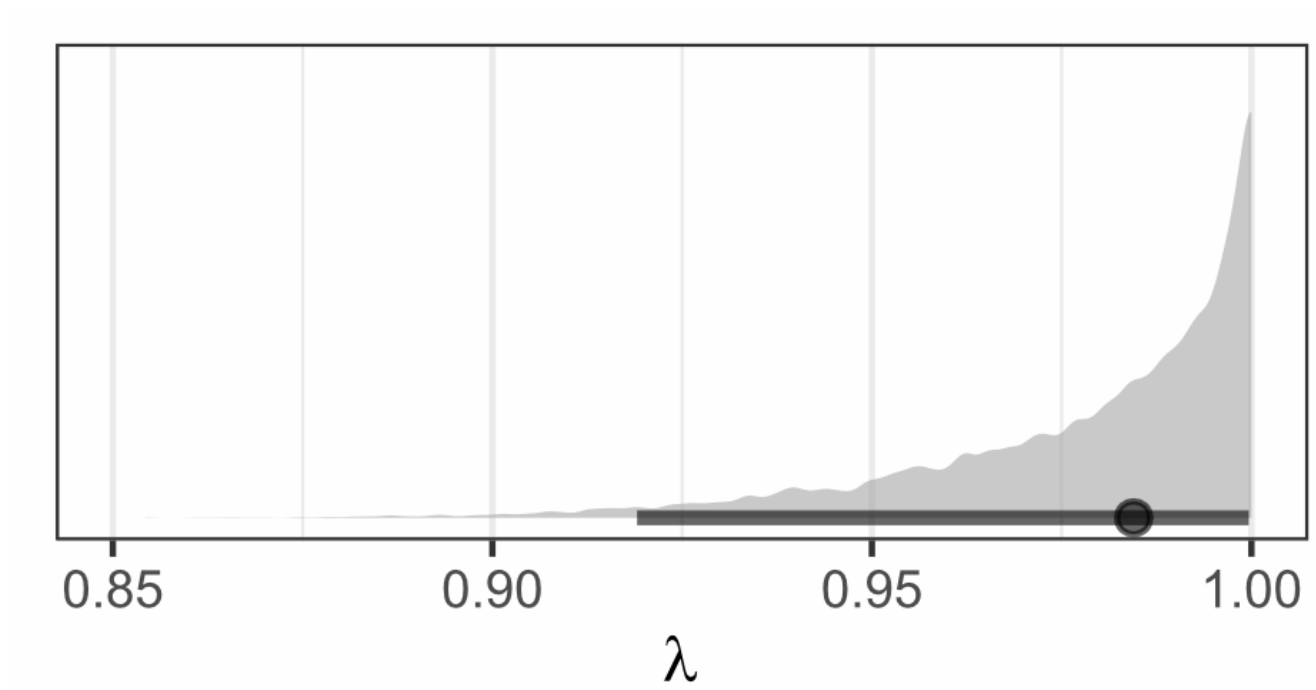

Figure S7. Recovery of λ for "inattentive" agents. We simulated data of 50 agents with empirically derived parameters, but whose λ is fixed to 1. We further introduced "attentional lapses" by temporarily setting $\beta = 0$ on one third of the trials. We find that even under those conditions, of occasional value-independent choice, λ is still recoverable.

Supplementary Note 8 - The association between λ and the model-agnostic outcome-irrelevant learning signature is not due to misestimation in random responders

To ensure that λ genuinely reflects outcome-irrelevant learning even among individuals who are not simply behaving noisily, we performed an additional analysis restricted to participants with β values above the median ($\beta > 1.8$). This subset represents individuals who show relatively strong value-based choice consistency, reducing the possibility that λ merely compensates for randomness in behavior. Even within this higher-β group, λ was strongly associated with the model-agnostic signature of outcome-irrelevant learning (r=-.55; see Figure S8), reinforcing that λ captures a meaningful computational tendency rather than an artifact of low β.

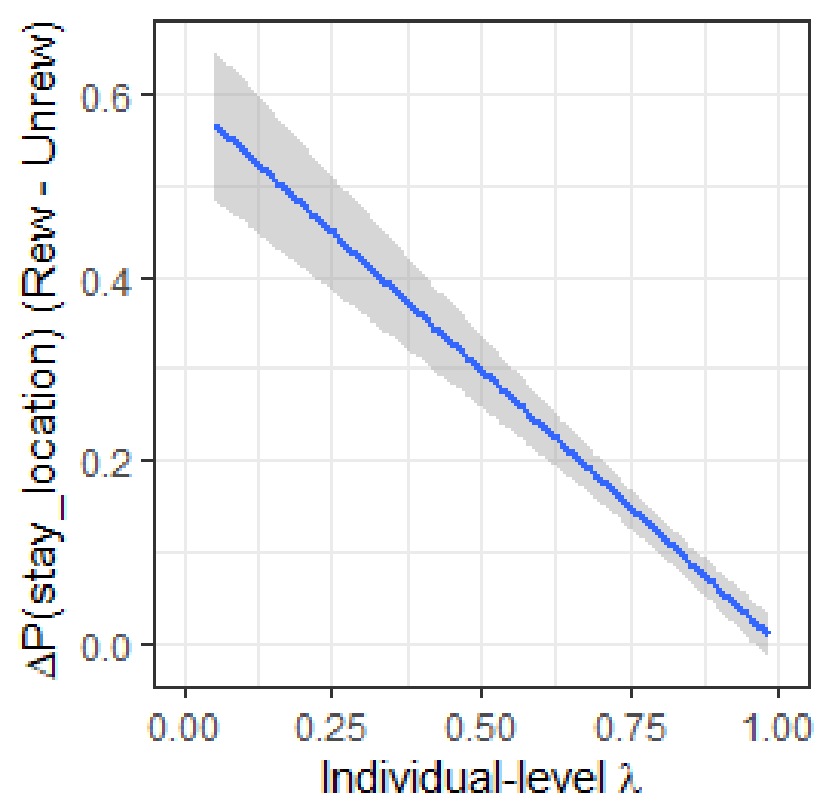

Figure S8. The association between λ and the model-agnostic outcome-irrelevant learning effect persists even when restricting the analysis to participants with high β values (β above the median). Data from Experiment 1 (N=504) and Experiment 2 (N=158).

Supplementary Note 9 - Empirical parameter identifiability

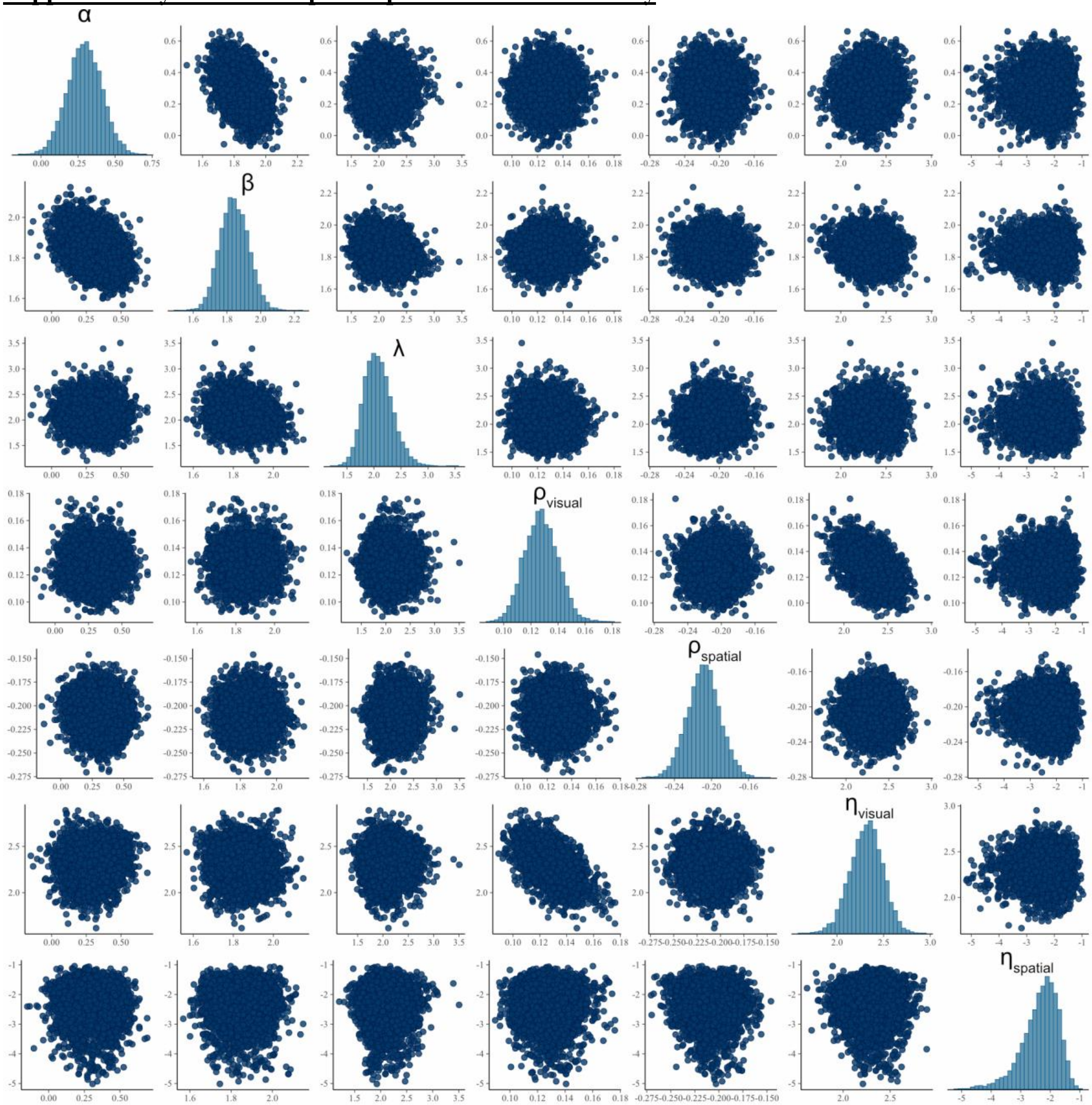

Figure S9. MCMC posterior sampling empirical pairs plot. To assess potential trade-offs between the outcome-irrelevant learning parameter λ and other estimated model parameters, we examined pairs plots of MCMC posterior samples for population-level parameters in the abstract condition of Experiment 1. The plots show a unimodal posterior distribution for all model parameters, and only weak correlations between λ and inverse temperature β as well as between λ and other parameters, indicating limited parameter trade-offs and good identifiability.

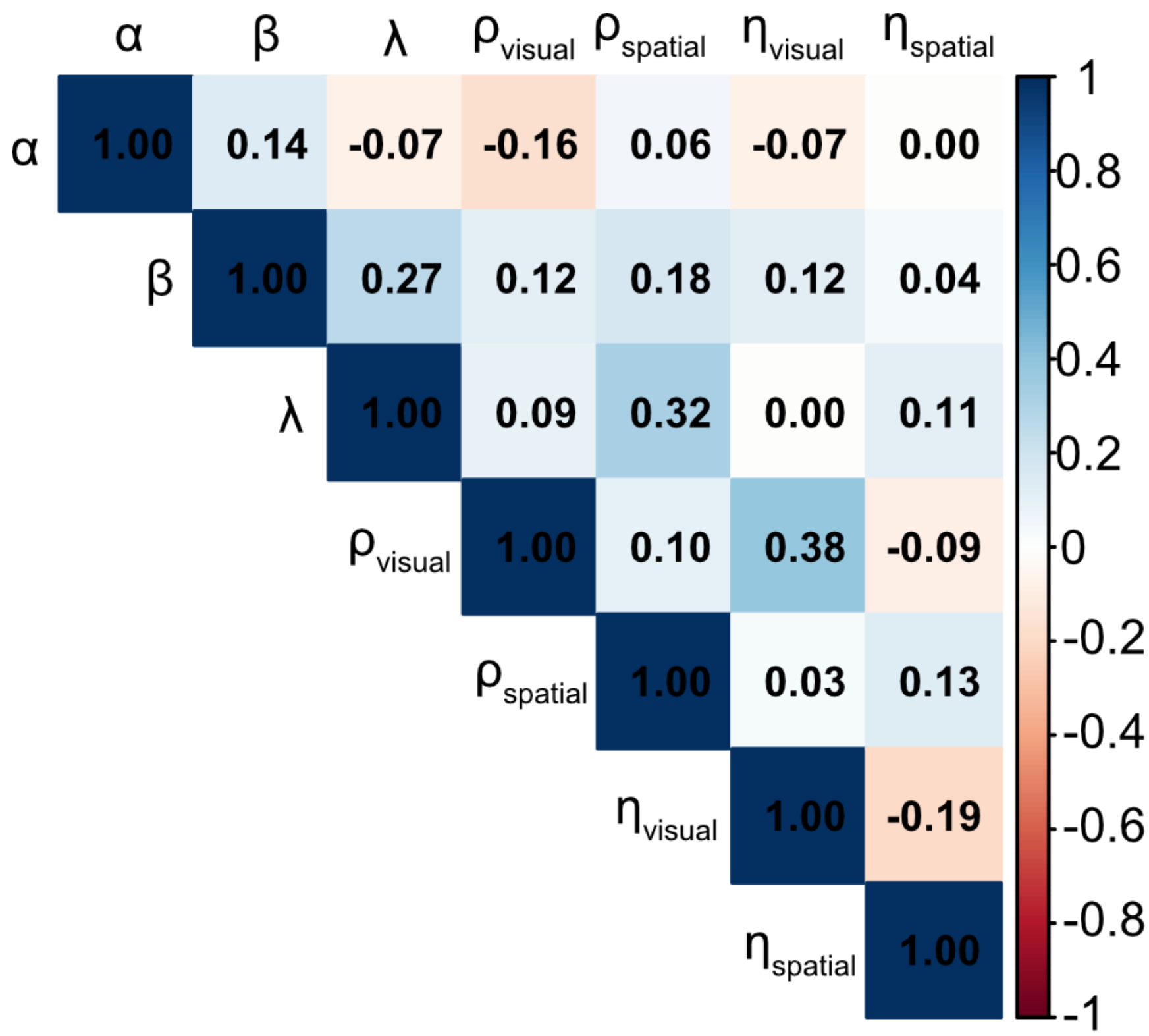

Figure S10. Correlations between individual-level parameter estimates. To examine empirical associations between individual-level parameter estimates, we computed Pearson correlations between individuals' posterior mean parameters across Experiments 1 and 2. Correlations were generally weak, indicating that individual differences in the outcome-irrelevant learning parameter λ did not strongly depend on individual differences in other model parameters.

Supplementary Note 10 - Comparing the computational model to a simpler categorical regression model

Previous studies indicated that a categorical regression model can fit the data better than RL models, indicating that participants' true strategies might deviate substantially from the learning approaches specified in an RL model. We therefore compared whether our winning computational model leads to a better fit than a simpler logistic regression, where choice (i.e., which of the two colors is chosen) is predicted only by the previous outcome (unrewarded/rewarded). Given that only two out of four colors are offered on each trial, we constrained our comparison to predicting choices on trials in which the previously chosen color was reoffered. We found that the computational model predictions were markedly better than the ones made by a categorical regression capturing only win-stay lose-shift behavior for the chosen color (Experiment 1 Story Δelpd = 966, SE = 66).

Supplementary Note 11 - Change detection task

The size of each square was 1.3° x 1.3° (all visual angles assume a 60cm distance of the participant from the screen), and the colors of the squares were randomly picked in each trial from 9 highly discriminable colors: red, magenta, blue, cyan, green, yellow, orange, brown, and black. (RGB values, respectively: 254,0,0; 255,0,254; 0,0,254; 0,255,255; 0,255,1; 255,255,0; 255,128,65; 128,64,0; 0,0,0). The task included two conditions varying trial-by-trial (set size 4 or set size 8). The squares were equally distributed around the four quadrants of the screen. Within each quadrant, one (set size 4) or two (set size 8) square locations were randomly selected by the computer out of three possible locations in every quadrant of the screen. To take into account the fact that the experiment was made on participants' personal computers, locations were on absolute distances compared to the center of their screen. The locations were 2.7°,5.3°, or 8° horizontally and vertically from the center of the screen, in each of the quadrants: Q1: (-2.7°,2.7°);(-5.3°,5.3°);(-8°,8°;) Q2:(2.7°,2.7°);(5.3°,5.3°);(8°,8°) Q3: (2.7°,-2.7°); (5.3°,-5.3°); (8°,-8°) Q4: (-2.7°,-2.7°);(-5.3°,-5.3°); (-8°,-8°).

Table S1. Posterior parameter estimates across three sessions of Experiment 2.

Parameter	Session 1	Session 2	Session 3	ΔSession 2 - 1	ΔSession 3 - 2
α(logit)	0.26 [-0.08, 0.60]	0.39 [-0.01, 0.77]	0.29 [-0.15, 0.74]	0.13 [-0.37, 0.66]	-0.10 [-0.70, 0.49]
β	1.84 [1.63, 2.04]	1.65 [1.45, 1.86]	1.71 [1.50, 1.91]	-0.19 [-0.47, 0.11]	0.05 [-0.24, 0.34]
η_{visual}(logit)	3.16 [2.84, 3.48]	2.64 [2.28, 3.00]	2.79 [2.32, 3.21]	-0.52 [-1.02, -0.06]	0.15 [-0.42, 0.75]
$\eta_{spatial}$(logit)	-1.37 [-2.26, -0.61]	-1.86 [-2.93, -1.03]	-3.42 [-5.18, -1.88]	-0.50 [-1.85, 0.86]	-1.53 [-3.61, 0.41]
ρ_{visual}	0.11 [0.09, 0.13]	0.15 [0.12, 0.18]	0.15 [0.12, 0.18]	0.04 [0.01, 0.08]	-0.01 [-0.05, 0.03]
$\rho_{spatial}$	-0.21 [-0.25, -0.16]	-0.25 [-0.30, -0.20]	-0.20 [-0.26, -0.14]	-0.04 [-0.11, 0.03]	0.05 [-0.03, 0.12]
λ(logit)	2.08 [1.34, 2.89]	1.99 [1.21, 2.92]	1.57 [0.84, 2.37]	-0.08 [-1.26, 1.12]	-0.42 [-1.64, 0.70]

Note. Values represent unconstrained (i.e., before logistic transformation for α, η, and λ) posterior medians with 95% HDIs in brackets.

Supplementary Note 12 - Instructions and Quiz across Experiments

PowerPoint presentations visually depicting the instructions used across experiments, including those in which locations were outcome irrelevant and those in which colors were outcome irrelevant, can be found on GitHub at the following link: https://github.com/shahar-lab/Automatic-value-learning-results-in-counterproductive-human-behavior/tree/main/Task%20instructions

Supplementary References

1. Kool, W., Cushman, F. A. & Gershman, S. J. When Does Model-Based Control Pay Off? *PLOS Comput. Biol.* **12**, e1005090 (2016).

2. Feher da Silva, C., Lombardi, G., Edelson, M. & Hare, T. A. Rethinking model-based and model-free influences on mental effort and striatal prediction errors. *Nat. Hum. Behav.* **7**, 956–969 (2023).

3. Daw, N. D., Gershman, S. J., Seymour, B., Dayan, P. & Dolan, R. J. Model-Based Influences on Humans' Choices and Striatal Prediction Errors. *Neuron* **69**, 1204–1215 (2011).
