# Supplementary material for: Automatic value learning results in counterproductive human behavior
Source: Nat Commun. 2026 Aug 10;17:9567. doi: 10.1038/s41467-026-76297-7 (PMC13550625; doi:10.1038/s41467-026-76297-7)
Supplement: Supplementary file 2 [file 41467_2026_76297_MOESM2_ESM.pdf]

nature portfolio

Corresponding author(s): Ido Ben-Artzi

Last updated by author(s): 12/01/2026

Reporting Summary

Nature Portfolio wishes to improve the reproducibility of the work that we publish. This form provides structure for consistency and transparency in reporting. For further information on Nature Portfolio policies, see our Editorial Policies and the Editorial Policy Checklist.

Statistics

For all statistical analyses, confirm that the following items are present in the figure legend, table legend, main text, or Methods section.

n/a	Confirmed	
☐	☒	The exact sample size (n) for each experimental group/condition, given as a discrete number and unit of measurement
☐	☒	A statement on whether measurements were taken from distinct samples or whether the same sample was measured repeatedly
☒	☐	The statistical test(s) used AND whether they are one- or two-sided<br>*Only common tests should be described solely by name; describe more complex techniques in the Methods section.*
☐	☒	A description of all covariates tested
☐	☒	A description of any assumptions or corrections, such as tests of normality and adjustment for multiple comparisons
☐	☒	A full description of the statistical parameters including central tendency (e.g. means) or other basic estimates (e.g. regression coefficient) AND variation (e.g. standard deviation) or associated estimates of uncertainty (e.g. confidence intervals)
☒	☐	For null hypothesis testing, the test statistic (e.g. F, t, r) with confidence intervals, effect sizes, degrees of freedom and P value noted<br>*Give P values as exact values whenever suitable.*
☐	☒	For Bayesian analysis, information on the choice of priors and Markov chain Monte Carlo settings
☐	☒	For hierarchical and complex designs, identification of the appropriate level for tests and full reporting of outcomes
☐	☒	Estimates of effect sizes (e.g. Cohen's d, Pearson's r), indicating how they were calculated

Our web collection on statistics for biologists contains articles on many of the points above.

Software and code

Policy information about availability of computer code

Data collection	Participants were recruited through the Prolific platform and directed to a task webpage hosted on the Pavlovia server. The experiment was implemented using the jsPsych package (v7.1.2). All code required to run the tasks is publicly available at the following github repository: https://doi.org/10.5281/zenodo.21377105
Data analysis	Data were analyzed in R (v4.3.1) using the brms (v2.22), cmdstanr(v0.8.1), and loo(v2.8) packages. Stan models were run through the RStudio interface. All the code required to recreate the analyses in this manuscript can be found online at the following github repository: https://github.com/shahar-lab/Automatic-value-learning-results-in-counterproductive-human-behavior<br>In the repository you will find the code necessary to run all analyses and create all figures.

For manuscripts utilizing custom algorithms or software that are central to the research but not yet described in published literature, software must be made available to editors and reviewers. We strongly encourage code deposition in a community repository (e.g. GitHub). See the Nature Portfolio guidelines for submitting code & software for further information.

Data

Policy information about availability of data

All manuscripts must include a data availability statement. This statement should provide the following information, where applicable:

- Accession codes, unique identifiers, or web links for publicly available datasets
- A description of any restrictions on data availability
- For clinical datasets or third party data, please ensure that the statement adheres to our policy

All the data can be found online at the following github repository:
https://doi.org/10.5281/zenodo.21377105
The repository includes the raw task data, and the preprocessed data frames necessary to conduct all analyses shown in the manuscript. Data used for the reanalysis of Feher da Silva et al. (2023) can be found at https://www.nature.com/articles/s41562-023-01573-1 .

Research involving human participants, their data, or biological material

Policy information about studies with human participants or human data. See also policy information about sex, gender (identity/presentation), and sexual orientation and race, ethnicity and racism.

Reporting on sex and gender	Sex and gender information was obtained from Prolific and is reported descriptively in the Methods section. No sex- or gender-based analyses were conducted.
Reporting on race, ethnicity, or other socially relevant groupings	Ethnicity was obtained from Prolific but is not currently reported in the Methods section. No ethnicity-based analyses were conducted.
Population characteristics	Participants were between 18 and 50 years old and reported no history of psychiatric or neurodevelopmental conditions.
Recruitment	Participants were recruited anonymously through the Prolific platform, an online crowdsourcing service for research.
Ethics oversight	The study protocol for all experiments was approved by the Research Ethics Council of Tel Aviv University (#6707).

Note that full information on the approval of the study protocol must also be provided in the manuscript.

Field-specific reporting

Please select the one below that is the best fit for your research. If you are not sure, read the appropriate sections before making your selection.

☐ Life sciences ☒ Behavioural & social sciences ☐ Ecological, evolutionary & environmental sciences

For a reference copy of the document with all sections, see nature.com/documents/nr-reporting-summary-flat.pdf

Behavioural & social sciences study design

All studies must disclose on these points even when the disclosure is negative.

Study description	This was a quantitative experimental study in which participants completed a reinforcement learning and working memory tasks.
Research sample	Experiment 1: A total of 562 participants were recruited via Prolific in exchange for monetary compensation. All reported normal or corrected vision and no history of psychiatric, neurological, or brain injury, nor color blindness. Of these, 279 participants were assigned to the abstract condition (M age = 32.0, SD = 8.4; 159 male, 119 female, 1 other) and 283 to the story condition (M age = 31.3, SD = 7.9; 147 male, 122 female, 14 other). Participants completed a reinforcement learning task in Session 1 and a working memory change-detection task in Session 2 on the following day. In total, 524 participants completed both sessions.<br>Experiment 2: A total of 198 participants were recruited via Prolific. All met the same inclusion criteria as in Experiment 1. Participants completed the reinforcement learning task in Sessions 1–3 and a working memory change-detection task in Session 4. A total of 178 participants completed all four sessions on consecutive days (M age = 30.9, SD = 8.2; 93 male, 85 female).<br>Experiment 3: A total of 201 Prolific workers entered the study online in exchange for monetary compensation. All participants were aged 18-50 and reported normal or corrected vision and no current or past psychiatric or neurological diagnosis, color blindness, or brain injury. Participants completed a reinforcement learning task (Mean age = 32.5, SD = 8.2; 104 Male, 96 Female, 1 prefer not to say).<br>Experiment 4A: A total of 150 Prolific workers entered the study online in exchange for monetary compensation. All participants were aged 18-50 and reported normal or corrected vision and no current or past psychiatric or neurological diagnosis, color blindness, or brain injury. Participants completed a reinforcement learning task (Mean age = 32.4, SD = 8.2; 94 Male, 55 Female, 1 prefer not to say).<br>Experiment 4B: A total of 201 Prolific workers entered the study online in exchange for monetary compensation. All participants were aged 18-50 and reported normal or corrected vision and no current or past psychiatric or neurological diagnosis, color blindness, or brain injury. Participants completed a reinforcement learning task (Mean age = 32.3, SD = 8.0; 100 Male, 101 Female).

All samples are representative in terms of both Sex and Age.

Sampling strategy

Sample size was predetermined using a preregistered Bayesian precision analysis, aiming for precise parameter estimation. Specifically, we simulated data and estimated the sample size required to obtain a predefined posterior target width for the HDI95% credible interval. A full description of the Bayesian precision analyses can be found in the preregistrations of all experiments shown in this link: https://github.com/shahar-lab/Automatic-value-learning-results-in-counterproductive-human-behavior/tree/main/Preregistrations.

Data collection

Data was collected using an online task that participants completed via Prolific. Participants entered the study from Prolific and were directed to a task webpage hosted on the Pavlovia server. The experiment was implemented using the jsPsych package (v7.1.2).

Data for Experiment 1 - story condition was collected in August 2024, data for Experiment 1 - abstract condition was collected in February 2025. Data for Experiment 2 was collected in May 2025. Data for Experiments 3 and 4 was collected in November 2025.

We also reanalyzed an existing dataset where 50 adults completed a decision-making task in an fMRI scanner. Participants were between 18 and 30 years old and were recruited from the University of Zurich's Registration Center for Study Participants. Full details can be found in this link: https://www.nature.com/articles/s41562-023-01573-1

Timing

In Experiment 1, participants completed two sessions over two consecutive days. In Experiment 2, participants completed four sessions over four consecutive days.

Data exclusions

We omitted participants and trials from task analyses based on preregistered exclusion criteria. Specifically, we excluded participants who did not complete all sessions (15 in Experiment 2). Next, the first trial of each block, trials with no response, and trials with implausibly fast reaction times (<300 ms) or unusually slow reaction times (>4000 ms). This resulted in the omission of 7.3% of trials in the abstract sample and 5.2% in the story sample in Experiment 1, 7.8% of trials in Experiment 2, 5.8% of trials in Experiment 3, 5.3% of trials in Experiment 4A, and 6.7% of trials in Experiment 4B. Participants were subsequently excluded from task analyses for the following reasons: (a) participants with more than 20% of trials omitted due to no response or implausibly fast reaction times were excluded from further analysis (Experiment 1: 26 participants in the abstract condition and 23 in the story condition; Experiment 2: 22 participants; Experiment 3: 6 participants; Experiment 4A: 14 participants; Experiment 4B: 11 participants); (b) participants who repeated the same key response or switched responses on more than 70% of trials were excluded (Experiment 1: 3 participants in the abstract condition and 6 in the story condition; Experiment 2: 3 participants; Experiment 3: non-applicable; Experiment 4A 2 participants; Experiment 4B: non-applicable); and (c) participants who showed evidence of switching to other browser windows based on mouse-tracking data (5 participants in Experiment 4A). As a result, the final sample in Experiment 1 included 250 participants in the abstract condition and 254 in the story condition, Experiment 2 included 158 participants, Experiment 3 included 195 participants, Experiment 4A included 129 participants, and Experiment 4B included 190 participants.

Non-participation

Experiment 1: A total of 562 participants were recruited via Prolific, and 524 participants completed both sessions. Experiment 2: A total of 198 participants were recruited via Prolific, and 178 participants completed all four sessions on consecutive days. Reasons for dropping between sessions was not provided.

Randomization

In experiment 1, participants were randomly allocated into groups by having each group register into a separate Prolific study. Participants were unable to register to both experiments. The researchers were not blinded to the experimental condition during analyses.

Reporting for specific materials, systems and methods

We require information from authors about some types of materials, experimental systems and methods used in many studies. Here, indicate whether each material, system or method listed is relevant to your study. If you are not sure if a list item applies to your research, read the appropriate section before selecting a response.

Materials & experimental systems

n/a	Involved in the study
☒	☐ Antibodies
☒	☐ Eukaryotic cell lines
☒	☐ Palaeontology and archaeology
☒	☐ Animals and other organisms
☒	☐ Clinical data
☒	☐ Dual use research of concern
☒	☐ Plants

Methods

n/a	Involved in the study
☒	☐ ChIP-seq
☒	☐ Flow cytometry
☒	☐ MRI-based neuroimaging

Plants

Seed stocks	*Report on the source of all seed stocks or other plant material used. If applicable, state the seed stock centre and catalogue number. If plant specimens were collected from the field, describe the collection location, date and sampling procedures.*
Novel plant genotypes	*Describe the methods by which all novel plant genotypes were produced. This includes those generated by transgenic approaches, gene editing, chemical/radiation-based mutagenesis and hybridization. For transgenic lines, describe the transformation method, the number of independent lines analyzed and the generation upon which experiments were performed. For gene-edited lines, describe the editor used, the endogenous sequence targeted for editing, the targeting guide RNA sequence (if applicable) and how the editor was applied.*
Authentication	*Describe any authentication procedures for each seed stock used or novel genotype generated. Describe any experiments used to assess the effect of a mutation and, where applicable, how potential secondary effects (e.g. second site T-DNA insertions, mosiacism, off-target gene editing) were examined.*
