# Supplementary material for: Automatic value learning results in counterproductive human behavior
Source: Nat Commun. 2026 Aug 10;17:9567. doi: 10.1038/s41467-026-76297-7 (PMC13550625; doi:10.1038/s41467-026-76297-7)
Supplement: Supplementary file 3 [file 41467_2026_76297_MOESM3_ESM.pdf]

nature portfolio

Peer Review File

Automatic value learning results in counterproductive human behavior

Corresponding Author: Mr Ido Ben-Artzi

This file contains all reviewer reports in order by version, followed by all author rebuttals in order by version.

Version 0:

Reviewer comments:

Reviewer #1

(Remarks to the Author)
In this article, Ben-Artzi et al. investigate the influence of automatic value learning on choice behaviour. Across two large pre-registered studies using, they find that participants assign value to irrelevant stimulus features - specifically, the side on which a stimulus was presented – even though there was no predictive relationship with the reward. This behavior was very persistent: Even when participants were explicitly told which stimulus features were outcome-relevant, or when they practiced the task extensively, this automatic mechanism could still be observed, despite leading to suboptimal behavior. The authors also found a relationship with working memory capacity.

Overall, I would like to applaud the authors for collecting such large datasets in combination with the reanalysis of exiting data, and for preregistering their hypotheses. The work is clearly rigorous. It is also very interesting to see the persistence of the automatic outcome-irrelevant behavioral effects despite explicit instructions and over multiple training sessions.

However, I do have concerns regarding the interpretation of the results. In my view, what is depicted as “outcome-irrelevant learning” is perhaps more parsimoniously explained by well-documented Pavlovian influences on choice behavior. In addition, the relationship with WM – rather than reflecting a true mechanistic relationship – might perhaps be best explained by general task adherence or attentional engagement. I elaborate on these points in more detail below.

Major comments:

1) My main criticism of this paper is that the irrelevant dimension was always space. There is strong evidence that space has a special significance for humans (and other animals). This raises the question whether what the authors observe should really be interpreted as outcome-irrelevant learning (implying domain-generality) or might instead reflect “learning of the irrelevant spatial dimension”, which has less broad implications for our understanding of RL mechanisms more generally. To really distinguish between these two possibilities, the authors should have manipulated either of the stimulus dimensions as outcome relevant versus outcome irrelevant. I try to avoid suggesting the collection of new datasets, but since the effort is limited in an online setting I wonder whether it might be warranted in this particular setting to run a control experiment that really distinguishes between the two hypotheses?

2) On a related note, if I understand the task description correctly, reward feedback was presented next to the chosen side. Could the observed outcome-irrelevant learning simply reflect Pavlovian approach/avoid biases, i.e. a conditioned association between the chosen spatial location and the outcome, rather than model-free updating of irrelevant stimulus features? Pavlovian learning is highly sensitive to spatial and temporal contiguity. This matters, because the observed effect might not be about a failure of selective credit assignment, but about an additional Pavlovian mechanism influencing choice. If this were true, then mechanistic claims (“model-free RL is automatically updating irrelevant features”) are overstated. Again, an additional experimental manipulation would have been useful. For example, if the effects persist in a variant of the task where the coin is presented randomly next to the chosen or the unchosen object, then the conclusion about irrelevant feature learning would seem more plausible.

3) The authors state that “the role of automaticity in human reinforcement learning has been understudied and remains poorly understood”, but a few papers addressing this question come to mind that the authors must have overlooked. For example, both Jocham et al. (Neuron 2016) and Klein-Flügge et al. (Nature Communications, 2019) demonstrate that

learning mechanisms respecting causal structure and those relying on temporal contiguity are dissociable both neurally and behaviorally. In addition – assuming that point 2 raised above is indeed valid - , there is of course substantial literature on Pavlovian-instrumental transfer showing how automatically acquired Pavlovian values can bias instrumental learning. I suggest the authors rework their framing to clarify what is novel relative to this existing literature.

4) The connection with working memory capacity is interpreted here as protecting against outcome-irrelevant learning. But in the paradigm, only a handful of colors were used – surely, remembering which color is relevant is trivial, even for participants with lower WM capacity? Instead of a mechanistic relationship, I wonder whether WM variance reflects more general factors such as task engagement/adherence: Participants with lower WM scores may simply be noisier, more careless or less attentive. Can the authors provide evidence that the relationship between WM and outcome-irrelevant learning is causal rather than an artifact of compliance? (see also Zorowitz et al., Nature Human Behavior, 2023 on the creation of illusory correlations between behavioral measures and symptom scores through inattentive or low-effort responding).

5) The main conclusion of the paper centers around the fact that λ is smaller than 0. However, the parameter is capped at 1. A leftward-shift could arise simply from boundary-induced truncation rather than reflecting genuine contribution of outcome-irrelevant information to choice. I guess in combination with some of the other (model-free) evidence this is not a fatal concern, but it does raise the question how much of the claim that people systematically use outcome-irrelevant information for making choices may be an artifact arising from boundary effects.

Minor comments:
1) I got confused by the "visual features". There really is only one feature dimension – color – is that right? If so, I suggest labelling this explicitly to avoid confusion, e.g. replace "visual features" by "color": "Accordingly, we defined learning based on the visual features as "outcome-relevant learning,"…"
2) The authors argue that "it might be hard for the human cognitive system to avoid automatically assigning credit in a model-free manner to outcome-irrelevant action features". I wonder whether there is actually an adaptive function to this. For example, in volatile environments the task-relevant stimulus dimensions might change regularly. In such a situation it might make sense to keep track of seemingly irrelevant stimulus-outcome associations (see also latent learning).

(Remarks on code availability)

Reviewer #2

(Remarks to the Author)
Ben-Artzi and colleagues examine behavior in two large online samples of participants playing a multi-armed bandit task. They also reanalyze previously published data from a two-step decision task. In all data sets, they find that participants are less likely to choose a previously unrewarded location/response on the next trial even though they are clearly instructed and report understanding that locations/responses are irrelevant for determining payoffs. They fit a set of RL models to the data and conclude that participants are employing model-free learning that automatically assigns credit to irrelevant location/response features of the task. The model fits are done with state-of-the art methods and the authors thoroughly test for parameter and model recovery. Despite these commendable methodological features, I think the conclusions drawn from the results are fundamentally flawed. There is a bias against choosing a previously non-rewarded location/response, but the data cannot show that this bias is caused by model-free credit assignment and I am doubtful the data can convincingly show that the bias originates from any form of reward learning. My concerns and critiques are listed in detail below.

1. Model-free and model-based behavior cannot be distinguished in the current bandit task. There may be automatic/mistaken credit assignment to locations or button presses, but there is no way to know which system or combination of systems is assigning that credit. Narrative instructions and correct answers to comprehension questions cannot fully guarantee correct model understanding or consistent correct model use throughout the task. For example, participants may start to use inaccurate simplifying heuristics or strategies during the task because of boredom, frustration, or a desire to save effort.

2. I don't follow the logic of the interpretations of the data from the extensive training experiment. The authors suggest that this is evidence of an automaticity that does not go away with experience. As the authors point out, this is opposite of what one would expect from a reward learning system, which should learn that locations/responses are unpredictive of reward. This raises a further question about why we should think that automatic/mistaken credit assignment to locations/responses is part of any reward learning system (model-based or model-free). Please provide some reasons as to why you claim that the extensive training results support the idea that reward learning systems automatically assign credit to locations/responses, rather than contradicting the idea that such responses are learned.

3. The parameter fits from the full RL model indicate that decisions are based largely on color (lambda is close to 1 for most participants). Given the strong weighting in favor of color in the calculation of net Q-values, location will only be able to shift the choice from the option favored by color when the difference in color Q-values is very small. In other words, when it is unclear which color is better. It seems plausible that a deliberative decision-maker (i.e. model-based) might break approximate ties with some known irrelevant feature or heuristic rule simply to avoid spending time and effort to pick between two nearly equal options. This effort saving strategy may be more prominent in participants with low working memory ability who may find deciding between two similar options more effortful (or low working memory and low lambdas

may both stem from low motivation to do either task well). Given the inability to distinguish model-based and model-free behavior in this bandit task, is there any way to rule out the possibility that participants use such a tie-breaking heuristic?

4. Do lower values of lambda lead to lower payoffs in addition to lower accuracy? The bandit payoffs are probabilistic so making the technically correct response will not always yield reward, and conversely, the incorrect response is sometimes rewarded. Participants don't get direct feedback on being correct or incorrect, they only observe a reward or not. Payoffs on tasks with drifting probabilistic rewards are famously insensitive to the use of optimal versus (somewhat) suboptimal strategies (e.g., Kool et al 2016 https://doi.org/10.1371/journal.pcbi.1005090).

5. Can we distinguish mistaken credit assignment to location/response from responding randomly on a subset of trials? The locations are not predictive of reward and the learning rates listed in Table 1 are modest (~ 0.3). Therefore, if the locations are assigned credit, the Q values learned for each location should be very similar on most trials. The contribution of the location Q values to making a left choice is Q_L/(Q_L + Q_R) or ~ ½, if Q_L and Q_R are similar. So, another plausible alternative model is one in which lambda is a measure of a tendency for random responding instead of mistaken credit assignment. Does the full model (with lambda scaling color vs location influence) fit better than this alternative with lambda scaling color's influence relative to a random choice)? The fact that a substantial minority of participants have lambdas below 0.5 in Fig. 1D makes me think that this parameter is not only/primarily capturing the influence of a mistaken/automatic tendency to use locations in forming net Q-values. Relying on unpredictive location/responses *more* than the predictive colors seems unlikely for any RL system in healthy adults.

6. Related to the idea that lambda may be an additional measure of randomness beyond the inverse temperature parameter, what is the correlation between the two parameters? Do they trade off against each other? The authors state that they inspected pairs plots for the fitted parameters. Could you please add those to the supplementary material so that readers can see the correlations between lambda and all other estimated model parameters?

7. Does the full RL model fit the data better than a simple categorical regression model? The current paper reuses data from Feher and colleagues 2023, which reported that a simple categorical regression model fit their data better than RL models did. Those authors claim that this result indicates that participants' true strategies deviate substantially from the learning approach(es) specified in the RL model. A similar sanity check on the appropriateness of the RL model specification for the bandit task data would be helpful to include.

Minor points
8. Why constrain the learning rate to be equal for relevant and (automatic) irrelevant features?

9. The authors write on line 753 that, "Positive values of rho reflected a bias toward repeating previously chosen features, whereas negative values captured a tendency to explore alternatives." This is not strictly true when combining equations 5 and 6. Instead, the value of both rho and eta determine the bias toward repeating previously chosen features. If both have the same sign, then there is a bias to repeat, but if they have opposite signs, then there is a bias against repeating. This is important because Table 1 shows negative values for rho_spatial and eta_spatial, and if looking at rho alone readers might mistake it for a bias against repeating spatial locations.

(Remarks on code availability)

Version 1:

Reviewer comments:

Reviewer #1

(Remarks to the Author)
The authors did an impressive job in addressing my concerns. They collected additional data where the original datasets were insufficient for addressing some of the issues I raised, they added a set of new analyses that specifically address the outstanding points and revised their manuscript accordingly. In my view, the new results substantially strengthen the evidence supporting the main claims. I am happy to now recommend publication in Nature Communications.

One minor point: I would suggest standardising the y-axis limits to facilitate comparison of effect sizes across stimulus dimensions, e.g. in Fig. 4B and 4D or Fig. 5D-G.

(Remarks on code availability)

Reviewer #2

(Remarks to the Author)
In my opinion the authors have done a thorough and convincing job of addressing/removing the concerns raised about the

previous version of this manuscript. This was a good deal of work, I'm sure, but the evidence from the new experiments and analyses makes the paper much stronger now. I also appreciate the author's willingness to clarify some of the conceptual framing and claims in the paper. In their reply to my comment about the labeling of outcome-irrelevant learning as model-free and the corresponding implicit claim that the outcome-irrelevant learning is limited to a model-free system they stated that agreed with the point and,

"We now only refer to “model-free” in a dedicated discussion paragraph that addresses the nuances around model-free processing, as can be seen below. Critically, we assert that demonstrating
automatic assignment of value to outcome-irrelevant action features is a meaningful contribution to the field of human learning and decision making, regardless of whether outcome-irrelevant learning is predominantly model-free or model-based. "

I think this discussion paragraph is important and I agree with the assertion that contribution is meaningful regardless of where outcome-irrelevant learning comes from. That said, the label 'Outcome-irrelevant model-free learning' still appears 5 times in the revised manuscript. I assume this is an oversight on the authors' part given their statement in the rebuttal letter.

In addition to the 5 remaining labels the following sentence from the paper directly claims that a model-free system is responsible for the outcome-irrelevant learning.

"This finding supports the conclusion that outcome-irrelevant
learning reflects an inherent component of human model-free reinforcement learning, rather than
a misunderstanding of the task structure."

As we seem to agree that the data cannot establish if outcome-irrelevant learning is model-based/free or something else altogether, I ask the authors to revise the sentence to read,

"This finding supports the conclusion that outcome-irrelevant
learning reflects an inherent component of human reinforcement learning, rather than
a misunderstanding of the task structure."

To be clear, it is still very important to show that outcome-irrelevant learning is not driven (solely) by confusion about the task structure. I simply want to push the field in general away from "automatically" lumping sub-optimal behaviors onto a model-free system without clear evidence for its involvement.

Other than these minor modifications to the text, I think the current version of the manuscript makes important contributions to the field.

(Remarks on code availability)

Open Access This Peer Review File is licensed under a Creative Commons Attribution 4.0 International License, which permits use, sharing, adaptation, distribution and reproduction in any medium or format, as long as you give appropriate

credit to the original author(s) and the source, provide a link to the Creative Commons license, and indicate if changes were made.
In cases where reviewers are anonymous, credit should be given to 'Anonymous Referee' and the source.
The images or other third party material in this Peer Review File are included in the article's Creative Commons license, unless indicated otherwise in a credit line to the material. If material is not included in the article's Creative Commons license and your intended use is not permitted by statutory regulation or exceeds the permitted use, you will need to obtain permission directly from the copyright holder.
To view a copy of this license, visit https://creativecommons.org/licenses/by/4.0/

Reviewer #1:

In this article, Ben-Artzi et al. investigate the influence of automatic value learning on choice behaviour. Across two large pre-registered studies using, they find that participants assign value to irrelevant stimulus features - specifically, the side on which a stimulus was presented - even though there was no predictive relationship with the reward. This behavior was very persistent: Even when participants were explicitly told which stimulus features were outcome-relevant, or when they practiced the task extensively, this automatic mechanism could still be observed, despite leading to suboptimal behavior. The authors also found a relationship with working memory capacity.

Overall, I would like to applaud the authors for collecting such large datasets in combination with the reanalysis of exiting data, and for preregistering their hypotheses. The work is clearly rigorous. It is also very interesting to see the persistence of the automatic outcome-irrelevant behavioral effects despite explicit instructions and over multiple training sessions.

However, I do have concerns regarding the interpretation of the results. In my view, what is depicted as "outcome-irrelevant learning" is perhaps more parsimoniously explained by well-documented Pavlovian influences on choice behavior. In addition, the relationship with WM - rather than reflecting a true mechanistic relationship - might perhaps be best explained by general task adherence or attentional engagement. I elaborate on these points in more detail below.

Thank you for the positive and constructive evaluation and for these insightful comments. We undertook a substantial revision to address your suggestions, including the completion of three additional experiments (N = 195 in Experiment 3; and N = 129 / 190 in Experiments 4A / 4B). We trust that this addresses your concerns in full and provide a point-by-point reply below.

Major comments:

1) My main criticism of this paper is that the irrelevant dimension was always space. There is strong evidence that space has a special significance for humans (and other animals). This raises the question whether what the authors observe should really be interpreted as outcome-irrelevant learning (implying domain-generality) or might instead reflect "learning of the irrelevant spatial dimension", which has less broad implications for our understanding of RL mechanisms more generally. To really distinguish between these two possibilities, the authors should have manipulated either of the stimulus dimensions as outcome relevant versus outcome irrelevant. I try to avoid suggesting the collection of new datasets, but since the effort is limited in an online setting I wonder whether it might be warranted in this particular setting to run a control experiment that really distinguishes between the two hypotheses?

We followed the reviewer's suggestion and completed an additional preregistered experiment (Experiment 3, N=195), testing the domain-generality of outcome-irrelevant learning. We found clear evidence that outcome-irrelevant learning is not selective to the spatial-motor domain, but is also present when the visual feature (i.e., color) of an action is outcome-irrelevant. Specifically, in Experiments 1 and 2, spatial-motor features (i.e., location/response-key) were outcome-irrelevant, and a visual feature of the stimuli (i.e., color) determined the outcome. We now present in Experiment 3 a modified multi-armed bandit task where the visual feature is outcome-irrelevant, and spatial-motor features determine reward.

Both computational modeling and signature regression analysis clearly showed that despite clear, reliable, and story-like instructions, participants engage in outcome-irrelevant learning in the visual domain. Therefore, we assert that counterproductive outcome-irrelevant learning is not selective to a specific domain (e.g., spatial-motor).

The introduction now includes:

Third, it is important to determine the extent to which outcome-irrelevant learning reflects a domain-general phenomenon. Prior studies demonstrating outcome-irrelevant learning have been largely confined to the spatial-motor domain, raising the question of whether automatic credit assignment also occurs in other domains [13,17,18]. If outcome-irrelevant learning operates automatically, then all action features activated as part of the task set should, in principle, receive credit. During action selection in a reinforcement-learning context, all motor and perceptual features required to execute a choice are activated as part of the task set [53–55]. One possibility is that all features activated during action selection, regardless of domain, are retrospectively attended to at outcome delivery and are therefore assigned credit, even when they are known to be irrelevant for predicting reward [56]. Alternatively, spatial-motor features may hold a privileged status due to their central role in natural environments, making credit assignment to locations or motor actions difficult to suppress even when they are known to be non-predictive of reward [57,58].

The results now include the following section:

Outcome-irrelevant learning extends beyond spatial-motor representations

To test whether outcome-irrelevant learning is specific to spatial-motor representations, we completed a preregistered experiment (N=195) using a modified version of the "story" framing used in Experiments 1&2. Here, the locations were made outcome-relevant while stimulus color was irrelevant to outcome prediction. The task included four available locations (left, middle-left, middle-right, and right). In each trial, participants were offered two of the four locations for their selection. The locations that were offered for selection in a given trial were marked by two “colored leaves” (e.g., orange or blue), while the two locations that were not available for a given trial were marked by two “green leaves” (see Figure 4A). In each trial, the allocation of the four colors (e.g., green, green, orange, blue) to each of the four locations was randomly assigned by the computer. Therefore, participants had to attend to the color during the selection phase to know which locations were offered. Yet, colors had no association with outcomes, which were determined only by the chosen location. After choosing a location, participants observed whether they obtained a reward or not. Each location led to a monetary outcome (unrewarded or rewarded) with a latent expected probability that was unknown to participants and that slowly drifted across trials. Participants were therefore asked to observe the outcomes that followed their selection and try to make decisions that would maximize return. We made sure, using story-based instructions and a dedicated quiz, that participants were well aware that only the location, and not the color of the stimuli, was outcome-relevant.

Confirming our preregistered hypothesis, we found a clear indication that participants assigned credit for the outcome-irrelevant colors. Computational modeling showed that the population-level λ parameter was substantially lower than 1 (posterior median = .59, HDI95% [.38, .78]; see Figure 4B). Our

model-agnostic signature further examined the tendency to re-select a previously selected color as a function of reward. This was examined only in trials where the two previously offered locations were not reoffered for selection in the following trial (see Figure 4A). We fitted a hierarchical logistic Bayesian regression and found that participants were more likely to repeat a previously chosen color following a rewarded (61%) vs. an unrewarded (47%) trial (posterior median = .54, HDI95% [.38, .7], pd =~100%; see Figure 4C). We further estimated the degree to which participants were able to associate rewards with the outcome-relevant locations and found a clear outcome-relevant reward learning signature, such that when the previously chosen location was reoffered, participants were more likely to repeat it following a rewarded vs. an unrewarded trial (posterior median = 1.07, HDI95% [.88, 1.26], pd =~100%; see Figure 4D). This indicates that despite a clear understanding of the task, individuals found it difficult to inhibit assigning credit to task-relevant, yet outcome-irrelevant colors. Thus, we conclude that outcome-irrelevant learning generalizes to other non-spatial-motor domains.

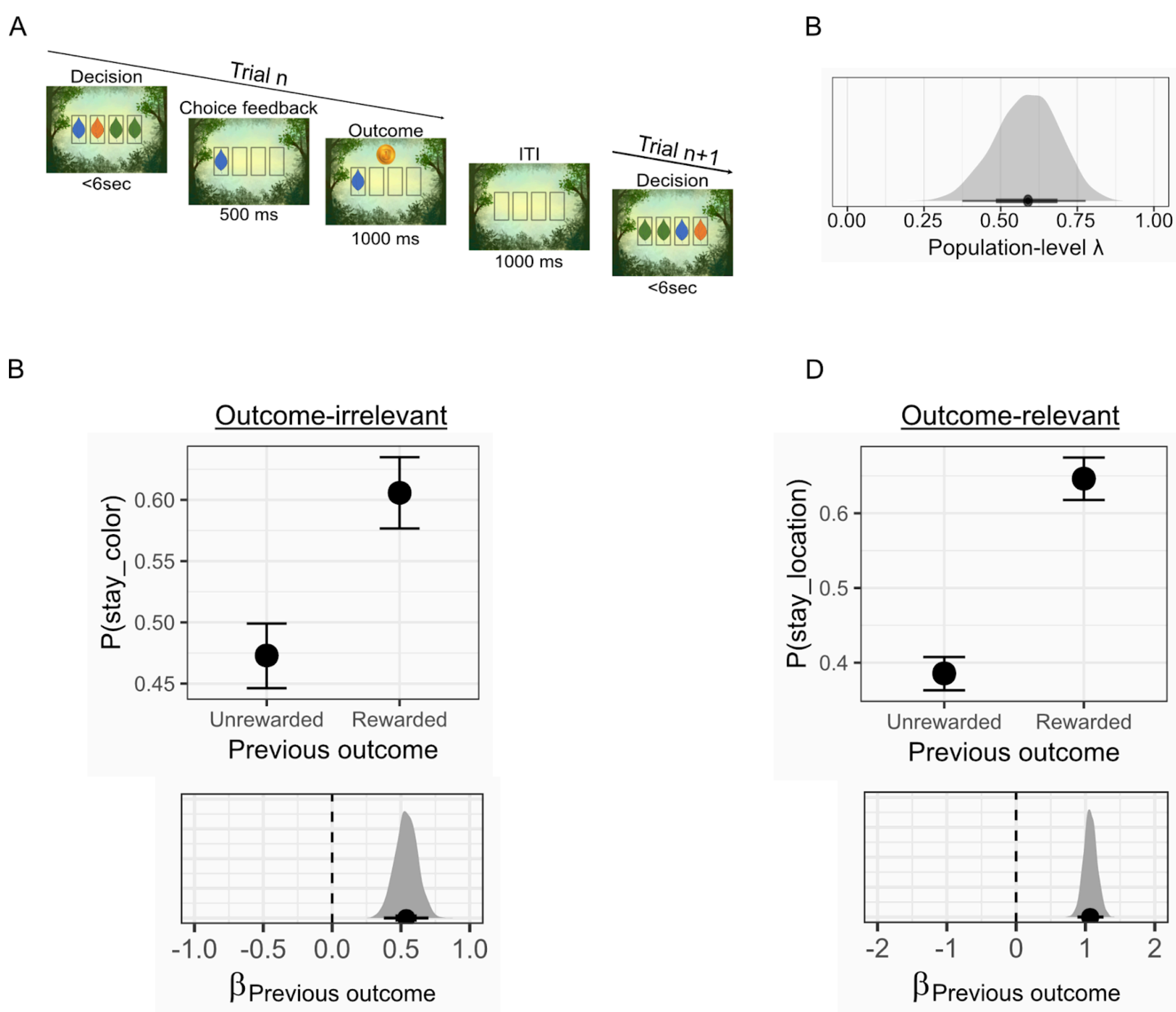

Figure 4. Outcome-irrelevant learning extends beyond spatial-motor representations. (A) To test whether outcome-irrelevant learning reflects a domain-general process or is restricted to spatial-motor representations, we modified the "story" condition from Experiment 1. Specifically, we flipped the

outcome-relevance of the two action features, such that the color was now outcome-irrelevant, and the location/response-key was outcome-relevant. On each trial, two out of four locations were offered for choice. The two offered locations in a given trial were marked by two “colored leaves” (e.g., orange or blue), while the two locations that were not available for a given trial were marked by two “green leaves”. Participants were comprehensively instructed and tested for their understanding that the specific color of the non-green leaf was irrelevant for outcome prediction, and choices should solely depend on the chosen location. **(B)** We fitted the computational model to participants’ behavior in Experiment 3, where colors were outcome-irrelevant, and choices should have been determined solely by the location. Higher values of λ still indicate greater reliance on outcome-relevant information (in this case, locations). We find a clear indication that participants were unable to inhibit assigning credit to outcome-irrelevant colors. **(C)** We evaluate whether the previous outcome affects color selection in trial $n+1$, when the locations from trial n were not reoffered. We find a robust effect indicating that outcome-irrelevant learning generalizes beyond spatial-motor representations. **(D)** To further confirm that individuals were able to follow the values of outcome-relevant locations, we estimated a win-stay lose-shift signature on trials where the previously chosen location was reoffered. We find a clear effect of previous outcome on location choice. The black circle in the figure represents the posterior median, and the horizontal line indicates the HDI95%.

The discussion now includes:

Previous work suggested that interference from outcome-irrelevant features might be limited to spatial-motor representations (Eckstein & Collins, 2020; Rmus et al., 2023). However, in Rmus et al., the outcome-irrelevant visual feature was also irrelevant for executing the task. That is, participants could have made their choice without exerting any attention to the visual feature (color). Thus, in Rmus et al., the lack of interference from the visual feature may be due to it being irrelevant to the whole task, not just the outcome. Here, we designed a task where visual features are necessarily attended during action execution, yet irrelevant for outcome prediction. We found clear evidence that value is assigned to outcome-irrelevant colors. We conclude that credit may be automatically assigned to all action features that are part of the task set, even when these are outcome-irrelevant.

2) On a related note, if I understand the task description correctly, reward feedback was presented next to the chosen side. Could the observed outcome-irrelevant learning simply reflect Pavlovian approach/avoid biases, i.e. a conditioned association between the chosen spatial location and the outcome, rather than model-free updating of irrelevant stimulus features? Pavlovian learning is highly sensitive to spatial and temporal contiguity. This matters, because the observed effect might not be about a failure of selective credit assignment, but about an additional Pavlovian mechanism influencing choice. If this were true, then mechanistic claims (“model-free RL is automatically updating irrelevant features”) are overstated. Again, an additional experimental manipulation would have been useful. For example, if the effects persist in a variant of the task where the coin is presented randomly next to the chosen or the unchosen object, then the conclusion about irrelevant feature learning would seem more plausible.

We highly appreciate the reviewer's justified concern. To address this concern, we have now included two additional pre-registered experiments described below, demonstrating that outcome-irrelevant learning cannot be explained as a Pavlovian learning effect.

Specifically, the reviewer raised a concern that the presentation of the chosen arm in a specific location on screen, along with the outcome at the screen center, could lead to a “choice-free” Pavlovian association between location and outcome. We would like to briefly clarify that in Experiments 1&2, the outcome was always presented at the center of the screen and not at the side of the chosen bandit. We apologize if this was previously unclear in the methods verbal explanation, and have now made sure that this is clearly mentioned in the methods section (see excerpt below). However, a Pavlovian association can still occur during the outcome presentation. As the reviewer noted, it is possible that at the time of outcome presentation, participants generated a Pavlovian association between the side of the chosen arm (which was more perceptually salient than the other side) and the outcome. Such a Pavlovian association can later result in approach/avoid behavior, providing a different mechanism than instrumental learning to the reported results (i.e., Pavlovian to instrumental transfer).

To demonstrate that outcome-irrelevant learning is mainly a choice-based learning effect, we followed the reviewer's recommendation for collecting additional data. We now provide results for two new preregistered experiments, one where the locations/response-keys are outcome-irrelevant (i.e., Experiment 4A; N=129), and one where the colors are outcome-irrelevant (i.e., Experiment 4B; N=190). These are very similar to previous versions of the task (Experiment 1-3). Critically, we made sure that all spatial/visual features gained exactly the same exposure on screen, eliminating any perceptual-Pavlovian association between an outcome and a chosen/unchosen feature (see Figure 5, below). This was achieved by omitting the visual indication of the chosen arm from the choice-feedback and outcome screens, thereby dramatically mitigating the contribution of a Pavlovian to Instrumental transfer to the effect. As can be seen below, we replicate the signature of outcome-irrelevant learning on both domains, with no reduction in the estimated effect of outcome-irrelevant learning compared with Experiments 1 to 3. Based on the results of these experiments, we conclude that outcome-irrelevant learning can not be explained by Pavlovian processes and reflects a choice-based credit assignment process to chosen, yet outcome-irrelevant features.

<u>To improve the explanation of Experiment 1, the methods now include:</u>

After selecting an arm, the chosen arm remained on screen for 0.5 sec without the unchosen arm. Next, a feedback screen appeared where the selected arm remained on screen, and the monetary outcome (either a gold coin in rewarded trials or a black coin in unrewarded trials) appeared at the screen center for 1 sec (see Figure 1 for trial sequence).

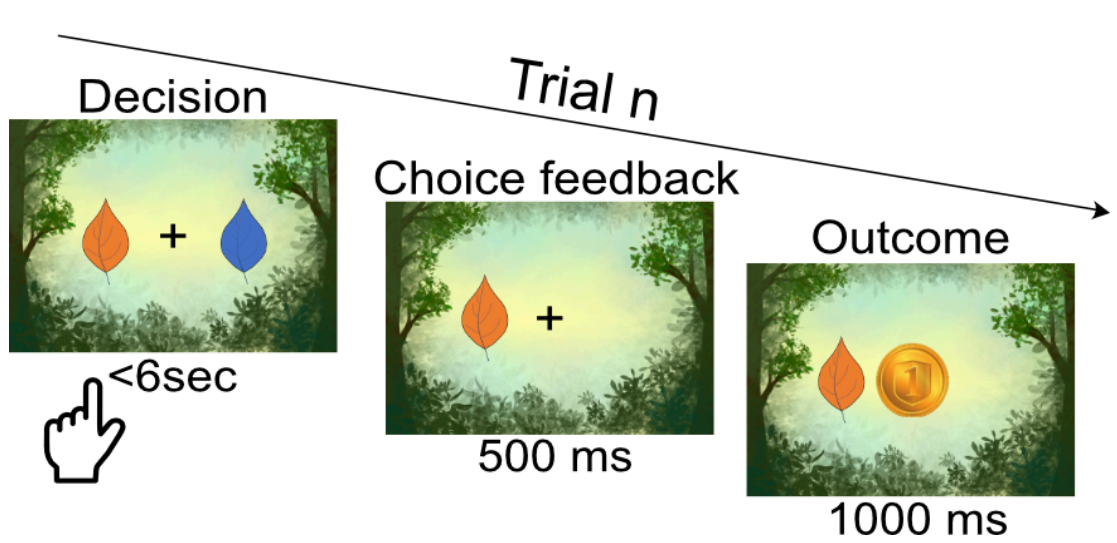

<u>The results now include the following section with two new experiments:</u>

Outcome-irrelevant learning can not be explained by a Pavlovian bias

We performed an additional experiment to minimize the possibility that Pavlovian processes were a main contribution to the previous results (Afshar et al., 2023; Pearce & Hall, 1980; Rescorla & Wagner, 1972). Specifically, in Experiments 1-3, the chosen option remained on the screen during outcome presentation. Thus, a Pavlovian association between the outcome-irrelevant chosen side or chosen color could have been formed. Following a Pavlovian to Instrumental transfer (Estes, 1948; Talmi et al., 2008), this may then lead to a bias towards approaching an outcome-irrelevant feature (i.e., side/color) following a rewarded trial and avoiding it following an unrewarded trial.

To test whether Pavlovian associations that might have formed during outcome presentation are the main driver of our effect, we completed two additional preregistered experiments that dramatically reduce the potential contributions of Pavlovian processes. Experiment 4A included locations and response keys as outcome-irrelevant and color as outcome-relevant (N=129; similar to Experiments 1&2). Experiment 4B included the opposite, namely, color as outcome-irrelevant and locations/response keys as predicting the outcome (N=190; similar to Experiment 3). Critically, in both experiments, we modified the designs so that during both choice feedback and outcome presentation, the chosen location or color was not shown on the screen (see Figure 5A&B). This minimized potential Pavlovian associations between the outcome-irrelevant features and the outcome.

We replicated our results from previous experiments in both domains, showing a reward-dependent change in the tendency to choose a knowingly outcome-irrelevant feature. We estimated the population-level λ parameter using computational modeling in both conditions, and found the λ parameter to be substantially lower than 1 (Experiment 4A: posterior median = .88, HDI95% [.82, .94]; see Figure 5C; Experiment 4B: posterior median = .78, HDI95% [.62, .92], see Figure 5D). To corroborate this conclusion, we replicated our model-agnostic signature showing that outcome-irrelevant locations are more likely to be repeated following a rewarded (50%) vs. an unrewarded trial (44%; Experiment 4A: posterior median = .24, HDI95% [.11, .38], pd = ~100%; see Figure 5E). To further ensure that our manipulation did not interfere with participants' ability to learn the relevant dimension, we also share results indicating a strong win-stay lose-shift signature for the previously chosen color, such that when a previously chosen color was reoffered, participants were more likely to choose it after a rewarded trial (75%), than after an unrewarded trial (42%; Experiment 4A: posterior median = 1.43, HDI95% [.1.23, 1.63], pd ~=100%; see Figure 5F). Similarly, when visual information was outcome-irrelevant (Experiment 4B; see Figure 5B), we find participants are more likely to repeat a previously chosen color following a rewarded (60%) than after an unrewarded trial (48%; Experiment 4B: posterior median = .48, HDI95% [.35, .62], pd = ~100%; see Figure 5G). Participants were again still able to learn the values of the outcome-relevant spatial-motor feature (Experiment 4B: posterior median = 1.14, HDI95% [.97, 1.3], pd = ~100%; see Figure 5H). Thus, we conclude that outcome-irrelevant learning persists even when mitigating the contribution of Pavlovian associations.

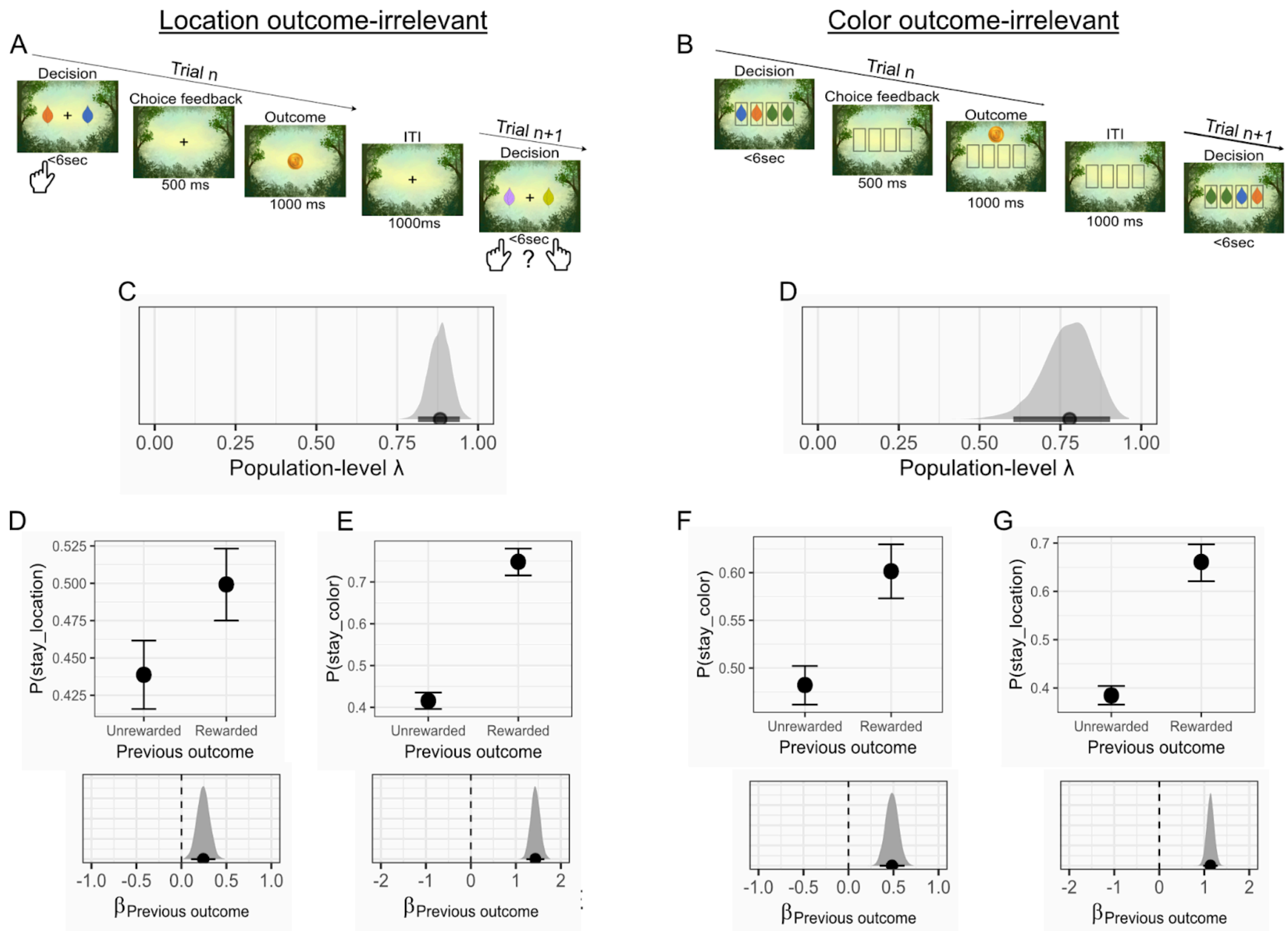

Figure 5. Outcome-irrelevant learning is observed even when choice feedback is omitted. We conducted two additional experiments demonstrating that outcome-irrelevant learning cannot be explained by choice-free Pavlovian association. **(A/B) Trial sequence for Experiments 4A and 4B.** Here, we repeated the tasks used in previous experiments where either location (Exp. 4A) or color (Exp. 4B) was outcome-irrelevant. Critically, we omitted the choice feedback presentation completely. This ensured that all features of the two offers gained exactly the same exposure on screen, preventing any possible Pavlovian association between the outcome and an outcome-irrelevant feature of the chosen arm. **(C/D) Posterior distributions of the λ parameter** estimated separately for each experiment using computational modeling showed a clear indication that even when the two options gained equal presentation on screen, participants were unable to inhibit credit assignment to outcome-irrelevant locations (Exp. 4A) or colors (Exp. 4B). **(D/F) Model-agnostic signature** showing that participants were more likely to stay with the same outcome-irrelevant feature (i.e., location for Exp. 4A; color for Exp. 4B) after a rewarded trial compared to an unrewarded trial (probability of direction=~100%). **(E/H)** We further show the outcome-relevant win-stay lose-shift signature, indicating that participants were clearly able to learn the value of the outcome-relevant feature (colors Exp. 4A; locations Exp. 4B; probability of direction=~100%).

3) The authors state that “the role of automaticity in human reinforcement learning has been understudied and remains poorly understood”, but a few papers addressing this question come to mind that the authors must have overlooked. For example, both Jocham et al. (Neuron 2016) and Klein-Flügge et al. (Nature

Communications, 2019) demonstrate that learning mechanisms respecting causal structure and those relying on temporal contiguity are dissociable both neurally and behaviorally.

We thank the reviewer for sharing these important papers. We see it as complementing our claims and now refer to them in the Introduction. Specifically, Jocham et al. (Neuron 2016) tested humans' ability to assign credit to actions preceding the receipt of reward by exactly 3 seconds, finding that humans wrongly temporally assigned credit in a non-specific manner, such that credit spread also to non-causally related choices. Similarly, Klein-Flügge et al. (Nature Communications, 2019) tested humans' ability to assign credit for grid sequences, finding an incidental spread of credit to spatially proximal locations. However, these previous demonstrations of non-causal credit assignment showed a spreading of credit to "irrelevant items" within *the outcome-relevant feature* (i.e., wrong timing, or locations on a grid) (Guttman & Kalish, 1956; Kahnt et al., 2012). A striking aspect of our work is in extending this literature by showing an automatic credit assignment even when there is a clear dimensional separation between relevant and irrelevant information, allowing, in principle, a complete filtering-out of irrelevant information during credit assignment.

The Introduction now includes:

However, in a scenario where the environment's underlying causal structure is not represented in a direct manner, the learning system might struggle to withhold assigning credit to incidental characteristics like the apple's location on the tabletop. Previous work suggested that the human brain assigns credit from rewarding outcomes to incidental and non-causally related representations (Jocham et al., 2016; Klein-Flügge et al., 2019). However, demonstrations of such non-causal credit assignment have been largely confined to credit spreading within the outcome-relevant feature (Guttman & Kalish, 1956; Kahnt et al., 2012), for example, assigning credit to actions that fall outside the causal time window (Jocham et al., 2016) or to spatially adjacent actions despite explicit knowledge of the task's spatial structure (Klein-Flügge et al., 2019). Our work extends this literature by showing, for the first time, an automatic credit assignment even when there is a clear dimensional separation between outcome-relevant and outcome-irrelevant information, allowing, in principle, a complete filtering out of irrelevant information during credit assignment. Thus, we argue that the human value-based learning system automatically forms action-outcome associations between action features and an outcome, even when both prior knowledge and extensive experience indicate that those action features are irrelevant to the outcome, and despite leading to reduced gains.

In addition - assuming that point 2 raised above is indeed valid - , there is of course substantial literature on Pavlovian-instrumental transfer showing how automatically acquired Pavlovian values can bias instrumental learning. I suggest the authors rework their framing to clarify what is novel relative to this existing literature.

We thank the reviewer and have now added a paragraph discussing this claim.

The discussion now includes:

Outcome-irrelevant learning can, in principle, be interpreted through a Pavlovian-to-Instrumental Transfer (PIT) account (Estes, 1948; Talmi et al., 2008). In PIT, a Pavlovian cue that is associated with

reward can later invigorate an instrumental response directed toward that cue. According to this Pavlovian account, the association between a location and a reward is formed independently of one's action. In this view, when an option is chosen, then the outcome-irrelevant feature of the selected arm might become linked to reward simply because the reward co-appears with that feature on screen, rather than having credit being assigned to the individual's choice. Experiments 1-3 may have been susceptible to this explanation, as the outcome-irrelevant features (location in Experiments 1-2, color in Experiment 3) were present on the screen while the outcome was displayed. This temporal overlap may have led the irrelevant feature to acquire a Pavlovian association, which could then bias instrumental responding toward the same side or color in subsequent trials. To address this concern, we designed Experiment 4 so that the outcome-irrelevant features (4A-locations/4B-colors) were displayed symmetrically during choice feedback and outcome presentation, eliminating any differential pairing between reward and an irrelevant feature. We still observed robust outcome-irrelevant learning effects, suggesting PIT is not the sole mechanism driving the outcome-irrelevant learning effect, and that choice-based credit assignment to outcome-irrelevant features persists in shaping behavior.

4) The connection with working memory capacity is interpreted here as protecting against outcome-irrelevant learning. But in the paradigm, only a handful of colors were used - surely, remembering which color is relevant is trivial, even for participants with lower WM capacity? Instead of a mechanistic relationship, I wonder whether WM variance reflects more general factors such as task engagement/adherence: Participants with lower WM scores may simply be noisier, more careless or less attentive. Can the authors provide evidence that the relationship between WM and outcome-irrelevant learning is causal rather than an artifact of compliance? (see also Zorowitz et al., Nature Human Behavior, 2023 on the creation of illusory correlations between behavioral measures and symptom scores through inattentive or low-effort responding).

We agree that a main concern in claiming that humans automatically assign credit to outcome-irrelevant features is whether this outcome-irrelevant learning may solely be an artifact of disengagement. However, the results of the current experiments clearly indicate that outcome-irrelevant learning is a property of the engaged human cognitive system that is not explained by task inattentiveness, as supported by the following analyses.

First, our computational model includes a β parameter, which captures random reward-insensitive behavior typically shown by participants who are inattentive to the task. The β parameter is recoverable and is clearly distinct from the λ parameter capturing the tendency for outcome-irrelevant learning. Importantly, the model-agnostic signature we described in the manuscript is negatively associated with λ (see Figure 7A) but positively associated with the β parameter (see Figure 7B). That is, participants who showed a *stronger* outcome-irrelevant stay behavior also had *higher* β parameter estimates. This is to be expected, as the model-agnostic outcome-irrelevant learning signature reflects a reward-driven behavior that can not arise from the behavior of a random responder. To further substantiate this point, we provide a simulation where artificial agents are simulated with ($\beta>0$, $\lambda=1$), but had in one third of the trials an "attentional lapse" causing them to respond randomly with respect to reward (i.e., in lapse trials $\beta=0$). We show that such agents have no outcome-irrelevant learning model-agnostic effect (see Figure 7C).

Second, to further strengthen our conclusion that working memory is associated with outcome-irrelevant learning due to its role in reducing the processing of irrelevant information (Dreisbach & Haider, 2008), we provide an additional analysis. Specifically, we fitted a multiple linear regression predicting each individual's WM by λ (indicating outcome-irrelevant learning), β (which is a direct measure of random responding; Wilson & Collins, 2019), and the overall choice accuracy in the bandit task, showing that the association between WM and λ persists (see Figure 7D). The role we suggest for WM in the current task is for holding an activated task set during choice, supporting the inhibition of credit assignment to task-relevant, but outcome-irrelevant representations.

Finally, while we are confident that the relationship between WM and outcome-irrelevant learning is not merely an artifact of task compliance, we can not determine causality and only point to an association. We now extend the relevant paragraph in the limitations section, and made an effort to make it clear that this evidence does not allow for inferring causality.

The results now include:

Inattentive responding does not lead to outcome-irrelevant learning.

Across our work, we reported a reward-driven stay/switch behavioral effect in the outcome-irrelevant dimension (see Figures 2, 4, and 5), which is tightly associated with the λ parameter (Figure 2). We thus interpreted this behavioral effect as a direct, model-agnostic signature of outcome-irrelevant learning. Here, we further aimed to show that this signature effect cannot arise from random responding that can arise due to task inattention.

We addressed this question using three independent analyses. First, we simulated agents using empirically derived parameter values (i.e., learning rate, color-perseveration, and location-switching tendencies). We, however, eliminated outcome-irrelevant learning in those agents by fixing λ to 1. We further introduced attentional lapses in one third of the trials for all agents by temporarily setting $\beta = 0$, resulting in value-independent choices on those trials. Nevertheless, we were able to recover λ as equal to 1 (see SI). Importantly, an in-silico analysis revealed no evidence for a reward-driven stay/switch behavioral effect in the outcome-irrelevant dimension (see Figure 7C). This demonstrates that arbitrary random responding alone cannot produce the empirical behavioral signature we associate with outcome-irrelevant learning.

Second, across Experiments 1&2, we fitted a multiple linear regression predicting each individual's reward-driven stay/switch behavioral effect in the outcome-irrelevant dimension (see Figures 2, 4, and 5) using both their λ and β estimates. Convincingly, we found that a one-unit reduction in β is associated with a 3% decrease in the aforementioned effect (posterior median = 3%, HDI95% [1%, 5%], pd=~100%; see Figure 7B). Therefore, if anything, random responding is associated with a lower signature effect in the outcome-irrelevant dimension. In contrast, λ values were again found to be strongly associated with the model-agnostic signature (posterior median = -.53, HDI95% [-.6, -.45], pd=~100%; see Figure 7A). This asserts that the reward-driven stay/switch behavioral effect in the outcome-irrelevant dimension reflects a behavior that can not arise from a random responder.

Finally, to further illustrate our point that random responding can not give rise to the aforementioned results, we fitted a linear multiple regression analysis predicting working memory capacity by λ while controlling for inattentive/random responding. We find that even when controlling for β and the general choice accuracy in the bandit task, there is a clear association between working memory capacity and the λ parameter (posterior median = 1.32, HDI95% [.92, 1.73], pd=~100%; see Figure 7D).

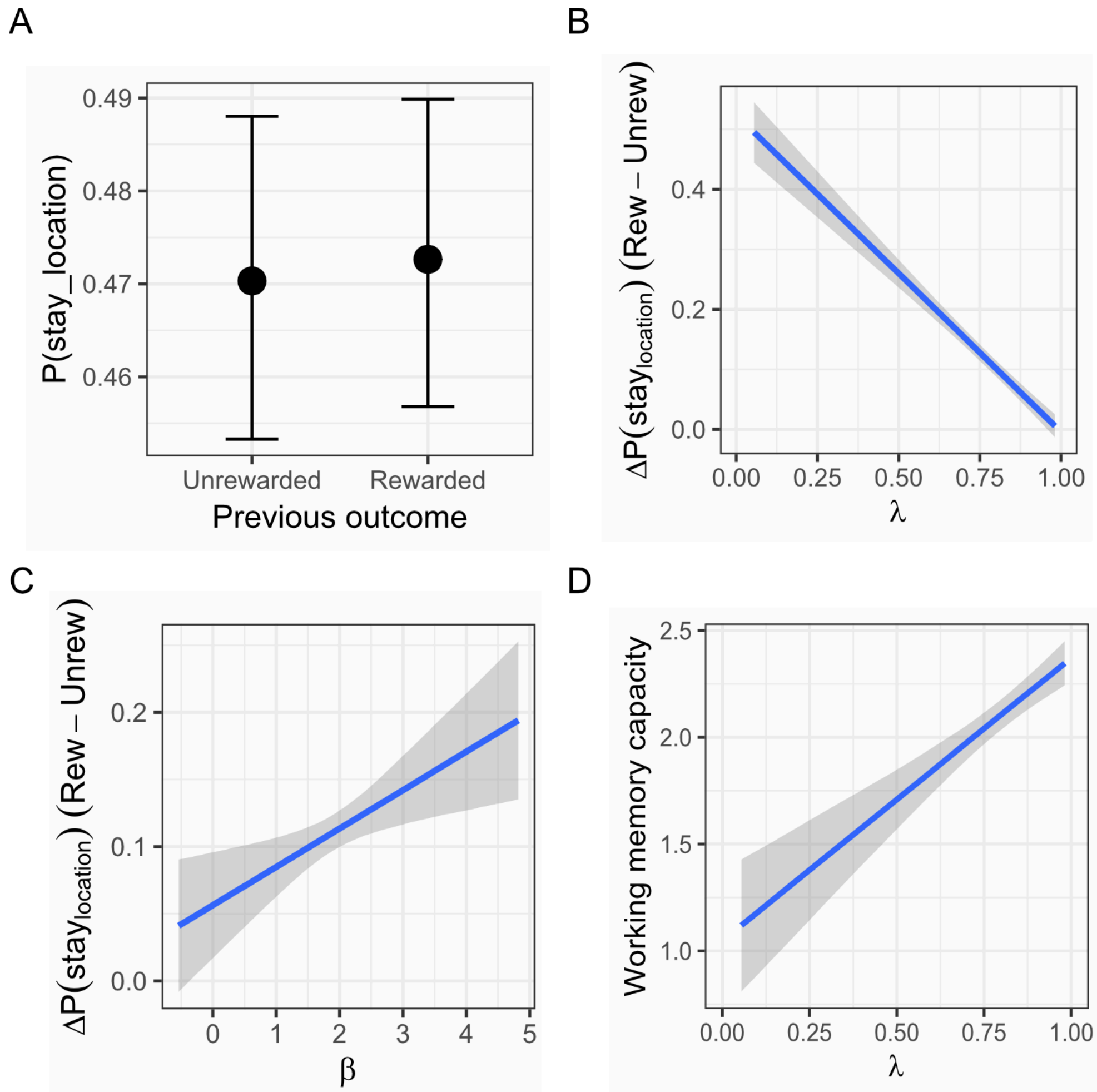

Figure 7. Inattentive responding does not lead to outcome-irrelevant learning. (A) To ensure that the reward-driven stay/switch behavioral effect in the outcome-irrelevant dimension is not driven by random responding, we simulated agents with empirical parameters but with no influence of outcome-irrelevant learning on decision (i.e., λ=1). To explore the possibility that outcome-relevant learning combined with inattentiveness gives rise to outcome-irrelevant learning, on one third of the trials, we included a "lapse of attention", where β is temporarily set to 0. We find that these agents clearly do not show the model-agnostic effect. Next, we predicted for each individual the model-agnostic signature based on both λ and β parameters. Despite λ and β being positively correlated among individuals in our sample, the model-agnostic signature is differentially associated with these parameters. **(B)** While higher degrees of λ are associated with a smaller outcome-irrelevant learning signature, **(C)** higher degrees of β are associated with a greater outcome-irrelevant learning signature. This is to be expected, given that random responding can not generate the effect. **(D)** Finally, we show that the association between λ and working memory is

robust even when controlling for both β and general choice accuracy in the bandit task. That is, the association is not due to inattentive responding in both tasks.

The limitations now include:

Moreover, while we demonstrate that working memory capacity moderates outcome-irrelevant learning, it is yet unknown whether working memory plays a mechanistic role in this process [126]. One option is that by actively maintaining knowledge about outcome-relevance, working memory resources are essential for such regulation [127]. But given the well-established association between working memory and general intelligence [128], research should explore whether the association we found reflects broader aspects of intelligence and cognitive control (Kyllonen & Christal, 1990; Unsworth et al., 2014). To discern whether the association between working memory and outcome-irrelevant learning represents a causal mechanism, future experiments should carefully manipulate working memory resources to assert that this relationship is causal.

5) The main conclusion of the paper centers around the fact that λ is smaller than 0. However, the parameter is capped at 1. A leftward-shift could arise simply from boundary-induced truncation rather than reflecting genuine contribution of outcome-irrelevant information to choice. I guess in combination with some of the other (model-free) evidence this is not a fatal concern, but it does raise the question how much of the claim that people systematically use outcome-irrelevant information for making choices may be an artifact arising from boundary effects.

We find that when λ is simulated as 1, our model fitting procedure confidently estimates it as extremely close to 1 (posterior median = .997; see Figure S6), indicating that capping effects are negligible. Moreover, our model comparison result shows that the full model is better at explaining empirical out-of-sample choices when compared to the reduced model. As the reviewer noted, agents simulated with λ of 1 do not show the model-agnostic signature described in the data (see Figure 7 above). Therefore, we can confidently identify behavior indicating no outcome-irrelevant learning, and we are confident that our findings are not driven by a statistical artifact.

The SI now includes:

The estimation of λ as lower than 1 is not due to statistical capping artifacts

To illustrate that our model fitting procedure can identify data containing no outcome-irrelevant learning, we simulated data of 50 agents from the reduced model, where λ is 1 for all agents, using the design from Experiment 1. We find that our model fitting procedure confidently recovered population-level λ as 1 (posterior median = .997), indicating that capping effects of λ are negligible.

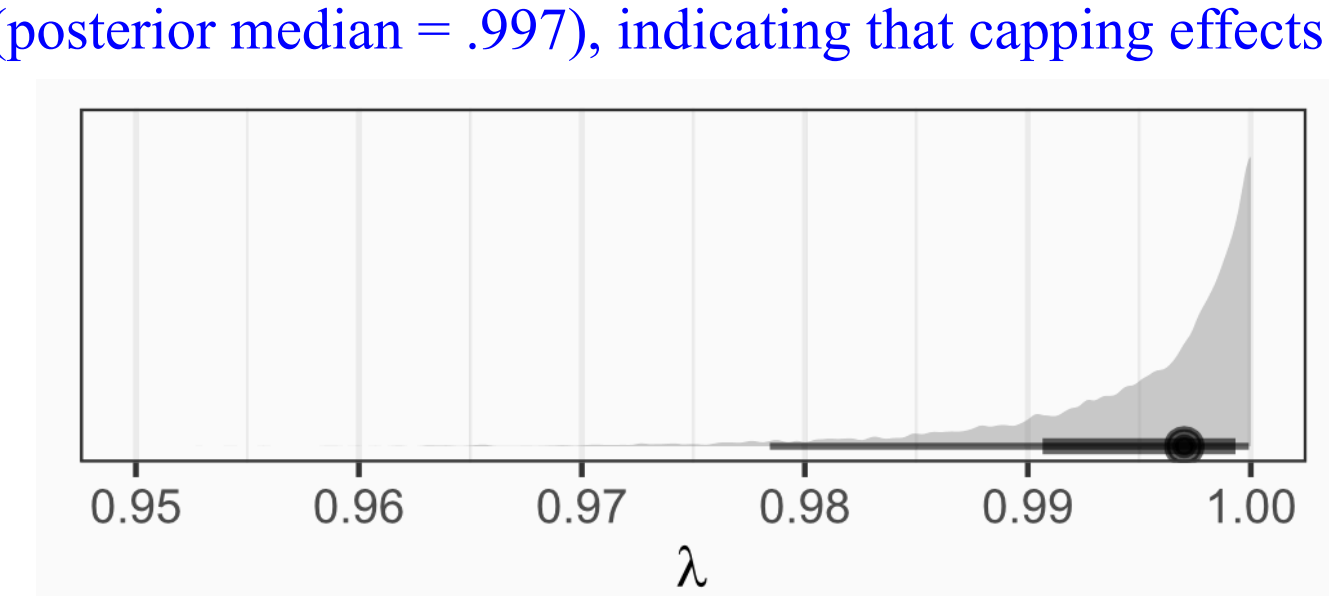

Figure S6. **Recovery of population-level λ = 1.** We simulated 50 agents with λ = 1 and estimated the posterior population-level λ. We found that λ can be clearly recovered (posterior median = .997).

Minor comments:

1) I got confused by the "visual features". There really is only one feature dimension - color - is that right? If so, I suggest labelling this explicitly to avoid confusion, e.g. replace "visual features" by "color": "Accordingly, we defined learning based on the visual features as "outcome-relevant learning,"…"

Thank you. We have now revised the text accordingly.

2) The authors argue that "it might be hard for the human cognitive system to avoid automatically assigning credit in a model-free manner to outcome-irrelevant action features". I wonder whether there is actually an adaptive function to this. For example, in volatile environments the task-relevant stimulus dimensions might change regularly. In such a situation it might make sense to keep track of seemingly irrelevant stimulus-outcome associations (see also latent learning).

Thank you. We agree that this might be a plausible explanation, and now extend the discussion paragraph regarding this.

The discussion now includes:

In contrast, merely tracking the value of an outcome-irrelevant feature might be beneficial by enabling early detection of changes in a volatile environment. For example, consider someone learning to pick apples from a tree while correctly ignoring leaf shape, which is initially unrelated to taste. If a pathogen later makes leaf shape predictive of taste, having encoded the value of leaves would allow the learner to detect this emerging correlation, something that would not be possible if those values had been entirely ignored. Since real-world scenarios are rarely static or guided by explicit instructions, outcome-irrelevant learning may offer greater flexibility in typical dynamic environments. The regulation failure we document may thus reflect the background operation of a change-detection strategy, which carries the cost of allowing irrelevant values to occasionally contaminate choice.

Reviewer #2:

Ben-Artzi and colleagues examine behavior in two large online samples of participants playing a multi-armed bandit task. They also reanalyze previously published data from a two-step decision task. In all data sets, they find that participants are less likely to choose a previously unrewarded location/response on the next trial even though they are clearly instructed and report understanding that locations/responses are irrelevant for determining payoffs. They fit a set of RL models to the data and conclude that participants are employing model-free learning that automatically assigns credit to irrelevant location/response features of the task. The model fits are done with state-of-the art methods and the authors thoroughly test for parameter and model recovery. Despite these commendable methodological features, I think the conclusions drawn from the results are fundamentally flawed. There is a bias against choosing a previously non-rewarded location/response, but the data cannot show that this bias is caused by model-free credit assignment and I am doubtful the data can convincingly show that the bias originates from any form of reward learning. My concerns and critiques are listed in detail below.

We deeply thank the reviewer for their dedicated reading and thoughtful comments. We provide below a point-by-point answer to all concerns.

1. Model-free and model-based behavior cannot be distinguished in the current bandit task. There may be automatic/mistaken credit assignment to locations or button presses, but there is no way to know which system or combination of systems is assigning that credit. Narrative instructions and correct answers to comprehension questions cannot fully guarantee correct model understanding or consistent correct model use throughout the task. For example, participants may start to use inaccurate simplifying heuristics or strategies during the task because of boredom, frustration, or a desire to save effort.

We acknowledge that outcome-irrelevant learning cannot be conclusively characterized as purely model-free, as at least some participants may still have acted according to a wrong model of the environment or used some type of heuristics regarding associations between reward and the outcome-irrelevant feature. We thank the reviewer for making this comment. We have now extensively revised the manuscript so that it does not refer to a model-free system, but instead to outcome-irrelevant learning. We now only refer to “model-free” in a dedicated discussion paragraph that addresses the nuances around model-free processing, as can be seen below. Critically, we assert that demonstrating automatic assignment of value to outcome-irrelevant action features is a meaningful contribution to the field of human learning and decision making, regardless of whether outcome-irrelevant learning is predominantly model-free or model-based.

<u>*The discussion now includes:*</u>

The reinforcement learning literature has argued for a dissociation between elaborative model-based processes, which rely on an internal representation of the environment, from model-free processes that learn based on action-outcome history alone (Balleine & Dickinson, 1998; Daw et al., 2011; Ding et al., 2025; Gläscher et al., 2010; Miranda et al., 2025). Given that participants were provided with elaborated instructions and quizzed to make sure they had understood these instructions, it seems unlikely that outcome-irrelevant learning is the result of a pervasive use of wrong cognitive models. However, we can not rule out that at least some participants might have formed wrong cognitive models of the task, making it challenging to confidently affirm that outcome-irrelevant learning is purely

model-free (Feher da Silva et al., 2023; Feher da Silva & Hare, 2020). Furthermore, recent work has questioned the existence of such perfect dissociation between model-free and model-based processes (Collins & Cockburn, 2020; Moran et al., 2019, 2021). Thus, future work should carefully address whether automatic outcome-irrelevant learning is model-free by definition or can also reflect residual model-based tendencies.

2. I don't follow the logic of the interpretations of the data from the extensive training experiment. The authors suggest that this is evidence of an automaticity that does not go away with experience. As the authors point out, this is opposite of what one would expect from a reward learning system, which should learn that locations/responses are unpredictive of reward. This raises a further question about why we should think that automatic/mistaken credit assignment to locations/responses is part of any reward learning system (model-based or model-free). Please provide some reasons as to why you claim that the extensive training results support the idea that reward learning systems automatically assign credit to locations/responses, rather than contradicting the idea that such responses are learned.

We thank the reviewer for this thoughtful comment and for the opportunity to clarify our theoretical position. Our argument involves two related but distinct claims, and we suspect that the distinction between them may not have been sufficiently clear in the original text.

(1) <u>Learning of outcome-irrelevant features (lower level)</u>: Our first claim is that people do learn the values of task-relevant yet outcome-irrelevant choice features (e.g., left/right in Experiments 1,2, and 4A or blue(cyan)/red(orange) colors in the now-added Experiments 3 and 4B). Across all experiments, we show robust reward-based credit assignment to these concrete features, even though they do not predict reward. Because this credit assignment is explicitly driven by reward history (i.e., value updating following reward or non-reward), we take this to be part of the "reward learning system" by definition. At this lower representational level, the system assigns value to action features irrespective of their true causal relevance. To further emphasize the fact that the phenomenon at hand is a value-based learning process, we have now added elaborated learning signatures and revised Figure 2 (see below). Specifically, these analyses extend beyond the single previous trial and show unequivocally that outcome-irrelevant learning is a gradual learning effect, rather than a mere bias from the previous trial alone. Specifically we now report that: (a) the influence of location-outcome history shows a gradient with the strongest influence from the very last trial, then decaying backward, in line with a learning rate effect (see report below and Figure 2E), (b) when counting the number of rewards that followed each location selection, the difference between these aggregated sums predict behavior (see report below, and Figure 2F).

(2) <u>Flexibility of strategy-level learning (higher level)</u>: Our second claim concerns a different level of abstraction: the level of choice policies or strategies. A fully flexible reward learning system should, in principle, be able to learn not only the value of specific features but also the value of using a particular strategy, e.g., whether relying on outcome-irrelevant features is beneficial or detrimental. Experiment 2 tests this higher-level flexibility. Participants were given extensive experience that should have enabled them to infer that a strategy sensitive to irrelevant features is counterproductive. If the reward learning system flexibly updated policies, we would expect a reduction in reliance on these irrelevant-feature values. However, we find that despite considerable opportunity, there is evidence that participants do not suppress or unlearn this strategy. Thus, this lower-level credit assignment is not easily overridden by

higher-level policy learning, leading to its characterization as automatic. Note that while we did observe some tendency for increased outcome-irrelevant learning across practice, this effect is uncertain statistically and should be interpreted with caution. Instead, our observations confidently show that the theoretical population almost certainly does not reduce outcome-irrelevant learning with practice (~100% probability of direction).

This pattern suggests a degree of automaticity or rigidity in how the reward learning system treats feature-level credit assignment. In other words, the system persistently assigns value to outcome-irrelevant features, and participants continue to rely on these values even when doing so is unhelpful. This is well in line with the event coding theory suggested by Hommel (2009), where some aspects of decisions are driven by automatic event coding processes. We have now revised the discussion to better clarify these points (within the possible word limit).

The results now include:

Model-agnostic regression signature for outcome-irrelevant learning

To illustrate the presence of outcome-irrelevant learning outside computational modeling, we focus on a subset of trials where the two outcome-relevant colors presented in a previous trial (trial n-1) are not reoffered in a subsequent trial (trial n; see Figure 2A). We aimed to demonstrate a signature of outcome-irrelevant learning, reflected in an increased tendency to repeat a previously chosen location as a function of the previous outcome (see Figure 2B; Ben-Artzi & Shahar, 2024). Note that examining the tendency to repeat/switch a location as a function of outcome reflects an outcome-dependent effect, which should not be confused with outcome-independent motor perseveration previously described extensively (i.e., tendency to repeat regardless of outcome; Miller et al., 2019).

Influence of outcome in one trial on the selection of outcome-irrelevant features in the next trial. To provide a main model-agnostic signature for outcome-irrelevant learning, we fitted a hierarchical Bayesian logistic regression estimating the effect of the *previous-outcome* (on trial *n-1*; Unrewarded/Rewarded) on the decision to repeat the same location choice on trial *n* (i.e., *stay_location*; Switch/Stay), when two different colors than before are offered (see Figure 2B). We found convincing results in both abstract and story conditions indicating that the probability of repeating the same location following a rewarded trial (47%/48% for story and abstract conditions, accordingly) was consistently higher than following an unrewarded trial (44%/43% for story and abstract conditions, accordingly; Story posterior median = .13, highest density interval ($HDI_{95\%}$) between .04 and .22, pd = ~100%; Abstract posterior median = .19, $HDI_{95\%}$ between .1 and .29, pd = ~100%; see Figure 2B). We found evidence in favor of some difference between conditions (interaction posterior median = -.06, $HDI_{95\%}$ [-.19, .07], pd = 83%). To illustrate the relationship between this model-agnostic signature and the λ parameter from out computational model we show a clear positive correlation in both simulated data (N=200, Posterior Pearson's r median = -.40, $HDI_{95\%}$ [-.53, -.27]; see Figure 2C) and in empirical data (N=504, pooled across the two conditions, Posterior Pearson's r median = -.43, $HDI_{95\%}$ [-.51, -.35]; see Figure 2D).

To further show that our model-agnostic signature reflects a reward-driven learning process, we conducted several additional analyses.

Influence of outcomes from the past three trials on selection of outcome-irrelevant features. In the analysis above (Figure 2B), we performed a regression in which reward on trial *n-1* predicted the probability of repeating the same location on the current *n* trial. We next repeated this same regression twice more: once using reward on trial *n-2* and once using reward on trial *n-3*. Thus, we estimated the reward effect separately in three regressions (for *n-1*, *n-2*, and *n-3*). In all three analyses, we only included trials in which the historical trial (whether *n-1*, *n-2*, or *n-3*) did not contain the same colors as those offered on the current trial. We find a gradual effect of past outcomes on choice. The outcome from the immediately preceding trial had the strongest influence (posterior median = .15, HDI95% [.09, .22], pd=~100%), a weaker but still reliable influence remains two trials back (posterior median = .08, HDI95% [.01, .14], pd=99%), and the effect is further reduced three trials back (posterior median = .03, HDI95% [-.03, .10], pd=85%). Thus, the regression coefficients systematically decrease with temporal distance, consistent with a learning-rate-like decay in the impact of rewards over time (see Figure 2E; see also Figure S2 for a confirmation that these effects do not appear among artificial agents who rely only on the very recent outcome).

Influence of cumulative reward on the selection of outcome-irrelevant features. We show that the model-agnostic cumulative reward difference between locations influences choice. Specifically, we calculated for each individual the cumulative reward difference at each point in time by adding 1 for each rewarded choice of a location and subtracting 1 for each unrewarded choice of a location. To show the effect extends beyond the very last trial, we accumulated only rewards obtained from the beginning of the block until trial n-2. We then use the difference in cumulative reward for each of the two locations to predict the next chosen location. As expected, given that locations are not predictive of outcome, on average, the cumulative reward difference between the locations was 0 (see Figure 2F). However, we show that when one location was arbitrarily more rewarded, participants were overall more likely to choose it (posterior median = .03, HDI95% [.02, .04], pd~=100%), and the lower one's estimated λ was the more they were affected by this noise in guiding their decisions (interaction posterior median = -.03, HDI95%[-.04, -.01], pd~=100%; see Figure 2F).

Perseveration estimates in the model-agnostic signature. Examination of Figure 2B shows an intercept below chance, which reflects a tendency to switch locations regardless of outcome. To illustrate this fact, Figure 2G provides a re-visualization of Figure 2B. Here, instead of coding the dependent variable as the probability of staying with the previous location selection (as in Figure 2B), we fit the same regression only predicting the chosen location (left/right coded 0 vs. 1, respectively) based on the previously chosen location (left/right) and its interaction with the previous outcome (unrewarded/rewarded). While this is a re-cap of Figure 2B in terms of added information, this allows us to more clearly demonstrate that participants tend to switch their chosen location regardless of reward (see grey dots in Figure 2G; posterior median for the effect of previously chosen location = -.17, HDI95% [-.20, -.13], pd~=100%). The figure then further demonstrates how outcome-irrelevant learning influences location selection. Specifically, after choosing the right-side location, participants were more likely to repeat that choice following a rewarded trial than an unrewarded one. In contrast, after previously choosing the left location, they were more likely to switch to the right location after an unrewarded trial than after a rewarded trial (interaction posterior median = .07, HDI95% [.04, .1],pd~=100%; see colored dots in Figure 2G). Importantly, the conclusion that the regression intercept reflects a location-switching

tendency is strongly supported by the negative $\rho_{spatial}$ parameter from our computational model (r=.58; see Figure 2H).

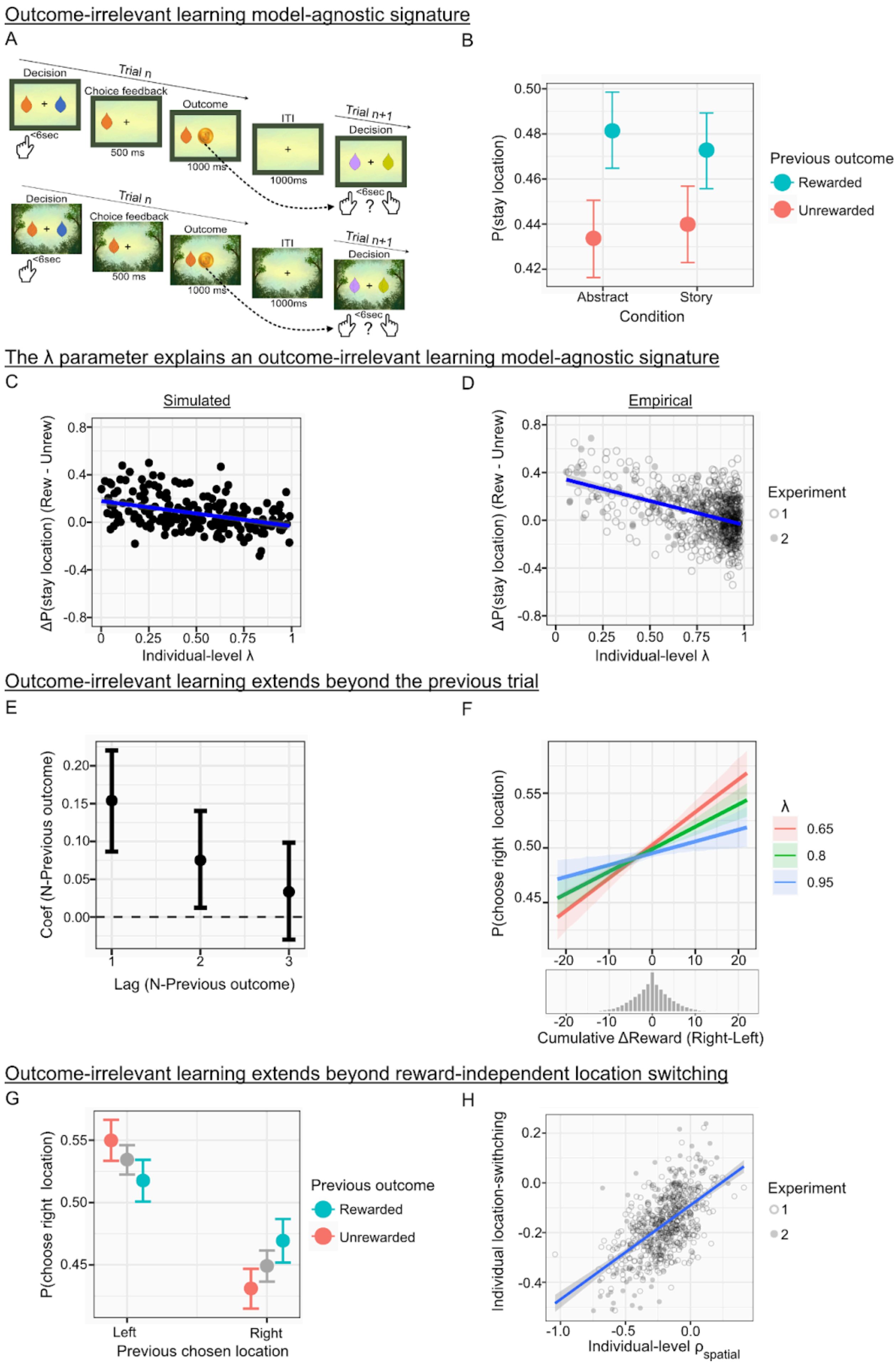

Outcome-irrelevant learning model-agnostic signature
A
Decision
Trial n
Choice feedback
Outcome
ITI
Trial n+1
Decision
<6sec
500 ms
1000 ms
1000ms
?
B
P(stay location)
0.50
0.48
0.46
0.44
0.42
Abstract
Story
Condition
Previous outcome
Rewarded
Unrewarded
The λ parameter explains an outcome-irrelevant learning model-agnostic signature
C
Simulated
ΔP(stay location) (Rew - Unrew)
Individual-level λ
D
Empirical
Experiment
1
2
Outcome-irrelevant learning extends beyond the previous trial
E
Coef (N-Previous outcome)
Lag (N-Previous outcome)
F
P(choose right location)
λ
0.65
0.8
0.95
Cumulative ΔReward (Right-Left)
Outcome-irrelevant learning extends beyond reward-independent location switching
G
P(choose right location)
Left
Right
Previous chosen location
Previous outcome
Rewarded
Unrewarded
H
Individual location-swithching
Individual-level ρ spatial
Experiment
1
2

Figure 2. Model-agnostic signature indicated outcome-irrelevant learning. To further confirm that participants indeed learned the value of outcome-irrelevant locations, we estimated a model-agnostic signature. **(A)** We focus on a trial sequence where colors offered on trial n-1 were not reoffered on trial n, allowing us to examine the model-agnostic effect of the outcome in trial n-1 on trial n location choice, without any interfering contribution from outcome-relevant learning. Note that according to current literature of human reinforcement learning, the outcome in trial n-1 should have no influence on the trial n choice, as two different colors are offered for selection. **(B)** We fitted a hierarchical Bayesian logistic regression model examining the influence of the previous outcome (from trial n-1) on the tendency to choose the same location (in trial n) when two different colors are offered (see panel A). We find that in both conditions, outcome-irrelevant learning tendencies are apparent, as indicated by a higher probability (~4%) of staying with the same location choice after a rewarded trial compared to an unrewarded one. The below chance intercept (middle point between unrewarded and rewarded trials) reflects a location-switching tendency as we illustrate further in panels G&H. **(C)** We simulated data using our model and task design to illustrate the relationship between the model-agnostic behavioral signature and the λ parameter. Y-axis values indicate for each simulated agent the difference in the probability of staying in the same location (when two different cards than before are offered, see A) following rewarded vs. unrewarded trials. As expected, agents generated using lower λ values show a bigger positive effect. **(D)** We repeat this process for empirical data from our two experiments, showing the negative relationship between λ and this model-agnostic behavioral signature. **(E)** To show that outcome-irrelevant learning signature extends beyond the previous trial, we extend the model-agnostic regression analysis shown in Figure 2B, from the previous trial to 2 and 3 trials back, showing a gradually decaying influence of a previously obtained reward on choice, always when different colors were now offered for selection. **(F)** We calculated an empirical cumulative reward difference between the two locations, while excluding the very last trial. We show that, on average, the difference is equal to 0, but when one location is by chance more rewarded than the other, choices of individuals with lower λ are strongly influenced by that arbitrary value difference. **(G)** Panel B shows an intercept below chance that reflects an outcome-independent location switching tendency. To illustrate this, we provide a re-visualization of our model-agnostic result from panel B, where the chosen location (left/right) is predicted by the previously chosen location and the previous outcome. As depicted by the grey points, we show that overall participants are more likely to choose 'left' after choosing 'right' and vice versa (i.e., location switching tendency regardless of reward). Importantly, we show that choice is further influenced by the previous outcome, making participants more likely to repeat a previously chosen location after a rewarded trial, and vice versa. **(H)** The explanation that the below chance intercept in Panel B is due to location switching tendency independent of the obtained outcome is supported by a strong correlation of the individual model-agnostic intercept in the regression model and the ρ spatial switching-perseveration parameter (r=.58).

The SI now includes:

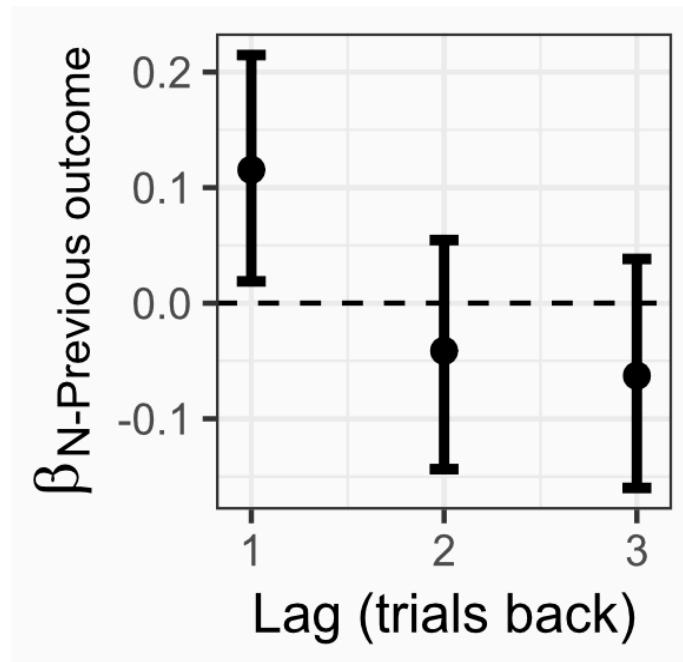

Figure S2. Validating the ability of our modal-agnostic signature regression analysis to estimate outcome-irrelevant learning from two and three trials back. The main text presents two regression analyses examining the influence of outcome on the selection of an outcome-irrelevant location, from two and three trials back (Figure 2E, main text). We wanted to ensure that if participants had been only influenced by the very last trial (*n-1*) and not the two or three trials back (*n-2/n-3*), then our regression coefficients would accurately reflect that. For this purpose, we simulated 200 agents with empirically derived parameters, but whose learning rate was artificially set to 1, reflecting learning that is constrained only to the very last trial (*n-1*). This means these agents should not show an effect on the n-2 or n-3 by definition, allowing us to test whether the regression analysis can, in fact, discover that. As expected, when fitting the logistic regression to data from the simulations, we find that only the outcome of the very last trial affects the probability of repeating the same location (in trial *n*), with no residual influence from earlier trials.

The discussion now includes:

A second key concern in arguing for automatic credit assignment is that, even after being clearly informed which action features were not predictive of reward, participants might still have uncertainty about the outcome-relevance of these features (for instance, because they mistrust the instructions). Such uncertainty could prompt them to deliberately assign value to outcome-irrelevant features and, through trial and error, test whether these features predict rewards. Previous studies have shown that, under conditions of state uncertainty, humans are adept at inferring through trial and error which action features are reward-predictive and which can be ignored (Niv, 2019; Niv et al., 2015; Song et al., 2022; Wilson & Niv, 2012). In Experiment 2, participants completed extended testing that gave them ample opportunity to learn through experience which action features were uninformative of reward. But even after three sessions comprising 600 trials in the same environment, and despite outcome-irrelevant learning leading to reduced monetary gains, participants continued to assign credit to outcome-irrelevant features. This aligns with a broader framework where, at a lower representational level, the reward learning system assigns value to task-relevant yet outcome-irrelevant features, as demonstrated across experiments. At a higher, strategy level, a fully flexible system should learn that relying on these features is counterproductive and reduce their influence with practice. Experiment 2 offered a substantial opportunity for this strategic adjustment, yet no reduction in outcome-irrelevant learning was observed. This indicates that higher-level policy learning does not override feature-level credit assignment, which continues to operate even when it is detrimental to performance.

3. The parameter fits from the full RL model indicate that decisions are based largely on color (lambda is close to 1 for most participants). Given the strong weighting in favor of color in the calculation of net Q-values, location will only be able to shift the choice from the option favored by color when the difference in color Q-values is very small. In other words, when it is unclear which color is better. It seems plausible that a deliberative decision-maker (i.e. model-based) might break approximate ties with some known irrelevant feature or heuristic rule simply to avoid spending time and effort to pick between two nearly equal options.This effort saving strategy may be more prominent in participants with low working memory ability who may find deciding between two similar options more effortful (or low working memory and low lambdas may both stem from low motivation to do either task well). Given the inability to distinguish model-based and model-free behavior in this bandit task, is there any way to rule out the possibility that participants use such a tie-breaking heuristic?

We thank the reviewer for this thoughtful comment. In principle, participants could break ties using a minimal-effort rule that does not require tracking task variables at all. For example, always choosing with the dominant hand whenever the bandits appear equally valuable. On this view, it is not obvious why participants would invest effort in tracking outcomes for the irrelevant feature across trials if their goal was simply low-effort tie-breaking.

That being said, we agree with the reviewer that a low-effort tie-breaking account is worth considering since participants might track a very simple outcome signal (e.g., whether the immediately preceding location choice was rewarded) and use it to bias subsequent selections of outcome-irrelevant features. If outcome-irrelevant learning served this function, we would expect a short-lived, local effect driven primarily by the last trial. Our updated analyses do not support that prediction. As shown in Figure 2, the influence of outcome extends beyond the immediately preceding trial. Specifically, Figure 2E shows positive regression coefficients for outcomes up to the last three trials, and Figure 2F shows an effect of rewards aggregated across the block. This broader temporal profile is difficult to reconcile with a simple, minimal-effort tie-breaking heuristic and instead suggests a more gradual value-learning process.

Importantly, we now provide an additional analysis showing that the influence of outcome-irrelevant values on behavior is by no means limited to cases where no other policy was available. We find that even in trials in which there was a substantial difference (>0.3) in the underlying expected values or in the latent model-derived empirical Q-values of the outcome-relevant colors, there was still a clear influence of previous outcome on location choice. While we acknowledge that estimated Q-values may not reflect the actual values held by the participant, this evidence still goes against the hypothesis that outcome-irrelevant learning is limited to a heuristic aimed at breaking ties.

We have now added a dedicated paragraph in the discussion to address this issue.

The discussion now includes:

Outcome-irrelevant learning may potentially serve as a “tie-breaking” mechanism in decisions made under complete uncertainty, where no reward-relevant information is available to guide choice (see SI for a relevant analysis). Such decisions have been described in the literature as "picking" rather than

"choosing," reflecting the absence of a rational basis for preference (Furstenberg et al., 2015, 2023; Ullmann-Margalit & Morgenbesser, 1977). It has been argued that true picking does not exist, since some internal or external influence always nudges the decision-maker toward one option over another (Leibniz, 1734). In this spirit, learning outcome-irrelevant noise, such as tracking which side of the screen temporarily tends to yield more rewards, could enable individuals to generate arbitrary yet structured decisions, as opposed to truly random ones (Sher et al., 2025). Under the assumption that truly random guessing is challenging to execute, individuals can rely on an internal coherent logic (Icard, 2021). Thus, such a mechanism of "arbitrary exploration" may be similar, yet potentially more realistic, than the "random exploration" that is often described in the literature (Wilson et al., 2021).

The SI now includes:

Outcome-irrelevant learning is not limited to decisions where no information is available in the outcome-relevant feature

To test whether outcome-irrelevant learning only serves as a tie-breaking mechanism, we explored whether the model-agnostic regression signature is also apparent when the outcome-relevant feature provides a strong signal for choice. Specifically, we repeated the regression analysis on a subset of trials where either the underlying expected values difference of the two colors was large (>0.3) and found that even in these trials participants are more likely to repeat a previously chosen location following a rewarded compared to an unrewarded trial (posterior median = .2, HDI95% [.1, .3], pd~=100%; see Figure S4A). As participants may not be aware of the underlying expected values, we show similar results on a subset of trials where the difference in model-derived Q-values for the offered colors is large (>0.3), and found the same effect (posterior median = .24, HDI95% [.13, .35], pd=~100%; see Figure S4B). While these analyses indicate that outcome-irrelevant learning also occurs when the outcome-relevant feature is far from chance-level, we acknowledge that the general conclusion that outcome-irrelevant learning is not merely a tie-breaking mechanism is constrained by the assumption that our estimates accurately reflect the values held by participants. Future experiments should therefore use designs that more directly and rigorously test this hypothesis.

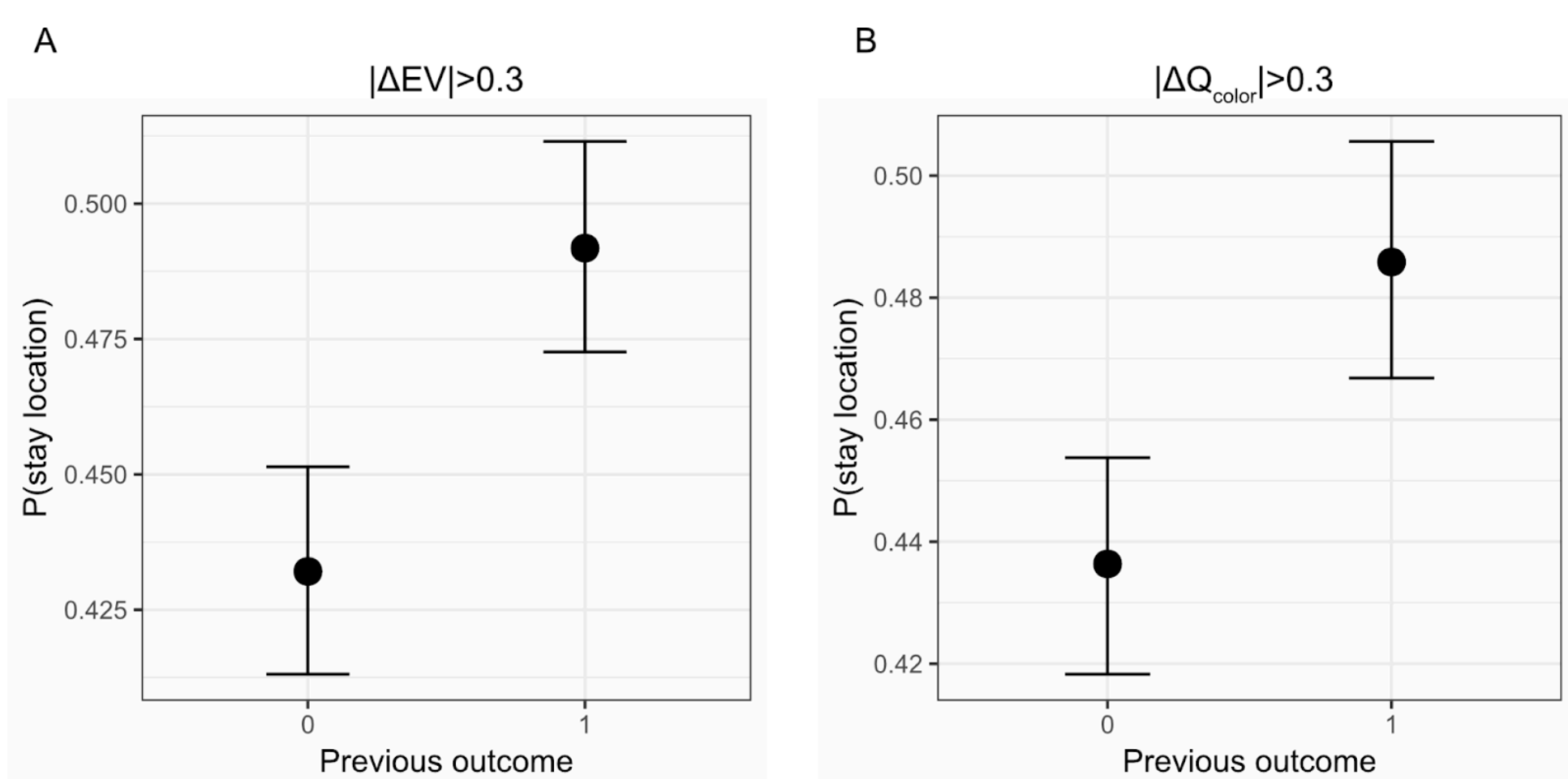

Figure S4. Outcome-irrelevant learning in trials with clear outcome-relevant information. (A) We also find that in a subset of "easy" trials where the current difference in expected values between the colors is large (i.e., >0.3), we find a clear indication of the outcome-irrelevant model-agnostic effect. **(B)**

As the underlying expected values of the random walk are not available for participants, we repeated the same analysis with model-derived Q-values for the outcome-relevant feature and found the same effect.

4. Do lower values of lambda lead to lower payoffs in addition to lower accuracy? The bandit payoffs are probabilistic so making the technically correct response will not always yield reward, and conversely, the incorrect response is sometimes rewarded. Participants don't get direct feedback on being correct or incorrect, they only observe a reward or not. Payoffs on tasks with drifting probabilistic rewards are famously insensitive to the use of optimal versus (somewhat) suboptimal strategies (e.g., Kool et al 2016 https://doi.org/10.1371/journal.pcbi.1005090).

Thank you for this comment. We have carefully examined and found outcome-irrelevant learning to be significantly associated not only with accuracy, but also with mean payoffs, in both Experiments 1&2 (see Figure S1; Note that the difference in the intercepts in Figure S1 (below) is due to different random walk sets used in the two experiments). We agree that payoffs are not always sensitive to optimal policies, such as in the case of the two-step task examined by Kool et al. (2016). Yet, in our case, where a single-stage multi-armed bandit is used, we are confident that λ has an effect on observed rewards - as can be seen below.

The SI now includes:

Previous studies have claimed that drifting probabilistic rewards might not be sensitive to the use of optimal strategies (Kool et al., 2016). We therefore further examined whether outcome-irrelevant learning was associated not only with reduced accuracy but also with lower payoffs in the task, which are what participants observed in the task. We found a substantial association with lower λ resulting in lower payoffs (posterior median = .06, HDI95% [.03, .09], pd =~100%) and Experiment 2 (posterior median = .08, HDI95% [.03, .12], pd =~100%; see Figure S1). Note that in Figure S1, the different intercepts between Experiment 1 & 2 pertain to the changes in the random walk, while the association between lambda and mean payoffs (i.e., slope) remains the same.

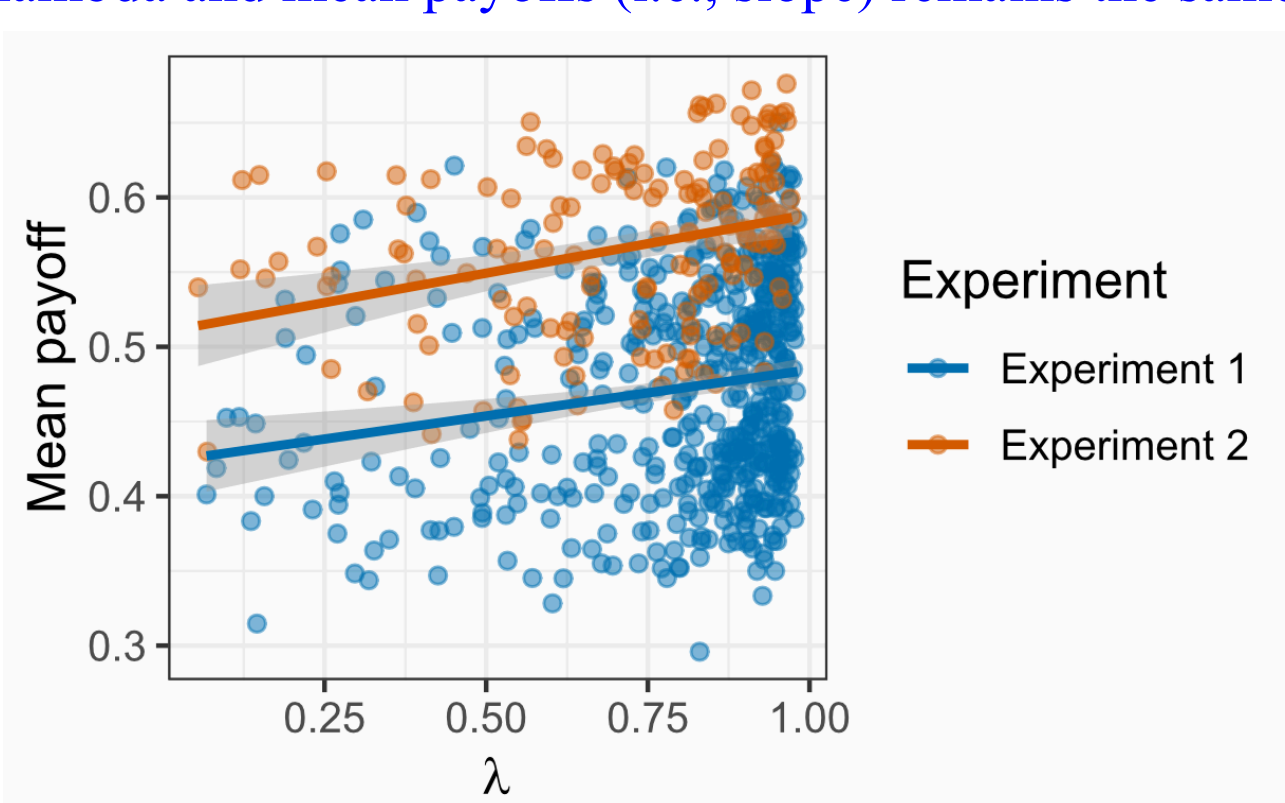

Figure S1. The association of λ with mean payoffs in the task, across both experiments. We find a positive association between λ and the mean payoff in the task. Note that the different intercepts are due to the different random walk sets used in the two experiments, and do not reflect a difference in the association between λ and mean payoffs, which was similar in both experiments.

5. Can we distinguish mistaken credit assignment to location/response from responding randomly on a subset of trials? The locations are not predictive of reward and the learning rates listed in Table 1 are

modest (~ 0.3). Therefore, if the locations are assigned credit, the Q values learned for each location should be very similar on most trials. The contribution of the location Q values to making a left choice is Q_L/(Q_L + Q_R) or ~ ½, if Q_L and Q_R are similar. So, another plausible alternative model is one in which lambda is a measure of a tendency for random responding instead of mistaken credit assignment.

We would first like to point out that α is not equal to 0.28. This impression arose due to confusion about Table S1, for which we apologize. All parameters in this table are in the unconstrained scale. That is, α, λ, and η parameters are in their natural values rather than the transformed values (i.e., constrained to be between 0 and 1). The α value in Table S1 corresponds to 0.57 on the transformed scale (i.e., constrained to be between 0 and 1). This is now emphasized in the table note.

Based on the Reviewer's comment, we provide in the main text three additional analyses that demonstrate that we can, in fact, distinguish mistaken credit assignment to location/response from responding randomly on a subset of trials.

First, we simulated data of 50 agents with empirical parameters (including color-perseveration and location-switching tendencies). However, we eliminated outcome-irrelevant learning in these agents by rigging λ=1. We further rigged β = 0 in one third of the trials, representing a potential lapse of attention and a value-independent random choice. This mimics the scenario described by the reviewer, in which there is no outcome-irrelevant learning, yet participants tend to respond at random due to momentary task disengagement. We find that this group of agents does not show the model-agnostic reward-driven signature we associate with outcome-irrelevant learning (see Figure 7C, below). Moreover, when fitting a model to the behavior of these agents, we show that we can recover the high λ (recovered population-level posterior median = .99), and are not mistaking the lapses of attention as outcome-irrelevant learning. Interestingly, the estimated β is 1.2, which is two-thirds of the true simulated β of 1.8, indicating that the model was able to capture the weighted average, where on two-thirds of the trials, β is 1.8, and on one-third it is 0. Therefore, we are confident that random responding does not appear by mistake in our analysis as outcome-irrelevant learning.

Second, the differences between Q-values for the outcome-irrelevant dimension are substantial at times. Specifically, we examined and found that the Q-value difference between right and left (when spatial-motor is outcome-irrelevant) to be 0.27, which is comparable to the value difference on the outcome-relevant dimension (see Figure S5). Therefore, we do get substantial variance in the differences of Q-values for the outcome-irrelevant dimension.

Finally, we note that the β parameter in the model (capturing random choice behavior) and the λ parameter (capturing choice based on location values) are distinct, as demonstrated by a successful parameter recovery. To further demonstrate this using empirical data, we fitted a multiple regression analysis showing that the magnitude of the model-agnostic signature is negatively associated with the λ parameter (see Figure 7A) but positively associated with the β parameter (see Figure 7B). This is to be expected, as the model-agnostic signature reflects a reward-driven behavior that can not arise from the behavior of a random responder.

The results now include:

Inattentive responding does not lead to outcome-irrelevant learning.

Across our work, we reported a reward-driven stay/switch behavioral effect in the outcome-irrelevant dimension (see Figures 2, 4, and 5), which is tightly associated with the λ parameter (Figure 2). We thus interpreted this behavioral effect as a direct, model-agnostic signature of outcome-irrelevant learning. Here, we further aimed to show that this signature effect cannot arise from random responding that can arise due to task inattention.

We addressed this question using three independent analyses. First, we simulated agents using empirically derived parameter values (i.e., learning rate, color-perseveration, and location-switching tendencies). We, however, eliminated outcome-irrelevant learning in those agents by fixing λ to 1. We further introduced attentional lapses in one third of the trials for all agents by temporarily setting $\beta = 0$, resulting in value-independent choices on those trials. Nevertheless, we were able to recover λ as equal to 1 (see SI). Importantly, an in-silico analysis revealed no evidence for a reward-driven stay/switch behavioral effect in the outcome-irrelevant dimension (see Figure 7C). This demonstrates that arbitrary random responding alone cannot produce the empirical behavioral signature we associate with outcome-irrelevant learning.

Second, across Experiments 1&2, we fitted a multiple linear regression predicting each individual's reward-driven stay/switch behavioral effect in the outcome-irrelevant dimension (see Figures 2, 4, and 5) using both their λ and β estimates. Convincingly, we found that a one-unit reduction in β is associated with a 3% decrease in the aforementioned effect (posterior median = 3%, HDI95% [1%, 5%], pd=~100%; see Figure 7B). Therefore, if anything, random responding is associated with a lower signature effect in the outcome-irrelevant dimension. In contrast, λ values were again found to be strongly associated with the model-agnostic signature (posterior median = -.53, HDI95% [-.6, -.45], pd=~100%; see Figure 7A). This asserts that the reward-driven stay/switch behavioral effect in the outcome-irrelevant dimension reflects a behavior that can not arise from a random responder.

Finally, to further illustrate our point that random responding can not give rise to the aforementioned, we fitted a linear multiple regression analysis predicting working memory capacity by λ while controlling for inattentive/random responding. We find that even when controlling for β and the general choice accuracy in the bandit task, there is a clear association between working memory capacity and the λ parameter (posterior median = 1.32, HDI95% [.92, 1.73], pd=~100%; see Figure 7D).

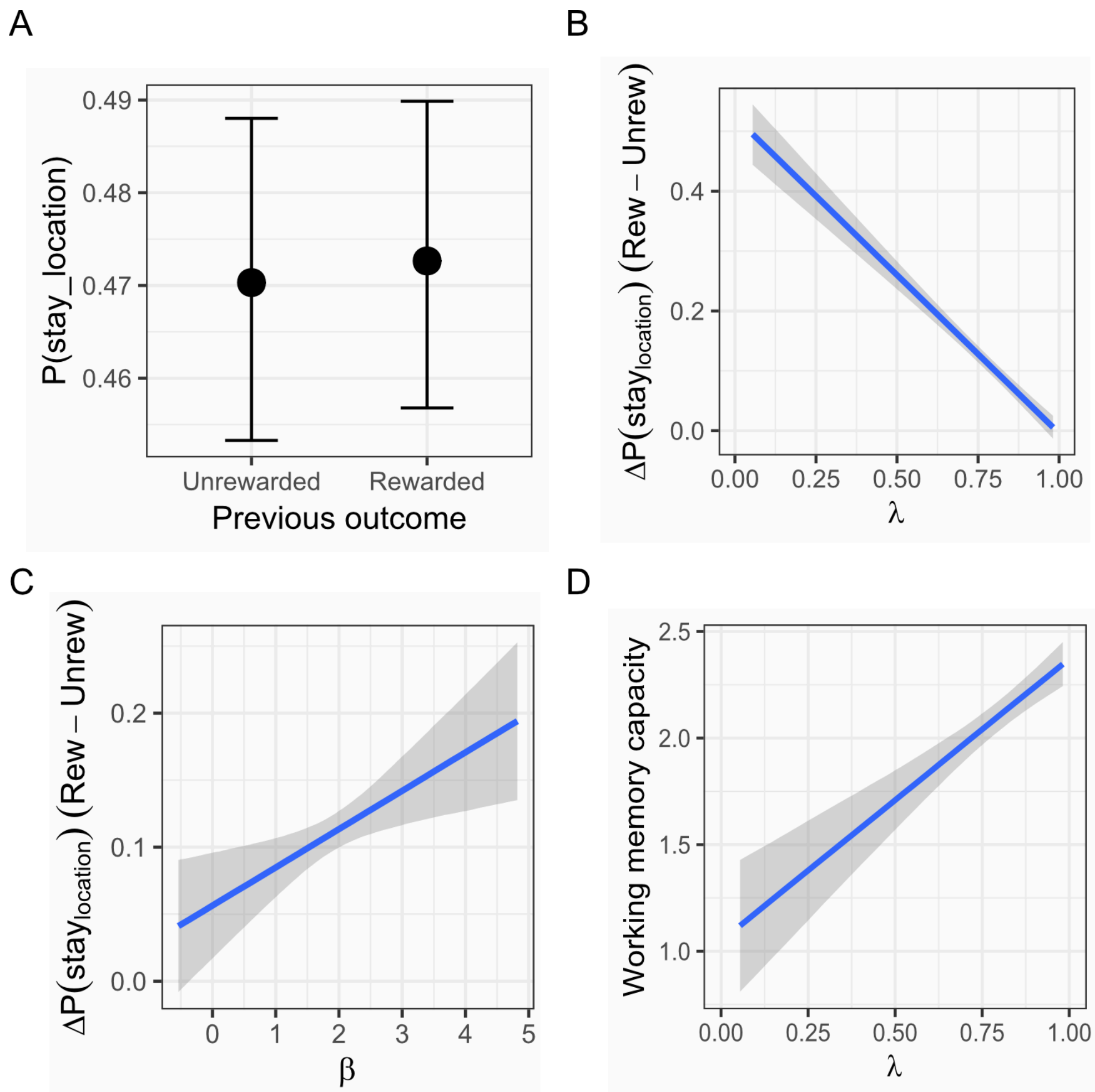

Figure 7. Inattentive responding does not lead to outcome-irrelevant learning. (A) To ensure that the reward-driven stay/switch behavioral effect in the outcome-irrelevant dimension is not driven by random responding, we simulated agents with empirical parameters but with no influence of outcome-irrelevant learning on decision (i.e., λ=1). To explore the possibility that outcome-relevant learning combined with inattentiveness gives rise to outcome-irrelevant learning, on one third of the trials, we included a "lapse of attention", where β is temporarily set to 0. We find that these agents clearly do not show the model-agnostic effect. Next, we predicted for each individual the model-agnostic signature based on both λ and β parameters. Despite λ and β being positively correlated among individuals in our sample, the model-agnostic signature is differentially associated with these parameters. **(B)** While higher degrees of λ are associated with a smaller outcome-irrelevant learning signature, **(C)** higher degrees of β are associated with a greater outcome-irrelevant learning signature. This is to be expected, given that random responding can not generate the effect. **(D)** Finally, we show that the association between λ and working memory is robust even when controlling for both β and general choice accuracy in the bandit task. That is, the association is not due to inattentive responding in both tasks.

The SI now includes:

Recovery of λ in agents with λ=1 and attentional lapses

To show that inattentive responding can not account for the outcome-irrelevant learning effect, we simulated data of 50 agents using empirically derived parameters, but eliminated outcome-irrelevant learning in those agents by fixing λ to 1. We further included attentional lapses in one third of the trials for all agents by temporarily setting $\beta = 0$. Yet, we were able to recover λ as equal to 1 (posterior median = .99, HDI95% [.93, 1]; see Figure S7).

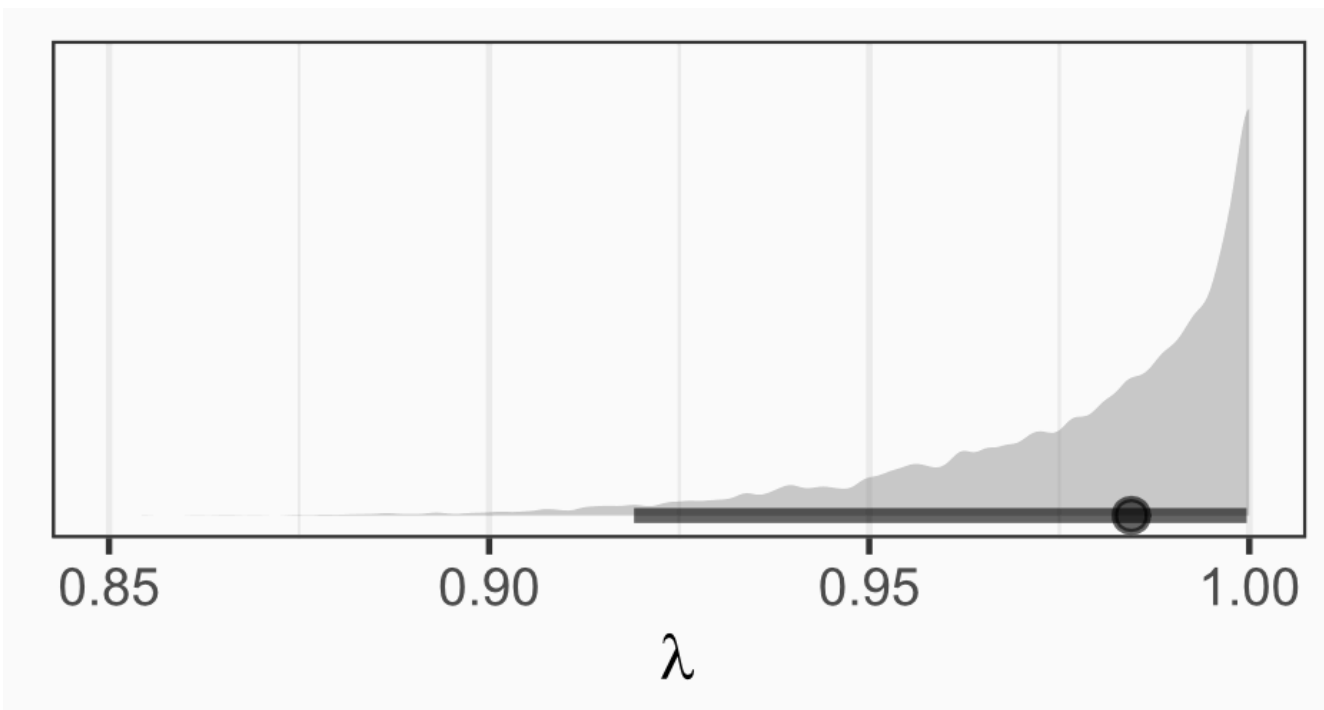

Figure S7. Recovery of λ for "inattentive" agents. We simulated data of 50 agents with empirically derived parameters, but whose λ is fixed to 1. We further introduced "attentional lapses" by temporarily setting $\beta = 0$ on one third of the trials. We find that even under those conditions, of occasional value-independent choice, λ is still recoverable.

The SI now includes:

Model-estimated Q-value difference in Experiments 1&2

We were concerned that the Q-values differences for outcome-irrelevant dimensions might show restricted variance since they were driven by a random association with the outcome (due to their irrelevant nature by design). We therefore examined and found that the differences in Q-values of the two outcome-irrelevant locations were substantial when estimating them from the model (see Figure S5). Thus, while arbitrary in nature, outcome-irrelevant values have a predictable influence on behavior.

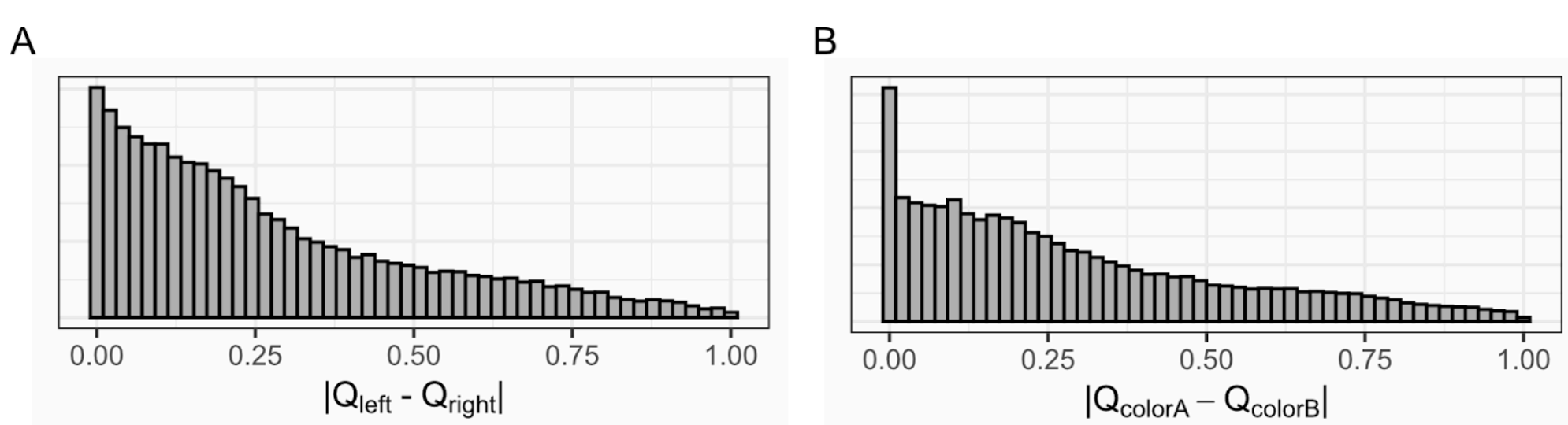

Figure S5. A histogram for the absolute model-derived Q-value difference between the (A) Left and Right outcome-irrelevant locations, and (B) between the two outcome-relevant currently offered colors. This shows that the variance of outcome-irrelevant Q-value differences was not minimal, and could show substantial value differences, despite being driven by random association with reward.

Does the full model(with lambda scaling color vs location influence) fit better than this alternative with lambda scaling color's influence relative to a random choice)?

Thank you for pointing this out. We agree that it is important to distinguish outcome-irrelevant learning from random responding within the modeling framework. As shown in our previous response, random responding can not account for reward-driven effects in the outcome-irrelevant dimension. Accordingly, based on our simulations, parameters that capture random responding (such as β or related noise parameters) cannot produce the behavioral signature in question, which is tightly linked to the λ parameter.

Regarding the reviewer's question about model comparison between our model and an alternative model in which a parameter scales outcome-relevant learning versus random responding, we note that such a model is equivalent to our baseline model in which λ is fixed to 1, and β captures this trade-off. This justified concern was therefore addressed in our model comparison. Specifically, we designed a baseline model in which λ was fixed to 1, such that choices reflected a mixture of value-based learning for the outcome-relevant feature and random responding. We compared this model with one in which λ was free to vary. Using simulated data, we were able to reliably distinguish between these models in model recovery. Empirically, the winning model was the full model including a λ free parameter, indicating that allowing choices to follow location values provides a better account of behavior. We therefore conclude with confidence that what we characterize as outcome-irrelevant learning cannot be a misinterpretation of underlying random behavior.

The fact that a substantial minority of participants have lambdas below 0.5 in Fig. 1D makes me think that this parameter is not only/primarily capturing the influence of a mistaken/automatic tendency to use locations in forming net Q-values. Relying on unpredictive location/responses *more* than the predictive colors seems unlikely for any RL system in healthy adults.

We agree with the reviewer that participants are generally not expected to have λ values below 0.5. However, it is important to note that the behavior of participants who show λ values below 0.5 is predictable, rather than random. Notably, their model-agnostic effect is strongest among all participants (see Figure 2D), indicating their parameter estimates are mostly not a statistical artifact but rather a true characterization of behavior. As can be seen in the simulation shown in Figure 2C, individuals simulated with λ values below 0.5 show a similar model-agnostic signature to what is seen in empirical data. As the reviewer suggested, these "extreme" participants may have formed a wrong cognitive model of the task, despite clear instructions and extensive practice. Nonetheless, their behavior remains reward-driven and systematically predictable. Furthermore, across experiments, the outcome-irrelevant feature consisted of only two options (e.g., left and right locations or blue and orange colors) that were constantly available, whereas the outcome-relevant feature comprised four options, only two of which were presented on each trial. This asymmetry may have made the outcome-irrelevant feature comparatively easier to track and associate with reward outcomes. Future experiments could test whether equating the two features in terms of the number of options and their availability alters λ estimates.

We further wanted to validate that λ estimates in our model do not reflect a statistical artifact. When behavior approaches randomness (β=~0), λ loses identifiability. Thus, to rule out the possibility that $\lambda < 1$ simply reflects low β estimates, we reevaluated the association between λ and the model-agnostic signature using only participants whose β values were above the median ($\beta > 1.8$), ensuring that estimates

reflected genuinely value-guided behavior. We show that even for this group of participants, whose behavior was clearly identifiable (mean group β = 2.5), λ clearly captures outcome-irrelevant learning tendencies, as indicated by a strong negative association between λ and the model-agnostic signature (r=-.55).

<u>*The SI now includes:*</u>

The association between λ and the model-agnostic outcome-irrelevant learning signature is not due to misestimation in random responders

To ensure that λ genuinely reflects outcome-irrelevant learning even among individuals who are not simply behaving noisily, we performed an additional analysis restricted to participants with β values above the median (β > 1.8). This subset represents individuals who show relatively strong value-based choice consistency, reducing the possibility that λ merely compensates for randomness in behavior. Even within this higher-β group, λ was strongly associated with the model-agnostic signature of outcome-irrelevant learning (r=-.55; see Figure S7), reinforcing that λ captures a meaningful computational tendency rather than an artifact of low β.

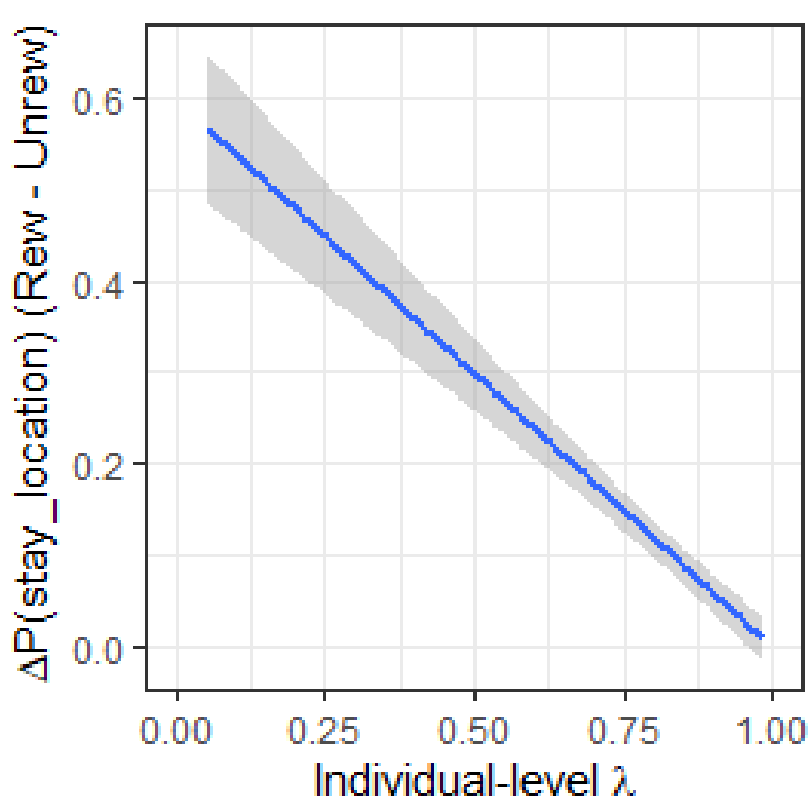

Figure S8. The association between λ and the model-agnostic outcome-irrelevant learning effect persists even when restricting the analysis to participants with high β values (β above the median).

6. Related to the idea that lambda may be an additional measure of randomness beyond the inverse temperature parameter, what is the correlation between the two parameters? Do they trade off against each other? The authors state that they inspected pairs plots for the fitted parameters. Could you please add those to the supplementary material so that readers can see the correlations between lambda and all other estimated model parameters?

The correlation of individuals' β and λ parameters across the two experiments is 0.27, and is now reported in the manuscript together with MCMC posterior pairs plots that we added to the SI, alongside a correlation matrix for all individual-level model parameters.

We fully understand and accept the reviewer's concern that λ might absorb variance in behavior that is actually due to random responding, rather than credit assignment to the outcome-irrelevant dimension. However, we argue that the empirical positive correlation between β and λ cannot be explained by such model misspecification and instead reflects a genuine empirical finding. Specifically, β governs stochasticity and random responding, whereas λ captures an arbitrary but *predictable* pattern of credit assignment. This distinction is supported by several converging results, reported in detail in our

response above, which we recap here:

(1) When we simulated a model where λ is fixed to 1, but β varies, we recovered λ ≈ 1. This was true even when we further introduced bursts of random response in one third of the trials by temporarily setting β = 0 (mimicking momentary attentional lapses). Thus, variance due to random responding was by no means absorbed by λ estimates. We believe this is to be expected given that low λ values reflect a *predictable* rather than *random* behavior.
(2) We observed a tight and selective association between predictable, reward-driven stay/switch behavior and λ - but not β - further indicating that λ does not reflect random responding.
(3) Outcome-irrelevant behavioral effects remained pronounced even among participants with high β.
(4) Model comparison showed that a random-responding component alone is insufficient to predict out-of-sample behavior.

We therefore postulate that λ is mechanistically incapable of conveying random responses. The positive correlation between β and λ should be taken as reflecting a true empirical relationship, whereby participants who are more engaged in the task show reduced outcome-irrelevant learning. This interpretation aligns well with our theoretical framework, which proposes that automatic credit-assignment processes are regulated by cognitive control mechanisms that can attenuate their behavioral impact. Consistent with this view, the literature on automatic cognitive processes strongly supports the idea that such processes can be cognitively regulated, placing our claims well within the existing theoretical framework.

The SI now includes:

Empirical parameter identifiability

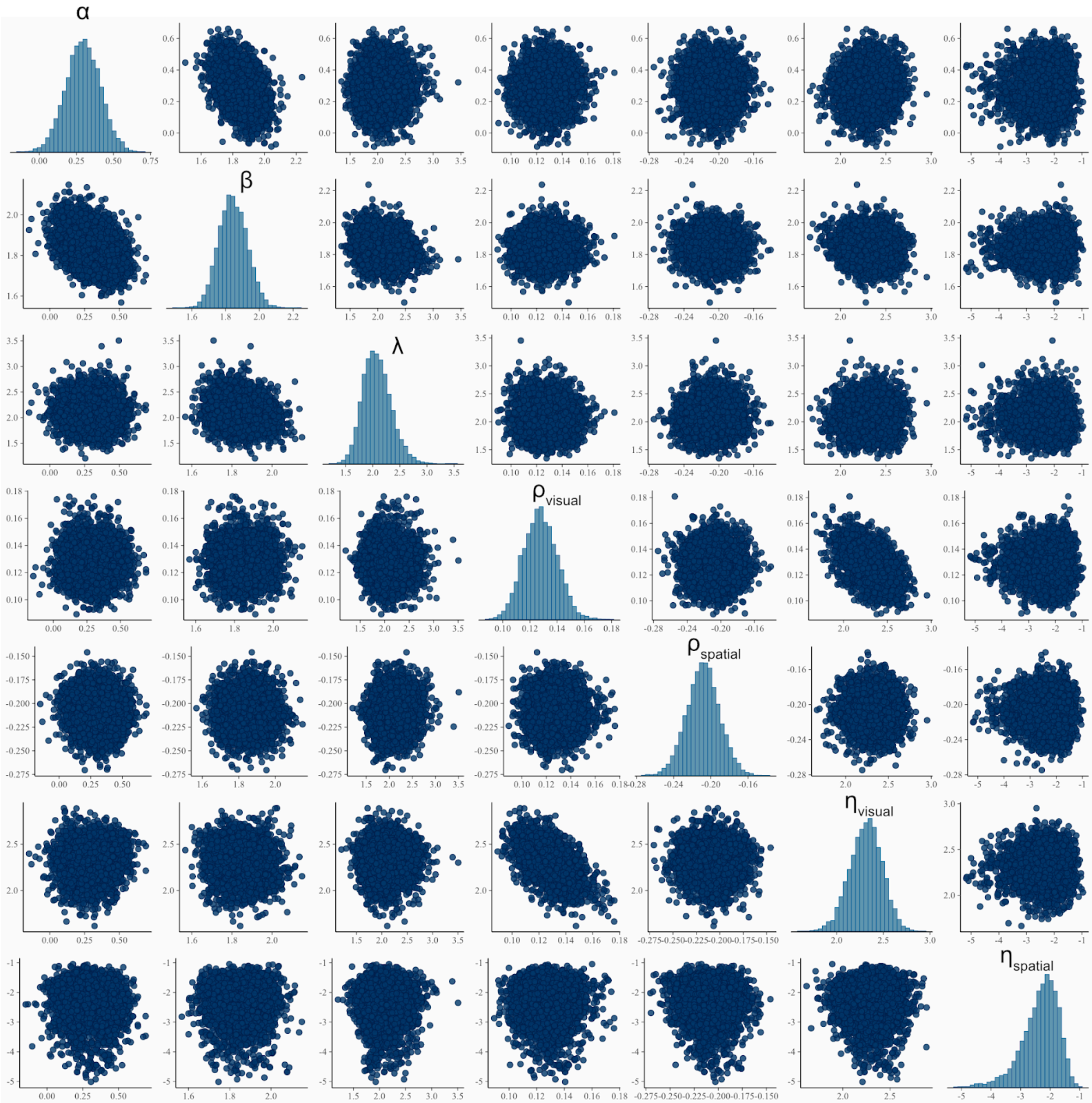

Figure S9. MCMC posterior sampling empirical pairs plot. To assess potential trade-offs between the outcome-irrelevant learning parameter λ and other estimated model parameters, we examined pairs plots of MCMC posterior samples for population-level parameters in the abstract condition of Experiment 1. The plots show a unimodal posterior distribution for all model parameters, and only weak correlations between λ and inverse temperature β as well as between λ and other parameters, indicating limited parameter trade-offs and good identifiability.

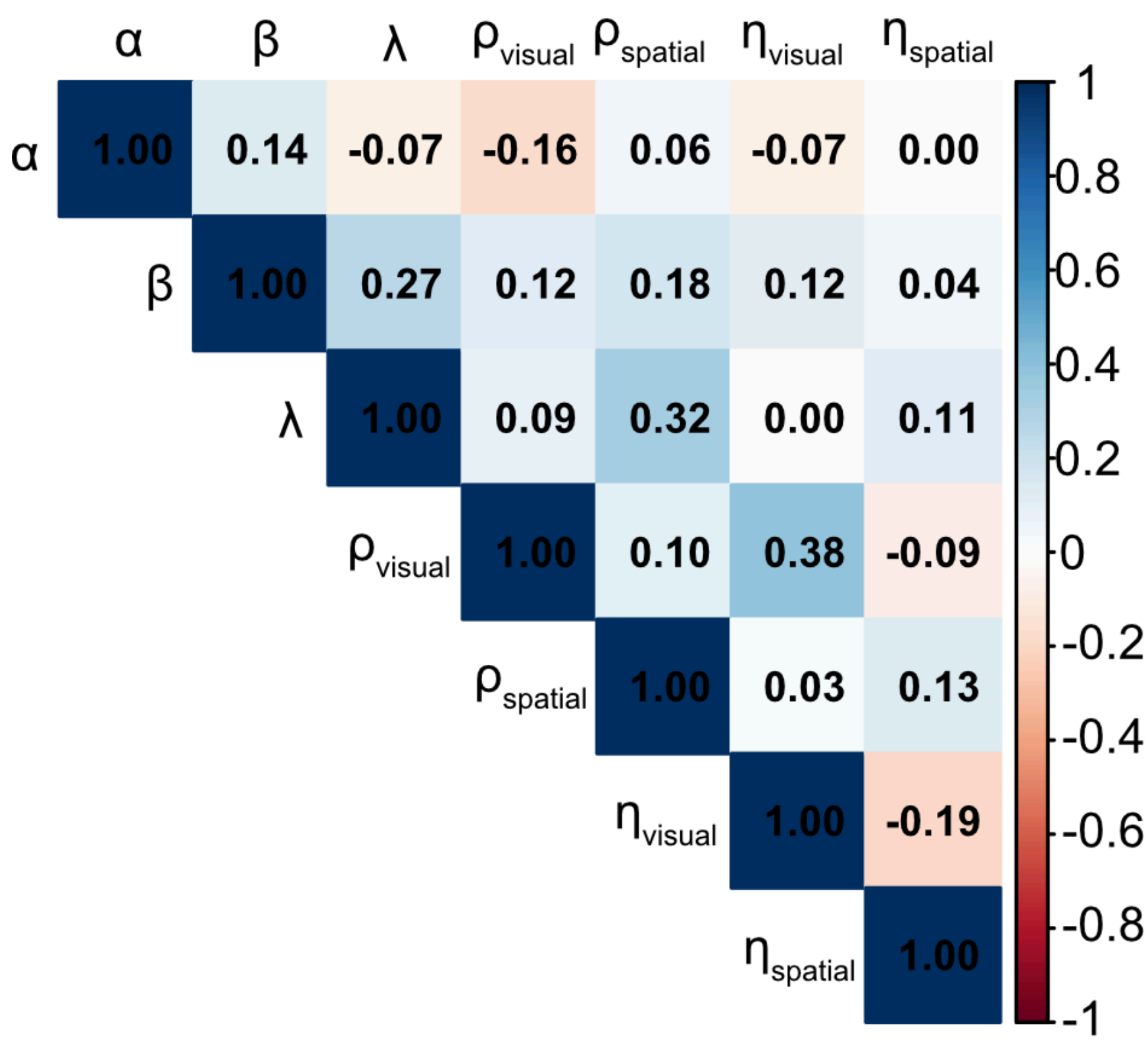

Figure S10. Correlations between individual-level parameter estimates. To examine empirical associations between individual-level parameter estimates, we computed Pearson correlations between individuals' posterior mean parameters across Experiments 1 and 2. Correlations were generally weak, indicating that individual differences in the outcome-irrelevant learning parameter λ did not strongly depend on individual differences in other model parameters.

7. Does the full RL model fit the data better than a simple categorical regression model? The current paper reuses data from Feher and colleagues 2023, which reported that a simple categorical regression model fit their data better than RL models did. Those authors claim that this result indicates that participants' true strategies deviate substantially from the learning approach(es) specified in the RL model. A similar sanity check on the appropriateness of the RL model specification for the bandit task data would be helpful to include.

We compared out-of-sample predictive performance between the reinforcement-learning model and a categorical regression model using leave-one-out cross-validation (loo; Vehtari et al., 2017). In the categorical regression model, choices were predicted based on the reward received on the preceding trial, while in the reinforcement learning model, behavior was predicted using Q-learning. The reinforcement-learning model showed substantially superior predictive performance, indicating that incremental value updating captured behavior much better than a simple win-stay/lose-shift rule.

The SI now includes:

Comparing the computational model to a simpler categorical regression model

Previous studies indicated that a categorical regression model can fit the data better than RL models, indicating that participants' true strategies might deviate substantially from the learning

approaches specified in an RL model. We therefore compared whether our winning computational model leads to a better fit than a simpler logistic regression, where choice (i.e., which of the two colors is chosen) is predicted only by the previous outcome (unrewarded/rewarded). Given that only two out of four colors are offered on each trial, we constrained our comparison to predicting choices on trials in which the previously chosen color was reoffered. We found that the computational model predictions were markedly better than the ones made by a categorical regression capturing only win-stay lose-shift behavior for the chosen color (Experiment 1 Story Δelpd = 966, SE = 66).

Minor points

8. Why constrain the learning rate to be equal for relevant and (automatic) irrelevant features?

We simulated data from a model that included both λ and two learning rates, but found that this model did not recover sufficiently well. Specifically, just as learning rates are by definition not identifiable when β is close to 0, the learning rate for the irrelevant feature is not identifiable when λ is close to 1. However, whereas β is seldom close to 0 in empirical data, this is not the case for λ in the current fit, where individuals can show λ values near 1. Importantly, this does not imply that these two learning rates are psychologically identical. Rather, it suggests that our current design cannot separate them, and future experiments should aim to create a design that better differentiates between these two parameters. We have now mentioned this clearly in the methods and limitations sections.

The methods now include:

We constrained the learning rate to be identical for the relevant and irrelevant features because allowing a separate α for the irrelevant dimension would introduce a non-identifiable $\alpha_{irrelevant}$ parameter when λ is estimated to be high. This issue can emerge in a substantial portion of participants whose λ values are close to 1.

The discussion limitation section now includes:

Finally, the current design does not allow us to reliably estimate a separate learning rate for each action feature. Thus, future experiments should aim to establish whether the learning rate for outcome-irrelevant dimensions is substantially different from that of outcome-relevant dimensions.

9. The authors write on line 753 that, “Positive values of rho reflected a bias toward repeating previously chosen features, whereas negative values captured a tendency to explore alternatives.” This is not strictly true when combining equations 5 and 6. Instead, the value of both rho and eta determine the bias toward repeating previously chosen features. If both have the same sign, then there is a bias to repeat, but if they have opposite signs, then there is a bias against repeating. This is important because Table 1 shows negative values for rho_spatial and eta_spatial, and if looking at rho alone readers might mistake it for a bias against repeating spatial locations.

We sincerely apologize for failing to mention in Table S1 that the parameters are reported in the unconstrained scale. We have now made an effort to make it clear that the two η parameters were constrained to be within 0 and 1 (i.e., logit transformation). Therefore, the negative values of $\rho_{spatial}$ indeed reflect a reward-independent side-switching bias, while the positive values of ρ_{visual} reflect a reward-independent color-repetition bias across the reported experiments.

The description of the computational model now includes:

The model included a mechanism to account for reward-independent tendencies to repeat or switch previously selected features. This was implemented by updating E-values for each chosen feature according to a feature-specific parameter (ρ_f; Eq. 5). Positive values of ρ reflected a bias toward repeating previously chosen features, whereas negative values captured a tendency to explore alternatives. Before each choice, the E-values decayed at a rate determined by a feature-specific parameter η_f (constrained between 0 and 1 using a logistic transformation), giving greater weight to more recent choices (Eq. 6).

$$[5] \quad E_f = E_f + \rho_f$$

$$[6] \quad E_f = E_f \cdot \eta_f$$

Table S1 note was corrected to:

"Values represent unconstrained (i.e., before logistic transformation) posterior medians with 95% HDIs in brackets."

References

Afshar, N. M., Cinotti, F., Martin, D., Khamassi, M., Calu, D. J., Taylor, J. R., & Groman, S. M. (2023). Reward-Mediated, Model-Free Reinforcement-Learning Mechanisms in Pavlovian and Instrumental Tasks Are Related. *Journal of Neuroscience*, *43*(3), 458–471. https://doi.org/10.1523/JNEUROSCI.1113-22.2022

Balleine, B. W., & Dickinson, A. (1998). Goal-directed instrumental action: Contingency and incentive learning and their cortical substrates. *Neuropharmacology*, *37*(4–5), 407–419. https://doi.org/10.1016/s0028-3908(98)00033-1

Ben-Artzi, I., & Shahar, N. (2024, October 10). *Outcome-Irrelevant and State-Independent Learning Mechanisms in Human Reinforcement Learning*. NeurIPS 2024 Workshop on Behavioral Machine Learning. https://openreview.net/forum?id=JADzOpkqiU

Collins, A. G. E., & Cockburn, J. (2020). Beyond dichotomies in reinforcement learning. *Nature Reviews Neuroscience*, *21*(10), 576–586. https://doi.org/10.1038/s41583-020-0355-6

Collins, A. G. E., & Frank, M. J. (2013). Cognitive control over learning: Creating, clustering, and generalizing task-set structure. *Psychological Review*, *120*(1), 190–229. https://doi.org/10.1037/a0030852

Daw, N. D., Gershman, S. J., Seymour, B., Dayan, P., & Dolan, R. J. (2011). Model-Based Influences on Humans' Choices and Striatal Prediction Errors. *Neuron*, *69*(6), 1204–1215. https://doi.org/10.1016/j.neuron.2011.02.027

Dill, M., & Fahle, M. (1998). Limited translation invariance of human visual pattern recognition. *Perception & Psychophysics*, *60*(1), 65–81. https://doi.org/10.3758/BF03211918

Ding, W., Cockburn, J., Simon, J. P., Johri, A., Cho, S. J., Oh, S., Feusner, J. D., Tadayonnejad, R., & O'Doherty, J. P. (2025). *Model-based and model-free valuation signals in the human brain vary markedly in their relationship to individual differences in behavioral control* (p. 2025.09.17.676398). bioRxiv. https://doi.org/10.1101/2025.09.17.676398

Dreisbach, G., & Haider, H. (2008). That's what task sets are for: Shielding against irrelevant information. *Psychological Research*, *72*(4), 355–361. https://doi.org/10.1007/s00426-007-0131-5

Eckstein, M. K., & Collins, A. G. E. (2020). Computational evidence for hierarchically structured reinforcement learning in humans. *Proceedings of the National Academy of Sciences of the United States of America*, *117*(47), 29381–29389. https://doi.org/10.1073/pnas.1912330117

Estes, W. K. (1948). Discriminative conditioning; effects of a Pavlovian conditioned stimulus upon a subsequently established operant response. *Journal of Experimental Psychology*, *38*(2), 173–177. https://doi.org/10.1037/h0057525

Foster, J. J., Bsales, E. M., Jaffe, R. J., & Awh, E. (2017). Alpha-Band Activity Reveals Spontaneous Representations of Spatial Position in Visual Working Memory. *Current Biology*, *27*(20), 3216-3223.e6. https://doi.org/10.1016/j.cub.2017.09.031

Furstenberg, A., Breska, A., Sompolinsky, H., & Deouell, L. Y. (2015). Evidence of Change of Intention in Picking Situations. *Journal of Cognitive Neuroscience*, *27*(11), 2133–2146. https://doi.org/10.1162/jocn_a_00842

Furstenberg, A., Sompolinsky, H., & Deouell, L. Y. (2023). Error monitoring when no errors are possible: Arbitrary free-choice decisions invoke error monitoring processes. *iScience*, *26*(4), 106373. https://doi.org/10.1016/j.isci.2023.106373

Gläscher, J., Daw, N., Dayan, P., & O'Doherty, J. P. (2010). States versus Rewards: Dissociable Neural Prediction Error Signals Underlying Model-Based and Model-Free Reinforcement Learning. *Neuron*, *66*(4), 585–595. https://doi.org/10.1016/j.neuron.2010.04.016

Guttman, N., & Kalish, H. I. (1956). Discriminability and stimulus generalization. *Journal of Experimental Psychology*, *51*(1), 79–88. https://doi.org/10.1037/h0046219

Icard, T. (2021). *Why Be Random?* https://philarchive.org/rec/ICAWBR

Jeong, H., Taylor, A., Floeder, J. R., Lohmann, M., Mihalas, S., Wu, B., Zhou, M., Burke, D. A., & Namboodiri, V. M. K. (2022). Mesolimbic dopamine release conveys causal associations. *Science*, *378*(6626), eabq6740. https://doi.org/10.1126/science.abq6740

Jocham, G., Brodersen, K. H., Constantinescu, A. O., Kahn, M. C., Ianni, A. M., Walton, M. E., Rushworth, M. F. S., & Behrens, T. E. J. (2016). Reward-Guided Learning with and without Causal Attribution. *Neuron*, *90*(1), 177–190. https://doi.org/10.1016/j.neuron.2016.02.018

Kahnt, T., Park, S. Q., Burke, C. J., & Tobler, P. N. (2012). How Glitter Relates to Gold: Similarity-Dependent Reward Prediction Errors in the Human Striatum. *Journal of Neuroscience*, *32*(46), 16521–16529. https://doi.org/10.1523/JNEUROSCI.2383-12.2012

Klein-Flügge, M. C., Wittmann, M. K., Shpektor, A., Jensen, D. E. A., & Rushworth, M. F. S. (2019). Multiple associative structures created by reinforcement and incidental statistical learning mechanisms. *Nature Communications*, *10*(1), 4835. https://doi.org/10.1038/s41467-019-12557-z

Kool, W., Cushman, F. A., & Gershman, S. J. (2016). When Does Model-Based Control Pay Off? *PLOS Computational Biology*, *12*(8), e1005090. https://doi.org/10.1371/journal.pcbi.1005090

Kyllonen, P. C., & Christal, R. E. (1990). Reasoning ability is (little more than) working-memory capacity?! *Intelligence*, *14*(4), 389–433. https://doi.org/10.1016/S0160-2896(05)80012-1

Leibniz (with unknown library). (1734). *Essais de theodicee sur la bonte de Dieu, la liberte de l'homme, et l'origine du mal, par M. Leibnitz*. chez Francois Changuion. http://archive.org/details/bub_gb_hZVROfS8yZMC

Miller, K. J., Shenhav, A., & Ludvig, E. A. (2019). Habits without values. *Psychological Review*, *126*(2), 292–311. https://doi.org/10.1037/rev0000120

Miranda, B., Butler, J. L., Malalasekera, W. M. N., Behrens, T. E. J., Dayan, P., & Kennerley, S. W. (2025). *Neural signatures of model-based and model-free reinforcement learning across prefrontal cortex and striatum*. https://doi.org/10.1101/2025.01.11.632388

Monsell, S. (2017). Task Set Regulation. In *The Wiley Handbook of Cognitive Control* (pp. 29–49). John Wiley & Sons, Ltd. https://doi.org/10.1002/9781118920497.ch2

Moran, R., Keramati, M., Dayan, P., & Dolan, R. J. (2019). Retrospective model-based inference guides model-free credit assignment. *Nature Communications*, *10*(1), 750. https://doi.org/10.1038/s41467-019-08662-8

Moran, R., Keramati, M., & Dolan, R. J. (2021). Model based planners reflect on their model-free propensities. *PLOS Computational Biology*, *17*(1), e1008552. https://doi.org/10.1371/journal.pcbi.1008552

Niv, Y. (2019). Learning task-state representations. *Nature Neuroscience*, *22*(10), Article 10. https://doi.org/10.1038/s41593-019-0470-8

Niv, Y., Daniel, R., Geana, A., Gershman, S. J., Leong, Y. C., Radulescu, A., & Wilson, R. C. (2015). Reinforcement learning in multidimensional environments relies on attention mechanisms. *Journal of Neuroscience*, *35*(21), 8145–8157. https://doi.org/10.1523/JNEUROSCI.2978-14.2015

Pearce, J. M., & Hall, G. (1980). A model for Pavlovian learning: Variations in the effectiveness of conditioned but not of unconditioned stimuli. *Psychological Review*, *87*(6), 532–552. https://doi.org/10.1037/0033-295X.87.6.532

Rescorla, R., & Wagner, A. (1972). A theory of Pavlovian conditioning: Variations in the effectiveness of reinforcement and non-reinforcement. *Classical Conditioning, Current Research and Theory*, *2*, 64–69.

Rmus, M., Zou, A., & Collins, A. G. (2023). Choice Type Impacts Human Reinforcement Learning. *Journal of Cognitive Neuroscience*, *35*(2), 314–330.

Sher, S., Müller-Trede, J., & McKenzie, C. R. M. (2025). Choices without preferences: Principles of rational arbitrariness. *Psychological Review*. https://doi.org/10.1037/rev0000529

Song, M., Baah, P. A., Cai, M. B., & Niv, Y. (2022). Humans combine value learning and hypothesis testing strategically in multi-dimensional probabilistic reward learning. *PLOS Computational Biology*, *18*(11), e1010699. https://doi.org/10.1371/journal.pcbi.1010699

Talmi, D., Seymour, B., Dayan, P., & Dolan, R. J. (2008). Human Pavlovian–Instrumental Transfer. *Journal of Neuroscience*, *28*(2), 360–368. https://doi.org/10.1523/JNEUROSCI.4028-07.2008

Ullmann-Margalit, E., & Morgenbesser, S. (1977). Picking and Choosing. *Social Research*, *44*(4), 757–785.

Unsworth, N., Fukuda, K., Awh, E., & Vogel, E. K. (2014). Working memory and fluid intelligence: Capacity, attention control, and secondary memory retrieval. *Cognitive Psychology*, *71*, 1–26. https://doi.org/10.1016/j.cogpsych.2014.01.003

Vehtari, A., Gelman, A., & Gabry, J. (2017). Practical Bayesian model evaluation using leave-one-out cross-validation and WAIC. *Statistics and Computing*, *27*(5), 1413–1432. https://doi.org/10.1007/s11222-016-9696-4

Wilson, R. C., Bonawitz, E., Costa, V. D., & Ebitz, R. B. (2021). Balancing exploration and exploitation with information and randomization. *Current Opinion in Behavioral Sciences*, *38*, 49–56. https://doi.org/10.1016/j.cobeha.2020.10.001

Wilson, R. C., & Collins, A. G. (2019). Ten simple rules for the computational modeling of behavioral data. *eLife*, *8*, e49547. https://doi.org/10.7554/eLife.49547

Wilson, R. C., & Niv, Y. (2012). Inferring relevance in a changing world. *Frontiers in Human Neuroscience*, *5*. https://doi.org/10.3389/fnhum.2011.00189

Reviewer #1 (Remarks to the Author):

The authors did an impressive job in addressing my concerns. They collected additional data where the original datasets were insufficient for addressing some of the issues I raised, they added a set of new analyses that specifically address the outstanding points and revised their manuscript accordingly. In my view, the new results substantially strengthen the evidence supporting the main claims. I am happy to now recommend publication in Nature Communications.

We sincerely thank the reviewer for their rigorous and constructive feedback, which has substantially strengthened the manuscript.

One minor point: I would suggest standardising the y-axis limits to facilitate comparison of effect sizes across stimulus dimensions, e.g. in Fig. 4B and 4D or Fig. 5D-G.

Thank you. This was now fixed.

Reviewer #2 (Remarks to the Author):

In my opinion the authors have done a thorough and convincing job of addressing/removing the concerns raised about the previous version of this manuscript. This was a good deal of work, I'm sure, but the evidence from the new experiments and analyses makes the paper much stronger now. I also appreciate the author's willingness to clarify some of the conceptual framing and claims in the paper.

We sincerely thank the reviewer for their critical, constructive, and high-quality review, and we deeply appreciate the time and effort they invested in evaluating our manuscript.

In their reply to my comment about the labeling of outcome-irrelevant learning as model-free and the corresponding implicit claim that the outcome-irrelevant learning is limited to a model-free system they stated that agreed with the point and, "We now only refer to “model-free” in a dedicated discussion paragraph that addresses the nuances around model-free processing, as can be seen below. Critically, we assert that demonstrating automatic assignment of value to outcome-irrelevant action features is a meaningful contribution to the field of human learning and decision making, regardless of whether outcome-irrelevant learning is predominantly model-free or model-based. "

I think this discussion paragraph is important and I agree with the assertion that contribution is meaningful regardless of where outcome-irrelevant learning comes from. That said, the label 'Outcome-irrelevant model-free learning' still appears 5 times in the revised manuscript. I assume this is an oversight on the authors' part given their statement in the rebuttal letter.

We thank the reviewer for catching this slight oversight. We have very carefully checked the manuscript for any remaining leftovers of this label and made sure none remain.

In addition to the 5 remaining labels the following sentence from the paper directly claims that a model-free system is responsible for the outcome-irrelevant learning. "This finding supports the conclusion that outcome-irrelevant learning reflects an inherent component of human model-free reinforcement learning, rather than a misunderstanding of the task structure." As we seem to agree that the data cannot establish if outcome-irrelevant learning is model-based/free or something else altogether, I ask the authors to revise the sentence to read, "This finding supports the conclusion that outcome-irrelevant learning reflects an inherent component of human reinforcement learning, rather than a misunderstanding of the task structure." To be clear, it is still very important to show that outcome-irrelevant learning is not driven (solely) by confusion about the task structure. I simply want to push the field in general away from "automatically" lumping sub-optimal behaviors onto a model-free system without clear evidence for its involvement.

We appreciate the reviewer catching this sentence and agree with their theoretical perspective, and we have revised the manuscript to include the suggested phrasing (see lines 363 to 365 in the main text).

Other than these minor modifications to the text, I think the current version of the manuscript makes important contributions to the field.

We once again thank the reviewer for their valuable time and constructive feedback.
